# Discovery and biosynthesis of tricyclic copper-binding ribosomal peptides containing histidine-to-butyrine crosslinks

**DOI:** 10.1038/s41467-023-38517-2

**Published:** 2023-05-23

**Authors:** Yuqing Li, Yeying Ma, Yinzheng Xia, Tao Zhang, Shuaishuai Sun, Jiangtao Gao, Hongwei Yao, Huan Wang

**Affiliations:** 1 State Key Laboratory of Coordination Chemistry, Chemistry and Biomedicine Innovation Center of Nanjing University, Jiangsu Key Laboratory of Advanced Organic Materials, School of Chemistry and Chemical Engineering, Nanjing University, Nanjing, 210093 China; 2 State Key Laboratory of Ecological Pest Control for Fujian and Taiwan Crops, College of Life Sciences, Fujian Agriculture and Forestry University, 350002 Fuzhou, China; 3 Institute of Molecular Enzymology, School of Biology and Basic Medical Sciences, Soochow University, Suzhou, 215123 China

**Keywords:** Biosynthesis, Enzyme mechanisms, Peptides, Enzymes, Natural product synthesis

## Abstract

Cyclic peptide natural products represent an important class of bioactive compounds and clinical drugs. Enzymatic side-chain macrocyclization of ribosomal peptides is a major strategy developed by nature to generate these chemotypes, as exemplified by the superfamily of ribosomally synthesized and post-translational modified peptides. Despite the diverse types of side-chain crosslinks in this superfamily, the participation of histidine residues is rare. Herein, we report the discovery and biosynthesis of bacteria-derived tricyclic lanthipeptide noursin, which is constrained by a tri amino acid labionin crosslink and an unprecedented histidine-to-butyrine crosslink, named histidinobutyrine. Noursin displays copper-binding ability that requires the histidinobutyrine crosslink and represents the first copper-binding lanthipeptide. A subgroup of lanthipeptide synthetases, named LanKC_Hbt_, were identified to catalyze the formation of both the labionin and the histidinobutyrine crosslinks in precursor peptides and produce noursin-like compounds. The discovery of the histidinobutyrine-containing lanthipeptides expands the scope of post-translational modifications, structural diversity and bioactivity of ribosomally synthesized and post-translational modified peptides.

## Abstract (web summary)

Cyclic peptides are important bioactive compounds and drugs, synthesised by enzymatic side-chain macrocyclization of ribosomal peptides, which rarely involves histidine residues. Here, the authors report the discovery and biosynthesis of tricyclic lanthipeptide noursin, constrained by a tri amino acid labionin crosslink and histidine-to-butyrine crosslink, which is important for copper binding of noursin.

## Introduction

Cyclic peptides possess structural and functional features that fill the gap between small molecules and large biologics and therefore hold great potential as a distinct class of therapeutics^1^. In addition to the well-documented N-to-C-terminal amide bond formation^2–4^, post-translational side-chain macrocyclization is a major strategy to generate cyclic peptide natural products, as demonstrated by the superfamily of ribosomally synthesized and post-translationally modified peptides (RiPPs)^5–7^. The biosynthesis of RiPPs is usually initiated by the production of a ribosomal precursor peptide composed of an N-terminal leader peptide (LP) and a C-terminal core peptide (CP), which subsequently undergoes enzymatic modifications during biosynthesis, including side-chain macrocyclization. Representative side-chain crosslinks in RiPPs include thioether bonds (lanthipeptides^8^, sactipeptides^9^ and ranthipeptides^10^), substituted pyridines (thiopeptides^11,12^), aryl-C(sp^3^) bonds (streptide^13^ and darobactin^14,15^), aryl-oxygen bonds (dikaritins^16–18^), which are generated through distinct enzymatic transformations. For example, lanthipeptides are typically synthesized through the dehydration of Ser/Thr residues to generate dehydroalanine (Dha)/dehydrobutyrine (Dhb), respectively, and the subsequent macrocyclization between cysteine and Dha/Dhb to form methyllanthionine (MeLan) or methyllabionin (MeLab) crosslinks^8^.

**Fig. 1:**
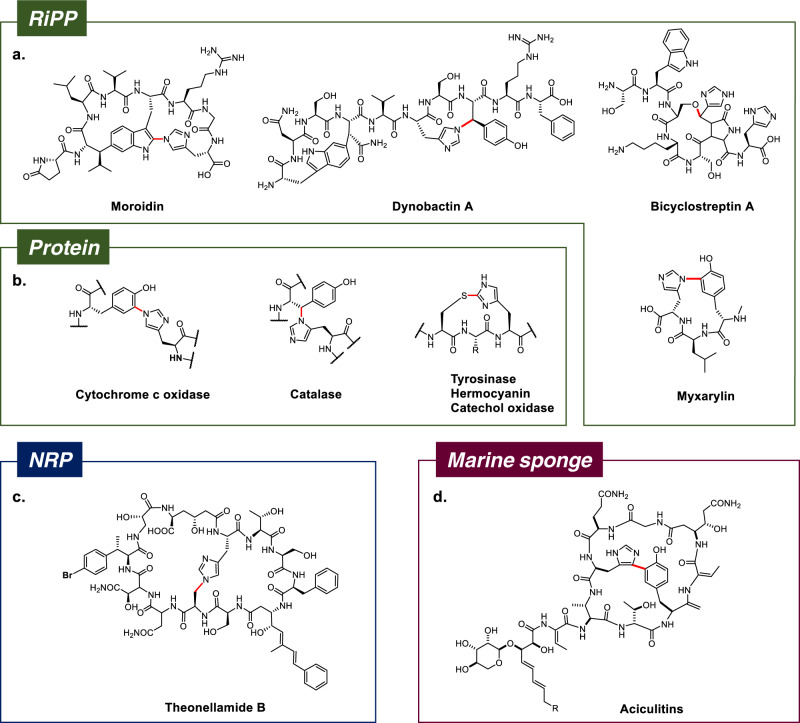
Cyclic peptide natural products and proteins with His-containing crosslinks. **a** RiPP cyclic peptides with His-containing crosslinks. **b** His-containing crosslinks in proteins generated via PTM. **c** NRP theonellamide B. **d** Marine sponge-derived aciculitins. RiPP ribosomally synthesized and post-translationally modified peptide; NRP non-ribosomal peptide. His-containing crosslinks are highlighted in red.

Histidine-containing crosslinks are relatively rare in cyclic peptide natural products. Examples from the RiPP family include the plant-derived moroidin-type bicyclic peptides^19–21^, dynobactin A^22^, bicyclostreptin^23^, and biarylitides^24,25^ (Fig. 1a), in which the His-containing crosslinks are generated post-translationally by either copper-dependent BURP cyclases, radical *S*-adenosylmethionine (rSAM) enzymes or cytochrome P450 enzymes. His-containing crosslinks are also found in proteins, such as the His(Nɛ)-Tyr(C6) crosslink in cytochrome c oxidases^26,27^, the His(Nδ)-Tyr(Cβ) crosslink in catalase HP II^28^, the His(Cɛ)-Cys(S) crosslinks in tyrosinases^29^, hemocyanins^30^ and catechol oxidases^31^ (Fig. 1b), most of which are generated post-translationally via metal-dependent autocatalytic processes. In addition, non-ribosomal peptides theonellamides contain histidinoalanine crosslinks (Fig. 1c)^32^, and marine sponge-derived aciculitins contain His-Tyr crosslinks (Fig. 1d)^33,34^. However, corresponding crosslinking mechanisms remain uncharacterized.

**Fig. 2:**
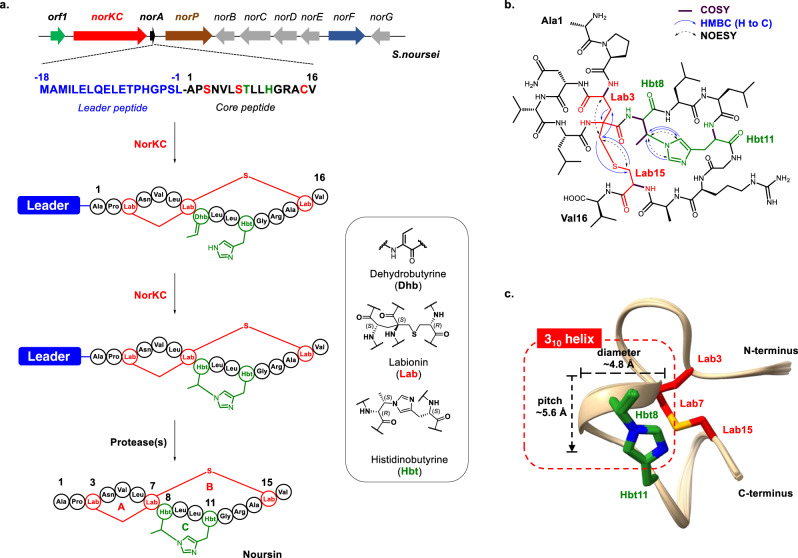
Biosynthesis and the chemical structure of noursin. **a** The biosynthesis of noursin. **b** The chemical structure of noursin. The 2D NMR correlations of the Lab and the Hbt crosslink are shown. **c** Ensemble of the 20 lowest energy conformers derived from 2D NMR analysis depicting all backbone atoms indicates the presence of a short 3_10_-helix. The Lab and His crosslinks are highlighted in red and green, respectively.

Herein, we report the discovery, characterization and biosynthesis of bacteria-derived tricyclic copper-binding lanthipeptides with both Lab crosslinks and unprecedented histidinobutyrine (Hbt) crosslinks, in which the His(Nɛ) is covalently conjugated to the Cβ of a butyrine residue (Abu(Cβ)) (Fig. 2b). A subfamily of lanthipeptide synthetases, named LanKC_Hbt_, with unique cyclase domains are revealed to catalyze the formation of both Lab and Hbt macrocycles through step-wise Michael-type addition reactions. This study reveals the Hbt crosslink as a new type of post-translational modification (PTM) in RiPP biosynthesis and provides insights into the remarkably promiscuous catalytic functions of RiPP synthetases.

## Results

### Discovery of the lanthipeptide noursin with a Hbt crosslink

Through genome mining, we identified a putative lanthipeptide biosynthetic gene cluster (BGC) spanning 12 kb from the strain of *Streptomyces noursei* ATCC 11455, named the *nor* gene cluster (Fig. 2a). The *nor* BGC consists of genes encoding a putative class III lanthipeptide synthetase NorKC, a precursor peptide NorA, a S9 family peptidase NorP, and a putative transporter NorF (Fig. 2a and Supplementary Table 1). The precursor peptide NorA contains a LELQEL motif in its N-terminal segment, which is highly conserved in the leader peptides (LPs) of class III lanthipeptides as the substrate recognition element for modification enzymes^8,35–37^. The putative C-terminal core peptide (CP) of NorA (NorA_CP_) contains three Ser/Thr residues as potential dehydration sites and one Cys residue. The peptidase NorP is likely responsible for the removal of the LP after the completion of modifications^38–40^. Other putative enzymes in the *nor* BGC are rarely found in lanthipeptide BGCs, and their functions are unclear.

To explore the function of the *nor* BGC, we prepared a cosmid library of *S. noursei* ATCC 11455 genomic DNA and obtained a cosmid containing the complete *nor* gene cluster, denoted as pNOR (Supplementary Fig. 1a). Heterologous expression of the pNOR cosmid in *S. lividans* TK24 yielded a peptide product in the liquid culture medium, named noursin, with a mass of [M + 2H]^2+^ *m/z* = 792.4254, which matched with the product of the putative NorA_CP_ after threefold dehydration (mass error of 2.4 ppm) (Supplementary Fig. 1). Noursin was then purified by XAD16 nonionic macroporous resin, reverse phase silica gel column and high-performance liquid chromatography (HPLC) (Supplementary Fig. 2). Marfey’s analysis of noursin revealed the presence of Gly and L-enantiomers of Ala, Pro, Asn, Val, Leu, and Arg, matching with the sequence of the putative NorA_CP_ (Supplementary Fig. 3). Ser and Thr derivatives were not detected by the Marfey’s analysis, which is in agreement with the proposed threefold dehydration of NorA_CP_ during noursin biosynthesis. Treatment of noursin with the thiol-selective reagent iodoacetamide (IAA) and 2-hydroxy-1-ethanethiol (βME) resulted in no modification, indicating the absence of free Cys or Dha/Dhb residues (Supplementary Fig. 4a). Tandem mass spectrometry (MS/MS) analysis of noursin suggested the presence of a ring structure between residues Ser3 and Cys15 (Supplementary Fig. 4b). As the ratios of Dha/Dhb and Cys residues involved in the formation of a MeLan and MeLab motif are typically 1:1 and 2:1^8^, respectively, the consumption of three Dha/Dhb and one Cys residue during noursin biosynthesis implies the presence of PTM(s) other than (Me)Lan/(Me)Lab formation.

The structure of noursin was further characterized by a suite of two-dimensional NMR spectra, including ^1^H-^1^H COSY, TOCSY, NOESY, and ^1^H-^13^C HSQC, HMBC and ^1^H-^15^N HSQC (Supplementary Figs. 5–10, Supplementary Tables 2, 3). The 3rd, 7th and 15th residues to the N-terminus of noursin showed Cβ or/and Cα chemical shifts with significant differences with typical Ser and reduced Cys residues. The 2D ^1^H-^13^C HMBC spectrum clearly showed the inter-residue crosspeaks of Lab3(Cβ)-Lab7(Hβ), Lab7(Cα/β)-Lab3(Hβ), Lab7C’(the carbonyl carbon)-Lab3(Hβ), Lab7(Cβ)-Lab15(Hβ) and Lab15(Cβ)-Lab7(Hβ) (Supplementary Fig. 9), indicating the formation of a Lab crosslink via the Lab3(Cβ)-Lab7(Cα) bond and the Lab7(Cβ)-Lab15(S) bond. In addition, no Hα signal was observed for the 7th residue, indicating that the 7th residue is the central amino acid of the Lab motif with a quaternary Cα atom. To determine the configuration of this Lab crosslink, we utilized labyrinthopeptin A2 as a standard, which contains a (*2S*,*4S*,*8R*)-labionin crosslink. The biosynthesis of labyrinthopeptin A2 was reconstituted in vitro following a reported protocol, in which the precursor peptide LabA2 was modified by enzyme LabKC^41^. Noursin and the LabKC-modified LabA2 were then hydrolyzed, derivatized and subjected to LC-MS analysis. Results showed that the Lab derivatives generated from noursin and the LabKC-modified LabA2 peptide co-eluted with the same retention time, indicating that the Lab crosslink in noursin adopts a (*3S*,*7S*,*15R*) configuration (Supplementary Fig. 11).

The Cβ of the 8th residue displayed a chemical shift of 56.6 ppm, which is significantly larger than that reported in the Cβ of a Abu residue in MeLan (~45 ppm)^41^. Meanwhile, inter-residue crosspeaks of the Cβ of the 8th residue and His11(Hδ2/ε1), as well as of the His11(Cδ2/ε1) and the Hβ of the 8th residue were observed in the 2D ^1^H-^13^C HMBC spectra, indicating the formation of a Hbt crosslink between the Cβ of the 8th residue and His11(Nε2) (Fig. 2b and Supplementary Fig. 12). The conjugation with the histidine imidazole presumably results in a less electron shielded Hbt8(Cβ) with a characteristic chemical shift. Finally, the abundant Lab3-to-Lab7, Lab7-to-Lab15, and Hbt8-to-Hbt11 NOE peaks further support the formation of a Lab and a Hbt motif in noursin (Supplementary Fig. 7).

To validate the abovementioned structural assignment, we expressed a pNOR_H11W_ cosmid carrying NorA_H11W_ as the precursor peptide in *S. lividans* TK24 and acquired compound noursin_H11W_ (Supplementary Fig. 13). NMR analysis of noursin_H11W_ clearly showed the signals of unmodified Dhb8 and Trp11 residues (Supplementary Figs. 14–18). In contrast, the ^1^H NMR signals of Dhb8 and His11 residues are both missing in the NMR spectra of noursin. To chemically verify the presence of a Hbt crosslink, noursin was hydrolyzed, derivatized to corresponding *N*-trifluoroacetyl ethylesters and analyzed by LC-HRMS (Supplementary Fig. 19). Molecular ions matching the corresponding derivatives of Lab and Hbt crosslinks were both detected. These data further supported the formation of a Lab and a Hbt crosslink in noursin.

### Computational 3D structures of noursin

To elucidate the three-dimensional structure of noursin, extensive crystallographic attempts were made, but no crystal of sufficient quality for diffraction was acquired. Thus, three-dimensional conformations of noursin were modeled using ^1^H-^1^H distance constraints derived from 2D ^1^H-^1^H NOESY spectra. The Xplor-NIH software package was used for the structure calculation with the configuration of the Lab crosslink set as *3S*,*7S*,*15R*^42^. Four possible configurations of the Hbt8 residue, including (*S*)*-*Cα-(*S*)*-*Cβ, (*S*)*-*Cα-(*R*)*-*Cβ, (*R*)*-*Cα-(*S*)*-*Cβ and (*R*)*-*Cα-(*R*)*-*Cβ, were subjected to the structural calculation. Only the noursin structure with the Hbt8 in the (*R*)*-*Cα-(*S*)*-*Cβ configuration fits the NOESY-derived restraints and predicted energy very well, whereas the other three possible configurations all caused significant violations and created very high potential energies (Supplementary Table 5 and Supplementary Table 6). Meanwhile, Hbt8(Hβ) and Hbt11(Hδ2) protons exhibited a stronger NOE correlation than that between Hbt8(Hβ) and Hbt11(Hε1), suggesting that the Hbt8(Hβ) is in a shorter distance with Hbt11(Hδ2) than that with Hbt11(Hε1), which agreed with the *S* configuration assignment of Hbt8(Cβ) (Supplementary Fig. 20).

Superposition of the 20 lowest energy conformers illustrated that noursin adopts highly compact and globular conformations with a rigid and ordered backbone structure (Fig. 2c and Supplementary Fig. 21). The A ring of the Lab motif (residues Lab3–Lab7 as depicted in Fig. 2a) is highly constrained in an unusual conformation with a positive phi (*ϕ*) dihedral angle value of residue Asn4, and unusual psi (*ψ*) dihedral angle values of residues Asn4 and Leu6 (Supplementary Table 7). The highly distorted conformation of ring A is stabilized by a hydrogen bonding network formed among the carbonyl oxygen atoms of Lab3 and the nitrogen atoms of Val5, Leu6 and Lab7 (Supplementary Fig. 23a). The Hbt crosslink forces the backbone of the C ring into a conformation with unfavorable phi (*ϕ*) and psi (*ψ*) dihedral angle values on residues Hbt8 and Leu9 (Fig. 2a and Supplementary Table 7). Importantly, Hbt11(Nδ) is in a position to interact with Lab15(S) as well as with the amide nitrogen and the C-terminal carboxylate group of Val16 by forming a unique noncovalent bonding network (Supplementary Fig. 23b). Intriguingly, the backbone amides of residues Leu6, Lab7, Leu9 and Leu10 form a hydrogen bond network that resembles a 3_10_ helix with a pitch of ~5.6 Å and a radius of ~2.4 Å (Fig. 2c). The presence of a helical structure unit was further supported by the characteristic CD spectrum of noursin (Supplementary Fig. 24). Overall, with the participation of a Hbt crosslink via covalent and noncovalent bonding, noursin is locked in a highly constrained and globular conformation.

### Noursin is a copper binding lanthipeptide

**Fig. 3:**
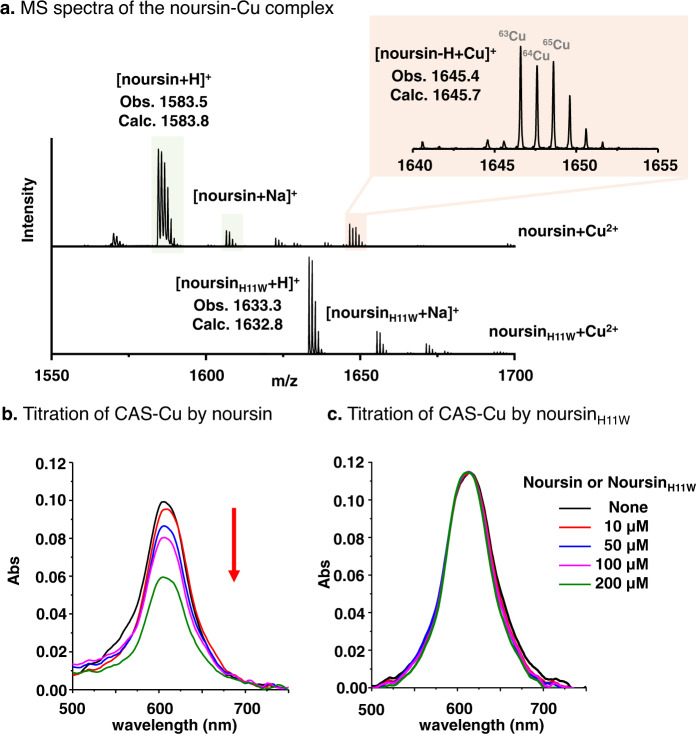
The copper-binding activity of noursin is dependent on the Hbt crosslink. **a** MALDI-TOF mass spectra of the samples of noursin and noursin_H11W_ with CuSO_4_. **b** UV–Vis spectra of a CAS-Cu solution titrated by noursin. **c** UV–Vis spectra of a CAS-Cu solution titrated by noursin_H11W_. Assay conditions: a solution of 50 μM CAS, 100 μM HDTMA and 8 μM CuSO_4_ in 20 mM HEPES buffer was titrated by noursin or noursin_H11W_ and incubated for 10 min before analyzed by a UV–Vis spectrometer.

Class III lanthipeptides possess diverse biological functions including antibacterial activities^35,37,41,43–46^. Preliminary bioactivity evaluation of noursin showed no antimicrobial activities against a limited collection of strains, including *Escherichia coli*, *Pseudomonas aeruginosa*, *Salmonella* and *Staphylococcus aureus*. Next, we evaluated the interaction of noursin with metal ions, including Fe^3+^, Al^3+^, Cu^2+^, Zn^2+^, Ni^2+^, Mg^2+^, and Mn^2+^ ions. Only the solution of noursin and CuSO_4_ yielded a new compound with a single charge and m/z 1645.4, as detected by MALDI–TOF–MS analysis (Fig. 3a and Supplementary Fig. 25a). The observed m/z and the distinct isotopic distribution match with the singly charged ion of a noursin-Cu complex (Fig. 3a). The binding between noursin and Cu ions was further confirmed by titrating a solution of CAS and CuSO_4_ by noursin in 20 mM HEPES buffer, pH 7.5. The addition of noursin gradually decreased the characteristic absorbance of the CAS-Cu complex at 618 nm, indicating the Cu-chelating property of noursin (Fig. 3b). Electro paramagnetic resonance analysis of the mixture of noursin and CuSO_4_ showed that the characteristic signal of Cu(II) is not affected by the varied ratio of noursin to CuSO_4_, suggesting that the complex is in a noursin-Cu(II) form (Supplementary Fig. 26). Copper-binding peptide natural products are rare in nature, and the only reported RiPP chalkophores are methanobactins derived from methanotrophic bacteria^47–49^. Thus, noursin represents the first copper-binding lanthipeptide.

Next, we evaluated the impact of structural units of noursin to its Cu-binding activity. Noursin_H11W_, which lacks the Hbt crosslink, displayed no Cu-binding activity (Fig. 3c and Supplementary Fig. 25b), showing that the Hbt motif is an essential structural unit for the Cu-binding activity of noursin. Neither NorA peptide nor the NorKC-modified NorA peptide formed chelating complex with Cu ions, indicating that the modification by NorKC and the leader removal are required (Supplementary Fig. 25c).

### NorKC catalyzes the formation of the Lab and Hbt motifs

**Fig. 4:**
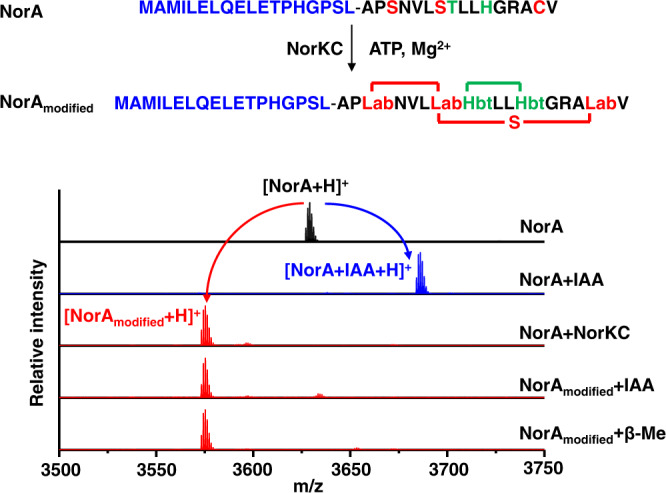
NorKC catalyzes the formation of the Lab and the Hbt motif in NorA. MALDI-TOF-MS analysis of the modification of NorA by NorKC. NorA: *M*_calc._=3627.87 Da, *M*_obs._=3627.12 Da; NorA_modified_: *M*_calc._=3573.84 Da, *M*_obs._=3573.29 Da; NorA-IAA adduct: *M*_calc._=3684.89 Da, *M*_obs_ = 3684.08 Da. Assay conditions: Tris-HCl buffer (pH 8.0), 1 mM NTPs, 1 mM MgCl_2_, 1 mM TCEP, 50 μM NorA and 10 μM NorKC at 28 °C for 1 h. Residues involved in Lab and Hbt formation in NorA peptide are in red and green, respectively.

To explore the biosynthetic origin of the Hbt crosslink, we examined the role of each putative enzyme encoded in the *nor* BGC through gene deletion in the pNOR cosmid. Only the *norA* and *norKC* genes are strictly required for the heterologous production of noursin in *S. lividans* TK24 (Supplementary Fig. 27), implying that NorKC is responsible for the formation of both the Lab and Hbt motifs. NorKC was then expressed as a fusion protein with an N-terminal His_6_-tag in *E. coli* and purified by immobilized metal ion affinity chromatography (IMAC). Synthetic NorA peptide was employed as a substrate to characterize the function of NorKC in vitro. In the presence of cofactor ATP/CTP/GTP and Mg^2+^ ions, NorKC efficiently converted NorA to a threefold dehydrated peptide product (denoted as NorA_modified_), as determined by MALDI-TOF-MS analysis (Fig. 4 and Supplementary Fig. 28). Incomplete dehydration of NorA by NorKC was observed when TTP was supplied as a cofactor (Supplementary Fig. 28). Treatment of NorA_modified_ by IAA and βMe did not result in any mass change, indicating that Cys15 and the three Dha/Dhb motifs are all consumed during the in vitro enzymatic modification (Fig. 4). Tandem MS analysis of NorA_modified_ showed no fragmentation between the C-terminal residues Ser3 and Val16, suggesting the formation of ring structures (Supplementary Fig. 29). NorA_modified_ was further hydrolyzed by HCl, derivatized and analyzed by LC-MS. Both derivatives of the Lab and Hbt crosslinks were detected with the same retention times as those derived from noursin produced via the fermentation of *S. lividans* TK24/pNOR (Supplementary Fig. 19), validating the correct installation of a Lab and a Hbt motif by NorKC in vitro. Thus, NorKC is capable of installing two distinct types of macrocycles in its precursor peptide through the formation of a C-C bond, a C-S bond, and a C-N bond.

### Formation of the Lab and Hbt motifs by NorKC is stepwise and leader-dependent

**Fig. 5:**
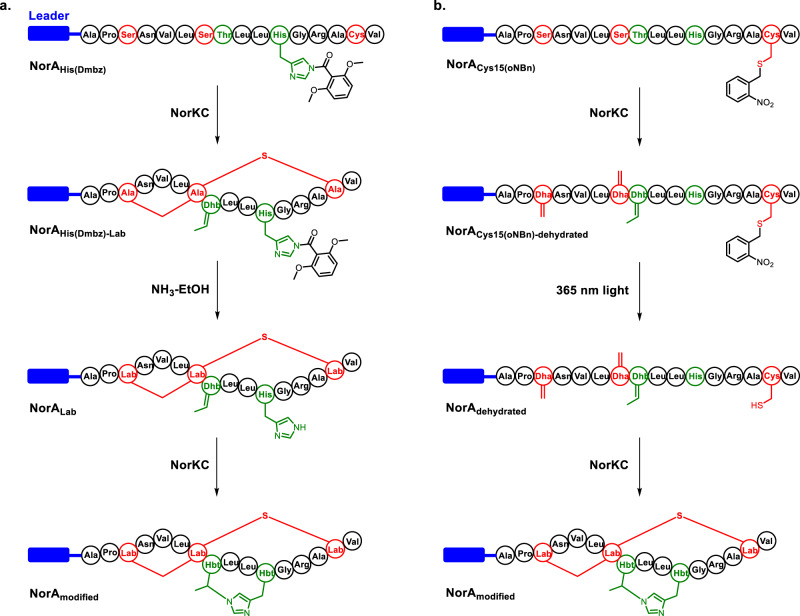
NorKC catalyzes the formation of the Lab and the Hbt motif in a stepwise manner. NorA_His(Dmbz)_ (**a**) and NorA_Cys15(oNBn)_ (**b**) were modified by NorKC, deprotected and modified by NorKC again to complete macrocyclization. Residues involved in Lab and Hbt formation in NorA peptide are in red and green, respectively. The leader peptide of NorA is represented as blue rectangle.

To explore the order of the formation of the Lab and Hbt motifs in noursin, we synthesized two precursor peptide variants with the side-chain of His11 or Cys15 protected. NorA_His(Dmbz)_ contains a residue His11 with the Nε masked by a 2,6-dimethoxybenzoyl group (Fig. 5a). Modification of NorA_His(Dmbz)_ by NorKC resulted in the product NorA_His(Dmbz)-Lab_, which is not modified by IAA but susceptible to βME addition (Fig. 5a, Supplementary Fig. 30). NorA_Lab_ was then generated by the deprotection of NorA_His(Dmbz)-Lab_ by the ammonolysis reagent (Fig. 5a), desulfurized by NiCl_2_ and NaBH_4_ and analyzed by MSMS. Results showed the formation of the Lab motif but not the Hbt motif in the linearized NorA_Lab_ (Supplementary Fig. 31). Furthermore, NorA_Lab_ was fully hydrolyzed by HCl, and the resulting amino acids were derivatized to corresponding *N*- trifluoroacetic acid ethyl esters before LC-HRMS analysis. Only the Lab derivative was detected by LC-MS but not the Hbt derivative (Supplementary Fig. 32a). Therefore, in the absence of a reactive His residue, NorKC specifically catalyzes the formation of a Lab crosslink in the NorA peptide. Next, NorA_Lab_ was incubated with NorKC in the absence of ATP and Mg^2+^, which led to the formation of NorA_modified_ with the Hbt motif installed successfully (Fig. 5a and Supplementary Figs. 30 and 32b). This result indicates that the Hbt formation occurs via the NorKC-catalyzed Michael-type addition of His11 to Dhb8.

As a second peptide substrate, NorA_Cys15(oNBn)_ with an *o*-nitrobenzyl protected residue Cys15 was incubated with NorKC, which generated fully dehydrated peptide NorA_Cys15(oNBn)-hydrated_ without macrocyclization (Fig. 5b and Supplementary Fig. 33). NorA_Cys15(oNBn)-dehydrated_ was then irradiated by 365 nm light to remove the *o*-nitrobenzyl group on Cys15 and subjected to the reaction with NorKC. As expected, fully cyclized NorA_modified_ was generated near quantitatively under the assay condition. Collectively, these data demonstrated that NorKC catalyzes the two types of macrocyclizations in a stepwise manner, where the formation of the Lab motif occurs prior to the Hbt motif.

The modification of lanthipeptide precursor peptides usually requires the LPs as a substrate recognition element for the modification enzymes^50–53^. Indeed, NorA_CP_ was barely modified by NorKC under assay conditions, and supplementation of NorA_LP_ in trans had no observable effect in enhancing the modification efficiency (Supplementary Fig. 34). The leader-dependence of the Hbt formation was specifically examined by employing peptide NorA_Lab_(−9)−16_ as a substrate, in which the N-terminal eight residues of NorA_LP_ was truncated (Supplementary Fig. 35a). Results showed that NorKC was not capable of catalyzing the Hbt formation in NorA_Lab_(−9)−16_ (Supplementary Fig. 35b). Thus, the installation of both the Lab and Hbt motifs by NorKC in NorA peptide is leader-dependent.

### The Lab formation increases the reactivity of Dhb8

The Michael addition of histidine or imidazole to a Dhb residue does not occur spontaneously under aqueous conditions (Supplementary Fig. 36), likely due to the weak nucleophilicity of imidazole and the steric hindrance of the β-methyl group in Dhb. Even with the strong nucleophile βME, residue Dhb8 in the dehydrated linear NorA_S3A_S7A_C15A_ peptide was unreactive in 20 mM Tris-HCl buffer, pH 8.0, within 5 h (Supplementary Fig. 37a). In contrast, the Dhb8 residues in the cyclized peptides NorA_Lab_ and NorA_Lab_H11A_ was modified by βME with significantly increased efficiency, generating corresponding adducts in 25% and 38% yields, respectively, within 1 h and >70% conversion after 5 h (Supplementary Fig. 37b and c). These results indicate that the reactivity of the Dhb8 residue was increased after the formation of the Lab ring. The NMR structure of noursin_H11W_ shows that the C’-Cα-Cβ bond angle of Dhb8 is 115.6°, which is between a typical sp^2^ bond angle of 120°and the Lab8(Cα)(sp^3^) bond angle of 109.6° in noursin (Supplementary Fig. 38). Thus, the enhanced reactivity of Dhb8 in NorA_Lab_ as a Michael acceptor is likely due to local structural distortion introduced by the Lab ring formation. An activated Dhb in NorA_Lab_ peptide may facilitate the formation of the Hbt crosslink and also contribute to the order of ring formation. It is worth noting that the enzymatic catalysis by NorKC is still critical for the Hbt formation despite the increased reactivity of Dhb8, as the Hbt crosslink cannot be generated by incubation of NorA_Lab_ without NorKC under assay conditions.

### Substrate tolerance of NorKC

To explore the chemoselectivity of NorKC during macrocyclization, residues His11 and Cys15 were mutated to various nucleophilic amino acids (Table 1). Modification of NorA_C15H_ by NorKC resulted in a threefold dehydrated peptide product with no ring structure, indicating that NorKC strictly requires a Cys residue for the Lab formation (Supplementary Fig. 39). Mutations of His11 to D-His, Lys or Trp residues resulted in no crosslinking between the 8th and 11th residues despite the formation of a Lab ring (Supplementary Figs. 40, 41), showing the stereo- and chemoselectivity of NorKC for the Hbt formation. Interestingly, when Thr8 was mutated to Ala or Ser, the correct Lab formation was interrupted. NorKC-modified NorA_T8A_ contains a Dha7-Cys15 Lan crosslink, whereas NorKC-modified NorA_T8S_ contains a Dha8-Cys15 Lan crosslink, indicating that NorKC is sensitive to the alteration at the 8th residue for the Lab cyclization (Supplementary Figs. 42, 43). To probe the versatility of NorKC to install Hbt crosslinks in cyclic peptide substrates, NorA_S3A_ and NorA_S7A_ were synthesized and modified by NorKC. Results showed that a Lan crosslink was formed between 7th−15th and 3rd−15th residues in NorKC-modified NorA_S3A_ and NorA_S7A_, respectively (Table 1, Supplementary Figs. 44,45). Hbt crosslinks were successfully installed in both peptides, indicating that NorKC accepts peptide substrates with cyclic structures other than a (*3*,*7*,*15*)-Lab ring. In addition, NorA_L9F_ and NorA_insert8A_ were both modified by NorKC to yield tricyclic peptide containing Hbt crosslinks (Supplementary Figs. 46, 47), showing that amino acid alterations in the Hbt ring and the Lab ring could be tolerated by NorKC. It is noteworthy that although Hbt crosslinks were formed in NorKC-modified NorA_S3A_, NorA_S7A_ and NorA_insert8A_, LC-MS analysis showed that the stereo-specificity of the Hbt crosslinks was compromised (Supplementary Figs. 44, 45,47). These results indicate that the Lan or Lab ring, in which the Dhb and His residues are located, may have an impact on the configurations of the resulting Hbt crosslink.

**Table 1:**
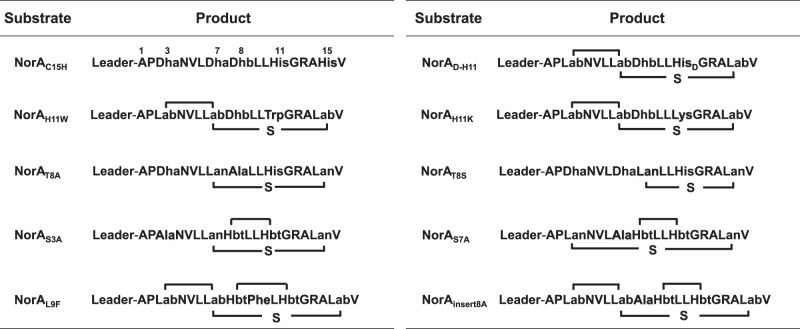
Modification of various NorA mutants by NorKC

Mutations were highlighted with shadings.

### Prevalence of the *nor*-like BGCs

**Fig. 6:**
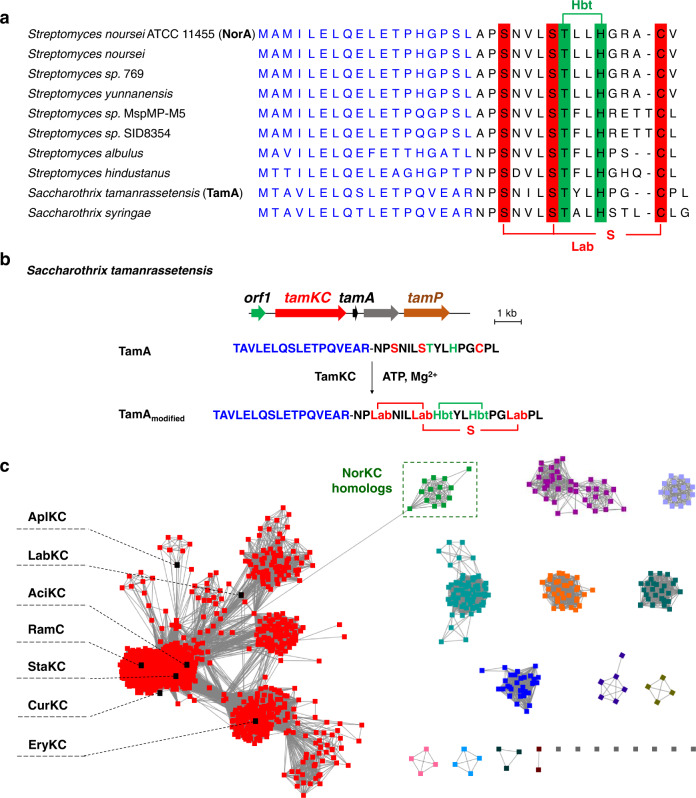
Genome mining and sequence-function analysis of NorKC homologs. **a** Precursor peptides in *nor*-like BGCs. **b** The *tam* BGC and the modification of TamA by TamKC in vitro. **c** SSN analysis of class III lanthipeptide synthetases reveals that NorKC homologs belong to a separate cluster distinct from typical LanKC enzymes. The leader peptides of NorA-like precursor peptides are in blue. Putative residues involved in Lab and Hbt formation are in red and green, respectively.

Intrigued by the unique multicyclic structure of noursin, we investigated the prevalence of the *nor*-like BGCs in bacterial genomes. Genome mining of bacterial genomes led to the identification of ten homologous BGCs in *Streptomyces* and *Saccharothrix* strains (Fig. 6a and Supplementary Fig. 48). The *tam* BGC from *Saccharothrix tamanrassetensis* and the *abl* BGC from *S. albulus*as were selected as representative examples to verify their biosynthetic potential. AlbKC and TamKC share 96% and 59% sequence similarity with NorKC, respectively, and precursor peptides AblA and TamA share high homology with NorA in both leader and core peptides. In vitro enzymatic experiments showed that AblKC and TamKC efficiently modified AblA and TamA, respectively, with ATP and Mg^2+^ by generating the corresponding cyclic peptide products with Lab and Hbt motifs (Fig. 6b and Supplementary Figs. 49, 50), supporting the function of the *nor*-like BGCs in producing noursin-like tricyclic peptides.

Functional validation of *nor*-like BGCs promoted us to analyze the sequence feature for these unique lanthipeptide synthetases. A protein sequence similarity network (SSN) was generated using the EFI-EST tool^54^ with two thousand selected class III lanthipeptide synthetases (LanKCs) from the NCBI database. Results revealed that NorKC homologs clustered into a separate subgroup, which is distinguishable from the characterized LanKCs, including AplKC from NAI-112 biosynthesis and RamC from SapB biosynthesis (Fig. 6c). Detailed SSN analysis of the lyase domains, the kinase domains and the cyclase domains of these LanKCs showed that their kinase and lyase domains are highly homologous (Supplementary Fig. 51). In contrast, the cyclase domains of NorKC homologs are grouped in a separate cluster with a distal phylogenetic relationship to characterized LanKC enzymes (Supplementary Fig. 51). The sequence analysis is in good agreement with the experimental observation that NorKC homologs exhibit unique cyclase activities to utilize both Cys and His for macrocyclization, whereas their dehydratase activities follow the catalytic routine of typical LanKC enzymes.

## Discussion

Common post-translational modifications of histidine residues, including methylation and phosphorylation, often rely on the nucleophilicity of the imidazole side-chain and a highly activated cofactor, such as S-adenosyl-L-methionine (SAM) and ATP^55–57^. Probably due to the lack of electrophilicity in proteinogenic amino acids, enzymatic side-chain crosslinking in peptides and proteins rarely utilize His residues as nucleophiles. Known examples of His-containing crosslinks in proteins are usually generated through metal-dependent catalysis^58^. In this study, we reveal the Hbt motif as a new type of His-containing crosslinking in RiPPs. Although the Lan/Lab rings in lanthipeptides are generated in diverse stereo- and regioselectivity^59,60^, known lanthipeptide synthetases display strict chemoselectivity by utilizing Cys as the nucleophile to initiate the Michael addition with Dha/Dhb residues, regardless of the distinct Zn-dependence of their cyclase domains^8^. The discovery of the Hbt crosslink and LanKC_Hbt_ enzymes expands the structural diversity of lanthipeptides and catalytic promiscuity of lanthipeptide synthetases.

The nucleophilic addition of His(Nɛ) to the Dhb(Cβ) is chemically challenging. No chemical methods to synthesize histidinobutyrine derivatives is available in the literature, and our synthetic attempts for the Hbt crosslink following synthetic protocols of histidinoalanine all failed (Supplementary Fig. 52). Distinct from other types of His-containing crosslinking in peptides and proteins (Fig. 1), the NorKC-catalyzed Hbt formation follows a Michael addition mechanism, which requires further investigation. The catalytic potency and promiscuity of LanKC_Hbt_ enzymes to generate both Lab and Hbt motifs might originate from their cyclase domains, which are distinct from typical LanKC enzymes. Overall, this study provides a unique macrocyclic peptide scaffold and biosynthetic enzymes that hold potential in the generation of cyclic peptides with structural diversity.

## Methods

### General methods

Polymerase chain reactions (PCR) were conducted using a Bio-Rad C1000 Touch™ thermal cycler. Tsingke Biotech conducted DNA sequencing with appropriate primers. Bruker UltraFlextreme was utilized for matrix-assisted laser desorption/ionization time-of-flight mass spectrometry (MALDI-TOF MS). Liquid chromatography electrospray ionization tandem mass spectrometry (LC/ESI–MS/MS) was performed and processed using an AB Sciex Triple TOF 4600 System equipped with a Shimadzu Prominence Ultra-Fast Liquid Chromatography system. The following conditions were used for all ESI-MS and MS/MS: nebulizer gas at 55 psi, heater gas at 55 psi, curtain gas at 35 psi, drying temperature at 550 °C, ion spray voltage at 5500 V, declustering potential at 100 V, collision energy at 35 V (positive), and collision energy spread at 10 V. The mass range and accumulation time for ESI-MS were 400–4000 m/z and 250 ms, respectively, while the mass range and accumulation time for MS/MS were 100–2000 m/z and 100 ms, respectively. Collision-induced dissociation was used for fragmentation of the respective peptide ions. AB SCIEX calibration solutions were utilized for instrument calibration and high resolution was selected in ESI+ mode. NMR experiments were performed at 298 K on Bruker AVANCE III 600 MHz and AVANCE NEO 800 MHz spectrometers equipped with 5 mm z-gradient ^1^H/^13^C/^15^N TCI cryogenic probes. Two-dimensional (2D) ^1^H-^1^H DQF-COSY, TOCSY, ^1^H-^13^C/^15^N HSQC, and ^1^H-^13^C HMBC were measured to obtain chemical shifts. 2D ^1^H-^1^H NOESY experiments with 500 ms of mixing time were performed to obtain ^1^H-^1^H distance constraints. All NMR spectra were processed using TopSpin 4.1.1 and analyzed using NMRFAM-SPARKY. The chemical shifts for ^1^H were referenced to DSS, and ^13^C/^15^N chemical shifts were referenced indirectly. The Xplor-NIH program (version 3.5) was used for the structure determination and refinement. The 20 lowest energy structures were selected from 100 calculated structures for analysis. The Ramachandran statistics by PROCHECK showed 64.1% of residues within the most favored region and 26.9% of residues within the allowed region for noursin, and 54.1% of residues within the most favored region and 37.3% of residues within the allowed region for noursin_H11W_. Figure generation was performed using PyMOL (version 2.5.0). The protein SSN was generated using the EFI-EST tool.

### Materials

All oligonucleotides were purchased from Genscript Biotech (Nanjing, China). Restriction endonucleases were purchased from New England Biolabs (Ipswich, MA, USA). ClonExpress II/MultiS one Step Cloning Kits and Phanta® Max Master Mix were purchased from Vazyme Biotech (Nanjing, China). Medium components for bacterial cultures were purchased from Thermo Fisher (Waltham, MA, USA). Chemicals were purchased from Aladdin Reagent (Shanghai, China) or Sigma-Aldrich (Schnelldorf, Germany). Endoprotease GluC was purchased from Roche Biosciences (Basel, Switzerland). *E. coli* DH5α was used as the host for cloning and plasmid propagation, and *E. coli* BL21 (DE3) was used as a host for expression of proteins and peptides. *Streptomyces noursei* ATCC-11455 was purchased from the China General Microbiological Culture Collection Center (CGMCC).

### General nomenclature

The nomenclature, including the numbering of residues in precursor peptides, follows the recommendation in the review article of *Nat. Prod. Rep*. **2021**, 38, 130.

### Construction of the pNOR and pNOR-H11W cosmids

Two 6-kb PCR products with 20 bp homologous sequences were amplified from the genome of *S. noursei* ATCC 11455 using the primer pairs Nor-L-F/ Nor-L-R and Nor-R-F/ Nor-R-R (pNOR_H11W_: Nor-W-L-F/ Nor-W-L-R and Nor-W-R-F/ Nor-W-R-R), respectively. The recombinant cosmids pNOR and pNOR_H11W_ were obtained through homologous recombination among the two 6-kb PCR products and linearized plasmid pSET-152-kasO.

### Heterologous expression of the *nor* gene cluster in *S. lividans* TK24

pNOR was transferred into *S. lividans* TK24 via *E. coli* ET12567 by conjugation, and apramycin-resistant transformants were obtained. *S. lividans* TK24 harboring pNOR (*S. lividans* TK24/pNOR) was inoculated into 150 mL YEME medium and cultured under shaking conditions (220 r.p.m.) at 28 °C for 6 days. To monitor the production of peptide natural products, 2 mL of the broth was centrifuged at selected time points, and the collected mycelia was soaked in 1 mL of methanol for 10 min. After centrifugation to remove the insoluble residue, the organic and aqueous phases were evaporated to dryness separately. The resulting products were dissolved in 0.5 mL methanol, and 2 μL aliquots of the samples were used for LC-HRMS analysis.

### Culture and fermentation

*S. lividans* TK24/pNOR and *S. lividans* TK24/pNOR_H11W_ were spread separately on MS agar plates that contained a medium composed of mannitol (20 g/L), soybean meal (20 g/L), and agar (20 g/L), pH 7.0, and then incubated at 28 °C. Upon sporulation, ~1 cm^2^ of the agar was cut, chopped, transferred to 150 mL of the YEME medium, which was composed of yeast extract (4 g/L), malt extract (4 g/L), and glucose (4 g/L), pH 7.0, and then cultured under shaking conditions (220 r.p.m.) at 28 °C for 6 days.

### Isolation of noursin and noursin_H11W_

The fermentation broth of *S. lividans* TK24/pNOR or *S. lividans* TK24/pNOR_H11W_ (30 L) was centrifuged, and the pelleted mycelial cake was extracted with methanol (2 L) three times. After the mycelium was removed by filtration, the methanol extract was concentrated under reduced pressure. The aqueous fermentation medium was treated with XAD16 nonionic macroporous resin (120 g) three times. The combined resins were then soaked in 500 mL methanol, and the methanol extract was concentrated under reduced pressure. Crude mixtures obtained from the mycelium and the aqueous fermentation medium were loaded separately onto reverse-phase silica gel columns, which were eluted with H_2_O–methanol solvents. Fractions containing noursin or noursin_H11W_ were combined, concentrated and purified by RP-HPLC using an Ultimate Polar RP column (250 × 10 mm, 5 µm, Welch Technology Co., Ltd., Shanghai) by gradient elution of solvent A (H_2_O + 0.1% formic acid) and solvent B (acetonitrile + 0.1% formic acid) with a flow rate of 4 mL/min over a 35 min period as follows: *T* = 0 min, 25% solvent B; *T* = 5 min, 25% solvent B; *T* = 25 min, 50% solvent B; *T* = 27 min, 98% solvent B; *T* = 33 min, 98% solvent B and *T* = 35 min, 25% solvent B. Fractions containing noursin or noursin_H11W_ were collected, and the solvents were removed in *vacuo*. Typical yields of noursin and noursin_H11W_ are 10 mg and 20 mg from 30 L culture broth, respectively.

### The CAS-Cu^2+^ complex titrated by noursin and noursin_H11W_

A stock solution containing chrome azurol S (CAS) (500 μM), hexadecyltrimethylammonium bromide (HDTMA) (1 mM) and CuSO_4_ (80 μM) was prepared in 200 mM HEPES buffer, pH 7.5. Samples of noursin or noursin_H11W_ dissolved in water were mixed with the CAS-Cu stock solution to yield an assay solution of 20 mM HEPES buffer, 50 μM CAS, 100 μM HDTMA, 8 μM CuSO_4_ and 0–200 μM noursin or noursin_H11W_. After incubation at room temperature for 10 min, the UV–Vis spectra of these mixtures were recorded by NanoDrop 2000c (Thermo Scientific).

### Molecular cloning of the *norA* and nor*KC* genes

Plasmids containing the target genes were cloned from the genomic DNA of *S. noursei* ATCC 11455 and amplified by PCR following 30 cycles of denaturing (95 °C for 30 s), annealing (65 °C for 30 s), and extending (72 °C, 1 min/kb) using high fidelity Phanta® DNA Polymerase. Amplification of target genes was confirmed by 1% agarose gel electrophoresis. The PCR products were purified using an Omega Biotech Gel Extraction Kit. pRSFDuet-1/pACYCDuet-1 vectors were digested in separate reactions containing 1× NEB buffer (New England Biolabs) with a selected pair of restriction enzymes for 3 h at 37 °C. The digested products were purified by agarose gel electrophoresis, and the target DNA fragments were extracted using an Omega Biotech Gel Extraction Kit. The resulting DNA products were ligated by homologous recombination at 37 °C for 0.5 h in 5 × CE II buffer with CE II enzyme. *E. coli* DH5α cells were transformed with 10 μL of the ligation product by heat shock. The resulting cells were plated on LB-kanamycin/chloramphenicol agar plates and grown for 15 h at 37 °C. Single colonies were picked and used to inoculate separate 5 mL cultures of LB-kanamycin/chloramphenicol medium. The cultures were grown at 37 °C for 12 h, and plasmids were isolated using an Omega Biotech Plasmid Mini Kit. The sequences of the resulting plasmids were confirmed by DNA sequencing.

### Overexpression and purification of the His_6_-NorKC enzyme

*E. coli* BL21 (DE3) cells were transformed with the pET-28a plasmid containing the gene encoding the NorKC enzyme and the pGRO7 chaperone plasmid. A single colony was used to inoculate a 30 mL culture of LB supplemented with 50 μg/mL kanamycin and 35 mg/L of chloramphenicol. The culture was grown at 37 °C for 12 h and further used to inoculate three 1 L of LB cultures in 2 L flasks supplemented with 50 μg/mL kanamycin and 35 mg/L of chloramphenicol. The culture was grown at 37 °C to OD_600_ ~ 0.6–0.8, and cooled at 4 °C on ice for 20 min before the addition of IPTG to a final concentration of 0.2 mM. The culture was grown at 18 °C for additional 20 h. Cells were harvested by centrifugation at 12,000 × *g* for 15 min at 4 °C, and the pellet was resuspended in 30 mL of start buffer (20 mM Tris buffer, pH 8.0, 500 mM NaCl, 1.0 mM TCEP, 10% glycerol).

All protein purification steps were performed at 4 °C. The cell paste was suspended in start buffer, and the cells were lysed using a high-pressure homogenizer (Avestin, Inc.). Cell debris was removed via centrifugation at 23,700 × *g* for 20 min at 4 °C. The supernatant was loaded onto a 5 mL HisTrap HP IMAC column charged with NiSO_4_ and equilibrated with start buffer. The column was washed with 50 mL of buffer A (30 mM imidazole, 20 mM Tris, pH 7.5, 300 mM NaCl), and the protein was eluted using a linear gradient of 0–100% buffer B (200 mM imidazole, 20 mM Tris, pH 7.5, 300 mM NaCl) over 40 min at a 2 mL/min flow rate. Fractions containing proteins were collected and analyzed by SDS-PAGE. Fractions containing target proteins were combined and concentrated using an Amicon Ultra-50 Centrifugal Filter Unit (30 kDa MWCO, Millipore). The protein sample was purified by gel filtration using an FPLC system (ÄKTA) equipped with an XK16 16/60 (GE Healthcare Life Sciences) column packed with SuperDex 75 resin. Fractions containing target proteins were collected, combined and concentrated using an Amicon Ultra-15 Centrifugal Filter Unit. The resulting protein sample was stored at −80 °C. Protein concentration was determined using a Bradford Assay Kit (Pierce).

### In vitro modification of NorA by NorKC

Typically, NorA (50 µM) and His_6_-NorKC (10 µM) were incubated with 5 mM NTP and 5 mM MgCl_2_ in 20 mM Tris-HCl, pH 8.0, at 28 °C for 1 h before heated at 50 °C to quench the reaction. The supernatant of the reaction mixture was desalted by a SPE column before analysis by MALDI-TOF-MS and LC-MS/MS.

### Modification of free cysteine residues in peptides with IAA

Peptides (10 µM) were typically modified by 10 mM IAA in 20 mM Tris-HCl (pH 8.5) and 0.1 mM TCEP at room temperature in the dark for 0.5 h. The reaction was then quenched by the addition of 20 mM DTT. The reaction mixture was further analyzed by MALDI-TOF-MS.

### Modification of Dha/Dhb residues in peptides with βME

Peptides (10 µM) were typically treated by βME (10 mM) in 20 mM Tris-HCl (pH 8.0) at 37 °C for 1 h. The resulting mixture was further analyzed by MALDI-TOF-MS.

### Deprotection of NorA_His(Dmbz)-Lab_ and NorA_Cys15(oNBn)-hydrated_

NorA_His(Dmbz)-Lab_ (50 µM) was dissolved in 500 µL of the ammonolytic reagent (28% NH_3_(aq): EtOH = 3:1) and incubated at room temperature for 1 h. NorA_Cys15(oNBn)-hydrated_ (50 µM) was subjected to UV (360 nm) irradiation by a portable UV lamp source (5 W) for 5 min.

### Marfey assay for chiral analysis of native amino acid residues in noursin

Noursin (2 mg) was hydrolyzed in 2 mL 6 N HCl in a sealed tube at 110 °C for 24 h. The hydrolysate was dried under reduced pressure and dissolved in 20 μL ddH_2_O, transferred to a 1.5 mL Eppendorf tube, which was followed by the addition of 40 μL of 1% acetone solution of FDAA (N-(5-fluoro-2, 4-dinitrophenyl)-L-alaninamide, Marfey’s reagent), and 8 μL of 1 N NaHCO_3_ solution for derivatization. The reaction mixture was heated with frequent shaking over a hot plate at 40 °C for 1 h and cooled to ambient temperature. The reaction was quenched by the addition of 4 μL of 2 N HCl and diluted with 0.2 mL MeOH. Standards (D/L-amino acid) were treated identically. FDAA-derivatized amino acids (2 μL) were injected onto an Agilent G6530 HRESI-QTOF mass spectrometer equipped with an Agilent 1260 HPLC system and a C18 reverse-phase column (Agilent Zorbax SB-C18, 3.5 μm, 2.1 × 150 mm). The elution gradient was: 0–20 min, 10–80% B; 20–25 min, 80–100% B; 26–35 min, 10% B; using H_2_O (0.1% FA, A), and ACN (0.1% FA, B) as mobile phases at a flow rate of 0.2 mL/min. The mass spectrometer was operated in positive mode with a mass range of 50–1700 *m/z*. The molecular mass corresponding to FDAA-residues was extracted for data analysis.

### Hydrolysis, derivatization and LC-MS analysis of modified peptides

Total hydrolysis of products (1 mg) was performed at 110 °C in an aqueous 6 M hydrochloric acid solution (1 mL) under vacuum for 12 h in glass-ampoules. After 12 h, the mixtures were dried under vacuum. 500 µL of 2 M ethanolic HCl-solution, generated from acetylchloride in abs. ethanol (1:4, v:v) in a Reacti-Vial was added to the dry hydrolysate. Samples were heated for 30 min at 110 °C and reagents were removed under vacuum. Acetylation was performed by adding 200 µL dichloromethane and 100 µL trifluoroacetic anhydride to the samples. The mixtures were heated again for 10 min at 110 °C. Excess of reagents was removed under vacuum. The resulting residues were dissolved in 50 µL MeOH and analyzed by LC-MS.

### Desulfurization of modified peptides

NaBH_4_ (1.0 mg) was added to a suspension of modified peptides (0.5 mg) and NiCl_2_ (2.0 mg) in MeOH (500 µL) and H_2_O (500 µL) in a Reacti-Vial, which was then immediately sealed. The reaction was heated to 50 °C for 4 h. The reaction mixture was concentrated and freeze-dried. After desalting, the desulfurized product was eluted with MeCN and freeze-dried.

### Reporting summary

Further information on research design is available in the Nature Portfolio Reporting Summary linked to this article.

## Supplementary information

Supplementary Information

Peer Review File

Reporting Summary

## Data Availability

The NMR structures of noursin and noursin_H11W_ generated in this study have been deposited in the PDB database under accession numbers 7YFS and 8HZW, respectively. The accession numbers of NorKC, AblKC and TamKC proteins from NCBI database are ANZ21440.1, WP_189866512.1, and WP_184696628.1, respectively. Supplementary information is available for this paper online. Correspondence and requests for materials should be addressed to H.W., H.Y., or J.G.
