# Supplementary material for: Discovery and biosynthesis of tricyclic copper-binding ribosomal peptides containing histidine-to-butyrine crosslinks
Source: Nat Commun. 2023 May 23;14:2944. doi: 10.1038/s41467-023-38517-2 (PMC10206099; doi:10.1038/s41467-023-38517-2)
Supplement: Supplementary file 1 — Supplementary Information [file 41467_2023_38517_MOESM1_ESM.pdf]

Supplementary Information

Discovery and biosynthesis of tricyclic copper-binding ribosomal peptides containing histidine-to-butyrine crosslinks

Yuqing Li,[1] Yeying Ma,[1] Yinzheng Xia,[1] Tao Zhang,[2] Shuaishuai Sun,[1] Jiangtao Gao,[2*] Hongwei Yao[3*] and Huan Wang[1*]

[1]State Key Laboratory of Coordination Chemistry, Chemistry and Biomedicine Innovation Center of Nanjing University, Jiangsu Key Laboratory of Advanced Organic Materials, School of Chemistry and Chemical Engineering, Nanjing University, Nanjing, 210093, China

[2]State Key Laboratory of Ecological Pest Control for Fujian and Taiwan Crops, College of Life Sciences, Fujian Agriculture and Forestry University, 350002, Fuzhou, China

[3]Institute of Molecular Enzymology, School of Biology and Basic Medical Sciences, Soochow University, Suzhou, 215123, P.R. China

*e-mail: wanghuan@nju.edu.cn; hwyao@suda.edu.cn; jgaotao@gmail.com

List of supplementary figures and tables:

• Supplementary Table 1 | Predicted functions of proteins encoded in the *nor* BGC
• Supplementary Table 2 | Chemical shifts of noursin in CD_3OH
• Supplementary Table 3 |Assigned correlations of 2D ^{1}H-^{1}H COSY, ^{1}H-^{13}C HMBC, and ^{1}H-^{1}H NOESY spectra of noursin.
• Supplementary Table 4 | Chemical shifts of noursin$_{H11W}$ in CD_3OD/H_2O
• Supplementary Table 5 | NMR and refinement statistics for noursin and noursinH11W structures
• Supplementary Table 6 | The energy and violation statistics for the 20 lowest energy unrefined structures of noursin for Hbt8Cα/Cβ at *R*/*S*, *R*/*R*, *S*/*R*, and *S*/*R* configurations, respectively
• Supplementary Table 7 | The dihedral angle values of each amino acid residue in noursin
• Supplementary Table 8 | Primers used in this study
• Supplementary Figure 1 | Heterologous expression of the *nor* BGC and MS analysis of noursin
• Supplementary Figure 2 | HPLC analysis of the purified noursin sample
• Supplementary Figure 3 | Marfey's analysis of noursin
• Supplementary Figure 4 | MS and MS/MS analysis of noursin
• Supplementary Figure 5 | 2D ^{1}H-^{1}H DQF-COSY spectrum of noursin in CD_3OH at 298 K
• Supplementary Figure 6 | 2D ^{1}H-^{1}H TOCSY spectrum of noursin in CD_3OH at 298 K with 30 ms of mixing time
• Supplementary Figure 7 | 2D ^{1}H-^{1}H NOESY spectrum of noursin in CD_3OH at 298 K with 500 ms of mixing time
• Supplementary Figure 8 | 2D ^{1}H-^{13}C HSQC spectrum of noursin in CD_3OH at 298 K
• Supplementary Figure 9 | 2D ^{1}H-^{13}C HMBC spectra of noursin in CD_3OH at 298 K
• Supplementary Figure 10 | 2D ^{1}H-^{15}N HSQC spectra of noursin in CD_3OH at 298 K
• Supplementary Figure 11 | The LC-MS analysis of the Lab derivatives of noursin and LabA2$_{modified}$
• Supplementary Figure 12 | The 2D ^{1}H-^{13}C HMBC spectrum clearly showed the Hbt crosslink
• Supplementary Figure 13 | Heterologous expression and isolation of noursin$_{H11W}$
• Supplementary Figure 14 | ^{1}H NMR spectra of noursin$_{H11W}$ in CD_3OD/H_2O (70%/30%, pH ~5.0)
• Supplementary Figure 15 | 2D ^{1}H-^{1}H TOCSY spectrum of noursin$_{H11W}$ in CD_3OD/H_2O (70%/30%, pH ~5.0) at 298 K with 80 ms of mixing time
• Supplementary Figure 16 | 2D ^{1}H-^{1}H NOESY spectrum of noursin$_{H11W}$ in CD_3OD/H_2O (70%/30%, pH ~5.0) at 298 K with 500 ms of mixing time
• Supplementary Figure 17 | 2D ^{1}H-^{13}C HSQC spectrum of noursin$_{H11W}$ in CD_3OD/H_2O (70%/30%, pH ~5.0) at 298 K
• Supplementary Figure 18 | 2D ^{1}H-^{13}C HMBC spectrum of noursin$_{H11W}$ in CD_3OD/H_2O (70%/30%, pH ~5.0) at 298 K
• Supplementary Figure 19 | LC-MS analysis of the Lab and the Hbt crosslinks in noursin and NorA$_{modified}$
• Supplementary Figure 20 | The assignment of Cβ of Hbt8 in the *S* configuration
• Supplementary Figure 21 | A cross-eye stereo of the superimposed 20 lowest energy structures of noursin
• Supplementary Figure 22 | A cross-eye stereo of the superimposed 20 lowest energy structures of noursin$_{H11W}$
• Supplementary Figure 23 | The structure of ring A (a) and C-terminal residues (b) in the lowest energy conformer of noursin and the hydrogen bonding network

• Supplementary Figure 24 | The circular dichroism spectra of noursin in H_2O at 298 K

• Supplementary Figure 25 | The metal binding ability of noursin and its derivatives analyzed by MALDI-TOF-MS

• Supplementary Figure 26 | The electro paramagnetic resonance (EPR) spectra of the mixed samples of noursin with CuSO4 at 298 K

• Supplementary Figure 27 | Heterologous expression of the *nor* BGCs with gene deletions

• Supplementary Figure 28 | Requirement of NTP during the modification of NorA by NorKC

• Supplementary Figure 29 | Tandem MS analysis of NorA$_{\text{modified}}$ generated *in vitro*

• Supplementary Figure 30 | The formation of the Lab motif catalyzed by NorKC is independent of the formation of the Hbt motif

• Supplementary Figure 31 | NorA$_{\text{Lab}}$ was partially linearized through desulfurization and the LC-MS/MS analysis of linearized NorA$_{\text{Lab}}$

• Supplementary Figure 32 | LC-MS analysis of the Lab and the Hbt derivatives from NorA$_{\text{Lab}}$ and NorA$_{\text{modified}}$

• Supplementary Figure 33 | Sequential modification of NorA$_{\text{Cys15(oNBn)}}$ by NorKC

• Supplementary Figure 34 | Modification of NorA$_{\text{CP}}$ by NorKC is incomplete and inefficient

• Supplementary Figure 35 | The formation of the Hbt crosslink by NorKC requires the leader peptide

• Supplementary Figure 36 | Histidine does not reactive with a Dhb derivative or Dhb8 in NorA$_{\text{H11A_modified}}$

• Supplementary Figure 37 | The formation of a Lab ring in NorA increases the reactivity of Dhb8

• Supplementary Figure 38 | The C’-Cα-Cβ bond angle of Hbt8 residue and Dhb8 in NMR structures of noursin and noursin$_{\text{H11W}}$

• Supplementary Figure 39 | Modification of NorA$_{\text{C15H}}$ by NorKC

• Supplementary Figure 40 | Modification of NorA$_{\text{D-H11}}$ by NorKC

• Supplementary Figure 41 | Modification of NorA$_{\text{H11K}}$ by NorKC

• Supplementary Figure 42 | Modification of NorA$_{\text{T8A}}$ by NorKC

• Supplementary Figure 43 | Modification of NorA$_{\text{T8S}}$ by NorKC

• Supplementary Figure 44 | Modification of NorA$_{\text{S3A}}$ by NorKC

• Supplementary Figure 45 | Modification of NorA$_{\text{S7A}}$ by NorKC

• Supplementary Figure 46 | NorKC catalyzes the formation of the Lab and the Hbt motif in the NorA$_{\text{L9F}}$ peptide

• Supplementary Figure 47 | NorKC catalyzes the formation of the Lan and the Hbt motif in the NorA$_{\text{insert8A}}$ peptide

• Supplementary Figure 48 | BGCs homologous to the *nor* BGC found in bacterial genomes

• Supplementary Figure 49 | AblKC catalyzes the formation of the Lab and the Hbt motif in the AblA peptide

• Supplementary Figure 50 | TamKC catalyzes the formation of the Lab and the Hbt motif in the TamA peptide

• Supplementary Figure 51 | The colored SSN for the cyclase domains, kinase domains, and lyase domains of class III lanthipeptide synthases

• Supplementary Figure 52 | Synthetic attempts to synthesize Hbt crosslinks.

Supplementary Table 1. Predicted functions of proteins encoded in the *nor* BGC.

Protein	Length (AA)	Accession number	Putative function
orf 1	173	ANZ21439.1 https://www.ncbi.nlm.nih.gov/protein/1051502734	Response regulator
NorA	34	ANZ21441.1 https://www.ncbi.nlm.nih.gov/protein/1051502736	Precursor peptide
NorKC	878	ANZ21440.1 https://www.ncbi.nlm.nih.gov/protein/ANZ21440.1	Class III lanthipeptide synthetase
NorP	690	ANZ21442.1 https://www.ncbi.nlm.nih.gov/protein/1051502737	S9 family peptidase
NorB	289	ANZ21443.1 https://www.ncbi.nlm.nih.gov/protein/1051502738	6-chlorohydroxyquinol-1,2-dioxygenase
NorC	468	ANZ21444.1 https://www.ncbi.nlm.nih.gov/protein/1051502739	Xylulokinase
NorD	333	ANZ21445.1 https://www.ncbi.nlm.nih.gov/protein/1051502740	Zinc-dependent dehydrogenase
NorE	246	ANZ21446.1 https://www.ncbi.nlm.nih.gov/protein/1051502741	DeoR/GlpR transcriptional regulator
NorF	419	ANZ21447.1 https://www.ncbi.nlm.nih.gov/protein/1051502742	MFS transporter
NorG	436	ANZ21448.1 https://www.ncbi.nlm.nih.gov/protein/1051502743	Erythromycin esterase family protein

Supplementary Table 2. Chemical shifts of noursin in CD_3OH.

Residue	N	H^N	Cα	Hα	Cβ	Hβ	Other
A1			50.6	4.34	18.1	1.55	172.2(C')
P2			62.3	4.90	31.7	2.09, 2.35	175.5(C'), 50.0(Cδ), 3.69(Hδ2), 3.78(Hδ3), 27.2(Cγ), 2.20(Hγ2), 2.06(Hγ3)
Lab3	120.0	8.89	53.9	4.19	35.4	2.80, 2.66	180.1(C')
N4	118.6	9.00	56.7	4.18	38.6	2.88, 3.17	176.0(C'), 108.7(Nδ2), 7.73(Hδ21), 7.07(Hδ22)
V5	121.7	8.51	66.2	3.78	31.9	2.04	176.9(C'), 21.5(Cγ1), 1.05(Hγ1*), 21.0(Cγ2), 0.94(Hγ2*)
L6	118.0	8.38	57.3	3.96	41.8	1.69, 1.61	178.1(C'), 23.6(Cδ1), 0.89(Hδ1*), 24.1(Cδ2), 0.92(Hδ2*), 27.2(Cγ), 1.58(Hγ)
Lab7	122.8	8.81	65.4		38.4	3.55, 2.12	178.2(C')
Hbt8		8.25	61.1	4.27	56.6	5.53	170.3(C'), 17.6(Cγ), 1.69(Hγ*)
L9	116.1	7.58	56.3	4.50	43.5	1.81, 1.63	177.4(C'), 22.5(Cδ1), 0.90(Hδ1*), 25.2(Cδ2), 0.95(Hδ2*), 27.2(Cγ), 1.78(Hγ)
L10	115.2	7.30	53.6	4.75	45.9	1.49, 1.86	176.3(C'), 23.0(Cδ1), 0.90(Hδ1*),

							25.8(Cδ2), 0.84(Hδ2*), 26.6(Cγ), 1.50(Hγ)
Hbt11	120.3	7.18	57.9	4.22	28.5	2.98, 3.29	175.9(C'), 126.3(Cδ2), 7.77(Hδ2), 135.0(Cε1), 8.26(Hε1), 131.8(Cγ)
G12	110.6	9.03	45.5	3.76, 4.38			172.4(C')
R13	116.6	8.33	56.8	4.65	31.7	1.72, 1.86	174.6(C'), 43.3(Cδ), 3.18(Hδ*), 27.4(Cγ), 1.65(Hγ2), 1.60(Hγ3), 83.4(Nε), 7.32(Hε)
A14	115.3	7.64	51.4	4.62	21.1	1.41	174.2(C')
Lab15	111.1	7.55	53.5	4.86	40.5	3.15, 2.83	172.5(C')
V16	124.0	8.27	66.3	3.93	32.1	1.88	181.3(C'), 21.2(Cγ1), 1.06(Hγ1*), 21.5(Cγ2), 0.98(Hγ2*)

Supplementary Table 3. Assigned correlations of 2D ^{1}H-^{1}H COSY, ^{1}H-^{13}C HMBC, and ^{1}H-^{1}H NOESY spectra of noursin. Key correlations of the Lab and the Hbt crosslinks are highlighted in bold.

**^{1}H-^{1}H COSY**	**^{1}H-^{13}C HMBC**		**^{1}H-^{1}H NOESY**		
A1HB-HA	A1C'-HA	L9CD2-HB2	A1HA-HB	**Lab7HB2-Lab15HB2**	Hbt11HA-HD2
P2HB2-HB3	A1C'-HB	L9CD2-HB3	A1HB-P2HD2	**Lab7HB2-Lab15HB3**	Hbt11HA-G12HN
P2HG2-HD2	A1CA-HB	L9CD2-HD1*	A1HB-P2HD3	**Lab7HB3-Lab3HA**	Hbt11HA-R13HN
P2HG2-HD3	A1CB-HA	L9CG-HA	P2HA-HB2	**Lab7HB3-Lab3HB2**	Hbt11HA-A14HN
P2HG3-HD2	P2C'-HB2	L9CG-HD2*	P2HA-HB3	**Lab7HB3-Lab3HB3**	Hbt11HA-Lab15HN
P2HG3-HG2	P2C'-HB3	L10C'-HA	P2HA-V16HB	Lab7HB3-HN	Hbt11HB2-L10HN
**Lab3HA-HN**	P2C'-Lab3HN	L10C'-HB2	P2HB2-Lab3HN	Lab7HB3-HB2	Hbt11HB2-HN
**Lab3HB3-HA**	P2CA-HB3	L10C'-HB3	P2HB2-N4HN	Lab7HB3-L10HN	Hbt11HB2-HD2
**Lab3HB3-HB2**	P2CA-HD2	L10C'-Hbt11H	P2HB3-HB2	**Lab7HB3-Lab15HB2**	Hbt11HB2-G12HN
N4HA-HN	P2CA-HD3	L10CA-HN	P2HB3-HG2	**Lab7HB3-Lab15HB3**	Hbt11HB3-HN
N4HB2-HA	P2CA-HG2	L10CA-HB2	P2HB3-HG3	Hbt8HN-HA	Hbt11HB3-HA
N4HB2-HB3	P2CA-HG3	L10CA-HB3	P2HB3-Lab3HN	Hbt8HA-HB	Hbt11HB3-HB2
N4HB3-HA	P2CB-HD2	L10CA-HD2*	P2HB3-Lab3HB3	Hbt8HA-HG*	Hbt11HB3-HD2
N4HD22-HD21	P2CB-HD3	L10CB-HA	P2HD2-A1HA	Hbt8HA-L9HN	Hbt11HB3-G12HN
V5HA-HN	P2CB-HG2	L10CB-HD1*	P2HD2-HB2	Hbt8HA-L9HB2	Hbt11HD2-HE1
V5HB-HA	P2CB-HG3	L10CB-HD2*	P2HD2-HB3	Hbt8HA-L10HN	Hbt11HE1-Lab3HN
V5HG1*-HB	P2CD-HB2	L10CB-HG	P2HD2-HG2	**Hbt8HA-Hbt11HD2**	Hbt11HE1-Lab7HN
V5HG2*-HB	P2CD-HB3	L10CD1-HB2	P2HD2-HG3	Hbt8HB-L9HN	Hbt11HE1-HA
L6HA-HN	P2CD-HG2	L10CD1-HB3	P2HD3-A1HA	**Hbt8HB-Hbt11HD2**	G12HA2-HN
L6HB2-HA	P2CD-HG3	L10CD1-HD2*	P2HD3-HB2	**Hbt8HB-Hbt11HE1**	G12HA2-R13HN
L6HB3-HA	P2CG-HB2	L10CG-HA	P2HD3-HB3	Hbt8HG*-Lab3HB3	G12HA3-HN
L6HG-HD1*	P2CG-HB3	L10CG-HB2	P2HD3-HD2	Hbt8HG*-HN	G12HA3-HA2
L6HG-HD2*	P2CG-HD2	L10CG-HB3	P2HD3-HG2	Hbt8HG*-HB	G12HA3-R13HN
**Lab7HB3-HB2**	P2CG-HD3	L10CG-HD2*	P2HD3-HG3	**Hbt8HG*-Hbt11HD2**	R13H-G12HN
**Hbt8HB-HA**	Lab3C'-HB3	Hbt11C'-HA	P2HG2-HB2	**Hbt8HG*-Hbt11HE1**	R13HA-HN
**Hbt8HG*-HB**	Lab3C'-N4HN	Hbt11C'-HB2	P2HG2-HG3	L9HN-Lab7HN	R13HA-HB2
L9HA-HN	Lab3C'-N4HA	Hbt11C'-HB3	Lab3HA-HN	L9HN-Hbt8HN	R13HA-HB3
L9HB2-HA	Lab3CA-HB3	Hbt11C'-G12HN	Lab3HA-HB2	L9HN-Hbt11HD2	R13HA-HG2
L9HB3-HB2	**Lab3CB-Lab7HB2**	Hbt11C'-G12HA2	Lab3HA-HB3	L9HA-Hbt8HA	R13HA-HG3
L9HG-HD1*	**Lab3CB-Lab7HB3**	Hbt11C'-G12HA3	**Lab3HA-Lab7HN**	L9HA-HN	R13HA-A14H
L9HG-HD2*	N4C'-HA	Hbt11CA-HN	**Lab3HA-Lab15HB2**	L9HA-HB2	R13HB2-HN
L10HA-HN	N4C'-HB2	Hbt11CA-HB2	**Lab3HA-Lab15HB3**	L9HA-HB3	R13HB2-HB3

L10HB2-HA	N4CB-HN	Hbt11CA-HD2	Lab3HB2-HN	L9HA-HG	R13HB2-A14HN
L10HB2-HB3	N4CB-HA	Hbt11CB-HN	Lab3HB2-HB3	L9HA-L10HN	R13HB3-HN
L10HD1*-HG	N4CB-HD22	Hbt11CB-HA	Lab3HB2-N4HN	L9HA-Hbt11HN	R13HB3-A14HN
L10HD2*-HG	V5C'-HB	Hbt11CB-HD2	**Lab3HB2-Lab7HN**	L9HA-Hbt11HD2	R13HB3-A14HB
**Hbt11HA-HN**	V5CA-HB	**Hbt11CD2-Hbt8HB**	Lab3HB2-Hbt11HE1	L9HB2-L6HA	R13HG2-HN
**Hbt11HB2-HA**	V5CA-HG2*	Hbt11CD2-HB2	Lab3HB3-HN	L9HB2-HN	R13HG2-A14HN
**Hbt11HB2-HB3**	V5CB-HG2*	Hbt11CD2-HB3	Lab3HB3-N4HN	L9HB2-L10HN	R13HG3-HN
**Hbt11HB3-HA**	V5CG1-HG2*	Hbt11CD2-HE1	**Lab3HB3-Lab7HN**	L9HB3-HN	R13HG3-A14HN
**Hbt11HD2-HE1**	V5CG2-HG1*	**Hbt11CE1-Hbt8HB**	Lab3HB3-Hbt11HE1	L9HB3-HB2	A14HN-G12HN
G12HA2-HN	L6C'-HB3	Hbt11CE1-HD2	N4H-Lab3HN	L9HB3-L10H	A14HN-R13HN
G12HA2-HA3	L6CB-HD1*	Hbt11CG-HA	N4HA-HN	L9HD1*-Hbt8HA	A14HN-Lab15HN
G12HA3-HN	L6CB-HD2*	Hbt11CG-HB2	N4HA-HB2	L9HD1*-HN	A14HA-HN
R13HA-HN	L6CB-HG	Hbt11CG-HB3	N4HA-HB3	L9HD1*-HA	A14HA-HB
R13HB2-HA	L6CD1-HD2*	Hbt11CG-HD2	N4HA-V5HN	L9HD2*-L6HA	A14HA-Lab15HN
R13HB2-HB3	L6CD2-HD1*	Hbt11CG-HE1	N4HB2-HN	L9HD2*-HN	A14HB-R13HN
R13HD*-HE	L6CD2-HG	G12C'-HA2	N4HB2-HD21	L10HN-L9HN	A14HB-HN
A14HA-HN	L6CG-HB3	G12C'-HA3	N4HB2-HD22	L10HN-Hbt11HD2	A14HB-Lab15HN
A14HB-HA	L6CG-HD1*	G12C'-R13HN	N4HB2-V5HN	L10HA-L9HN	A14HB-Lab15HB3
**Lab15HA-HN**	**Lab7C'-Lab3HB3**	G12C'-R13HA	N4HB3-HN	L10HA-HN	Lab15HN-G12HN
**Lab15HB3-HB2**	Lab7C'-HB2	G12CA-HN	N4HB3-HB2	L10HA-HB2	Lab15HN-R13HN
V16HA-HN	Lab7C'-HB3	R13C'-HA	N4HB3-V5HN	L10HA-HB3	Lab15HA-HN
V16HB-HA	Lab7C'-Hbt8HA	R13C'-HB2	N4HD22-HD21	L10HA-HD1*	Lab15HA-HB2
V16HG1*-HB	**Lab7CA-Lab3HB3**	R13C'-A14HN	V5HN-N4HN	L10HA-HG	Lab15HA-HB3
V16HG2*-HB	Lab7CA-HB2	R13CA-HN	V5HN-Lab7HN	L10HA-Hbt11HN	Lab15HA-V16HN
	Lab7CA-HB3	R13CB-HA	V5HA-HN	L10HA-Hbt11HD2	**Lab15HB2-Lab3HN**
	**Lab7CB-Lab3HB3**	R13CB-HD*	V5HA-L6HN	L10HA-R13HN	**Lab15HB2-Lab3HB3**
	**Lab7CB-Lab15HB3**	R13CB-HG2	V5HB-HN	L10HA-A14HN	Lab15HB2-HN
	Hbt8C'-HA	R13CB-HG3	V5HB-L6HN	L10HB2-Lab7HB2	Lab15HB2-HB3
	Hbt8C'-HB	R13CG-HA	V5HG1*-HN	L10HB2-Lab7HB3	Lab15HB2-V16HN
	Hbt8C'-HG*	R13CG-HD*	V5HG1*-HA	L10HB2-L9HN	Lab15HB3-HN
	Hbt8C'-L9HN	A14C'-HA	V5HG1*-HB	L10HB2-HN	Lab15HB3-V16HN
	Hbt8C'-L9HA	A14C'-HB	V5HG2*-HA	L10HB2-HB3	V16HA-G12HN
	Hbt8CA-HB	A14C'-Lab15HN	V5HG2*-HB	L10HB2-Hbt11HN	V16HA-Lab15HN
	Hbt8CA-HG*	A14CA-HN	V5HG2*-L6HN	L10HB3-Lab7HB2	V16HA-HN
	Hbt8CB-HA	A14CB-HA	L6HN-Lab7HN	L10HB3-HN	V16HA-HB
	Hbt8CB-HG*	Lab15C'-HN	L6HA-HN	L10HB3-Hbt11HN	V16HB-P2HB3
	**Hbt8CB-Hbt11HD2**	Lab15C'-HB3	L6HA-Lab7HN	L10HB3-Lab15HN	V16HB-Lab3HN
	Hbt8CG2-HA	Lab15C-V16HA	L6HA-L9HN	L10HD1*-HN	V16HB-HN
	Hbt8CG2-HB	Lab15CA-HN	L6HB2-HN	L10HD1*-HB3	V16HG1*-HA
	L9C'-HA	Lab15CA-HB3	L6HB2-HA	L10HD1*-A14HN	V16HG1*-HB
	L9C'-HB2	**Lab15CB-Lab7HB2**	L6HB2-Lab7HN	L10HD1*-Lab15HN	V16HG2*-HN
	L9C'-HB3	**Lab15CB-Lab7HB3**	L6HB3-HN	L10HD2*-Lab7HB2	V16HG2*-HA
	L9C'-L10H	V16C'-HA	L6HB3-HA	L10HD2*-HN	V16HG2*-HB
	L9C'-L10HA	V16C'-HB	L6HB3-Lab7HN	L10HD2*-HB3	
	L9CA-HN	V16CA-HB	L6HB3-Lab7HB3	L10HD2*-A14HN	
	L9CA-HB2	V16CA-HG1*	Lab7HN-N4HN	L10HG-A14HN	
	L9CA-HD2*	V16CA-HG2*	**Lab7HB2-Lab3HA**	L10HG-Lab15HN	
	L9CB-HN	V16CB-HA	**Lab7HB2-Lab3HB2**	Hbt11HN-L9HN	
	L9CB-HA	V16CB-HG1*	Lab7HB2-L6HA	Hbt11HN-L10HN	

	L9CB-HD1*	V16CB-HG2*	Lab7HB2-HN	Hbt11HN-HD2	
	L9CB-HD2*	V16CG1-HG2*	Lab7HB2-L9HN	Hbt11HN-G12HN	
	L9CD1-HB2	V16CG2-HA	Lab7HB2-L10HN	Hbt11HA-L10HN	
	L9CD1-HB3	V16CG2-HG1*	Lab7HB2-Hbt11HN	Hbt11HA-HN	
	L9CD1-HD2*		**Lab7HB2-Lab15HN**	Hbt11HA-HB2	

Supplementary Table 4. Chemical shifts of noursin$_{H11W}$ in CD_3OD/H_2O (70%/30%).

Residue	N	H^N	Cα	Hα	Cβ	Hβ	Other
A1		8.19	50.9	4.29	17.7	1.53	
P2			63.0	4.48	31.9	1.94, 2.33	50.1(Cδ), 3.64(Hδ2), 3.72(Hδ3), 27.6(Cγ), 2.14(Hγ2), 2.03(Hγ3)
Lab3	122.5	8.95	52.6	4.68	37.9	2.45, 2.83	
N4	121.0	8.57	56.9	4.28	37.8	2.90	111.4(Nδ2), 7.75(Hδ21), 7.09(Hδ22)
V5	119.7	8.32	63.6	3.97	31.5	2.09	21.0(Cγ1), 0.90(Hγ1*), 20.9(Cγ2), 0.95(Hγ2*)
L6	118.5	8.62	56.1	3.99	40.7	1.88, 1.65	25.2(Cδ*), 0.91(Hδ*), 27.0(Cγ), 1.62(Hγ)
Lab7	124.7	8.68			38.7	3.79, 2.66	
Dhb8		9.52	131.8		135.9	6.67	168.9(C'), 14.9(Cγ), 1.74(Hγ*)
L9	117.5	7.67	56.5	4.34	42.7	1.76, 1.64	25.2(Cδ1), 0.94(Hδ1*), 23.0(Cδ2), 0.88(Hδ2*), 27.2(Cγ), 1.71(Hγ)
L10		7.61		4.36	42.0	1.70	0.94(Hδ1*), 0.88(Hδ2*), 27.2(Cγ), 1.64(Hγ)
W11		7.81	59.2	4.27	28.1	3.34	128.1(Nε1), 10.20(Hε1), 126.4(Cδ1), 7.16(Hδ1), 130.0(Cδ2), 139.3(Cε2), 120.6(Cε3), 7.49(Hε3), 112.1(Cγ), 124.1(Cη2), 7.13(Hη2), 114.1(Cξ2), 7.40(Hξ2), 121.3(Cξ3), 7.03(Hξ3)
G12	109.3	8.10	45.6		4.01, 3.61		
R13	118.1	7.97	56.4	4.34	30.8	1.84, 1.96	43.5(Cδ), 3.22(Hδ*), 27.6(Cγ), 1.70(Hγ*), 84.0(Nε), 7.37(Hε)
A14	120.0	8.10	52.1	4.40	19.1	1.39	
Lab15		7.84	56.4	4.55	39.2	3.03, 2.97	
V16	118.4	8.34	60.8	4.23	33.1	2.17	21.0(Cγ1), 0.93(Hγ1*), 19.8(Cγ2), 0.93(Hγ2*)

Supplementary Table 5. NMR and refinement statistics for noursin and noursin$_{H11W}$ structures.

	**noursin**	**noursin$_{H11W}$**
**NMR distance and dihedral constraints**		
Distance constraints		
Total NOE	245	257
Intra-residue	92	78
Inter-residue		
Sequential ($\|i-j\| = 1$)	58	45
Medium-range ($\|i-j\| < 4$)	40	34
Long-range ($\|i-j\| > 5$)	17	17
**Structure statistics**		
Violations (mean and s.d.)		
Distance constraints (Å)	0.016±0.003	0.022±0.003
Max. distance constraint violation (Å)	0.293	0.232
Deviations from idealized geometry		
Bond lengths (Å)	0.003±0.000	0.003±0.000
Bond angles (º)	0.559±0.008	0.538±0.009
Impropers (º)	0.391±0.030	0.329±0.015
Average pairwise r.m.s. deviation** (Å)		
Heavy	0.63±0.12	0.75±0.13
Backbone	0.15±0.05	0.30±0.08

Pairwise r.m.s. deviation was calculated among 20 refined structures.

Supplementary Table 6. The energy and violation statistics for the 20 lowest energy unrefined structures of noursin for Hbt8Cα/Cβ at *R*/*S*, *R*/*R*, *S*/*R*, and *S*/*R* configurations, respectively.

Parameter	Hbt8Cα^{R}Cβ^S	Hbt8Cα^{R}Cβ^R	Hbt8Cα^{S}Cβ^R	Hbt8Cα^{S}Cβ^S
Distance restraints	247	247	247	247
Average Energy (kcal)				
torsionDB potential	405.14±7.76	405.02±11.16	474.75±20.61	485.72±21.68
NOE distance	20.73±2.35	48.51±2.53	392.31±59.77	378.23±107.92
van der Waals repel	9.88±1.09	11.03±0.99	87.10±16.70	84.98±21.18
bond angles	30.14±1.83	31.53±1.83	199.93±14.77	198.38±12.93
bond lengths	3.86±0.22	7.21±0.22	33.76±5.49	33.77±7.58
improper dihedral angles	5.03±0.34	4.72±0.30	44.06±9.00	42.94±5.79
Total	474.78±8.75	508.02±10.32	1231.91±39.89	1224.02±152.08
Maximum NOE violation (Å)	0.419	0.946	2.037	2.021

Supplementary Table 7. The dihedral angle values of each amino acid residue in noursin. Unusual dihedral angle values are highlighted with *.

**Position**	***ϕ***	***ψ***	**Position**	***ϕ***	***ψ***
Ala1		74.8	Leu9	-106.2	-10.0*
Pro2	-71.3	152.0	Leu10	-121.8	40.4
Lab3	-61.2	-41.4	Hbt11	-72.0	140.6
Asn4	63.1*	-85.6*	Gly12	83.2	1.1
Val5	-107.2	-44.2	Arg13	-13.5*	-79.7
Leu6	-91.4	-13.6*	Ala14	-90.7	-9.6*
Lab7	-59.9	-43.9	Lab15	-155.5	167.2
Hbt8	-44.9*	-30.9	Val16	-106.1	

Supplementary Table 8. Primers used in this study.

Primer	Sequence (5'-3')
Nor-L-F	GCTGCATGCATACGTACTAGTGTGCGGGCCCGCTGTTCT
Nor-L-R	TCGATGCTGGTCGAGACCATCAA
Nor-R-F	ATGGTCTCGACCAGCATCGACACCCCGAACACGTCGG
Nor-R-R	CTATGACATGATTACGAATTCGATCATCGCCGCCGGCTC
Nor-W-L-F	GCTGCATGCATACGTACTAGTGTGCGGGCCCGCTGTTCT
Nor-W-L-R	AGAAGCGTGCTCAGCACGTTGCTCGG
Nor-W-R-F	AACGTGCTGAGCACGCTTCTCTGGGGCCGC
NorW-R-R	CTATGACATGATTACGAATTCGATCATCGCCGCCGGCTC
pRSF-NorA-F	TCATCACCACAGCCAGGATCCGATGGCCATGATCCTGGAGCTTCAG
pRSF-NorA-R	GCATTATGCGGCCGCAAGCTTTCACACACAGGCGCGGCC
pACYC-NorKC-F	TAATAAGGAGATATACCATGGAGCTGGCGGCGTTC
pACYC-NorKC-R	GCATTATGCGGCCGCAAGCTTTCACCTCCTCTCTTTTCTTTTGGACGT
pET-28a-NorKC-F	GTGCCGCGCGGCAGCCATATGGAGCTGGCGGCGTTC
pET-28a-NorKC-R	GTGGTGGTGGTGGTGCTCGAGTCACCTCCTCTCTTTTCTTTTGGACG

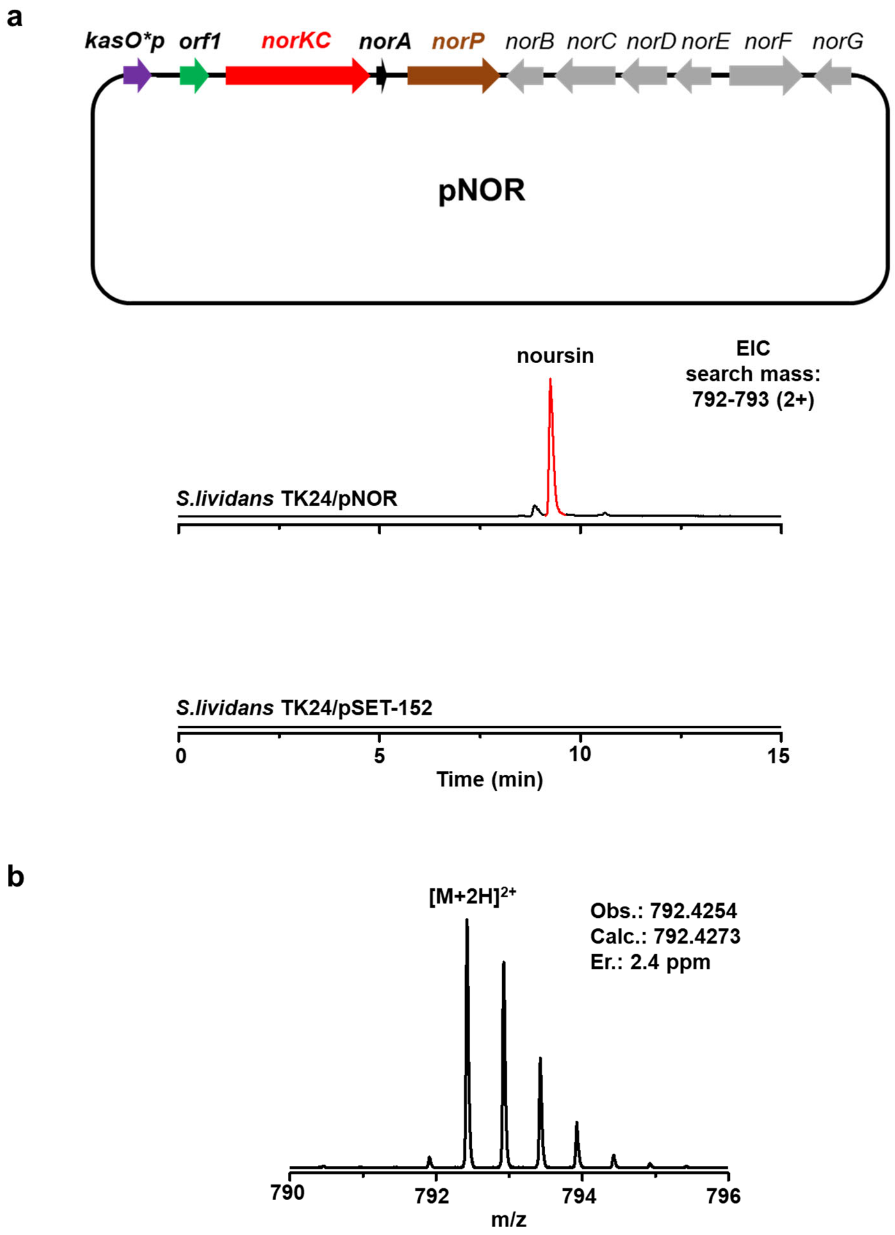

Supplementary Figure 1. Heterologous expression of the *nor* BGC and MS analysis of noursin. (a). The pNOR cosmid contains the complete *nor* BGC; LC-MS analysis of the extracts of *S. lividans* TK24/pNOR showing the production of noursin. *S. lividans* TK24/pSET-152 was employed as a control. (b). Mass spectra of noursin.

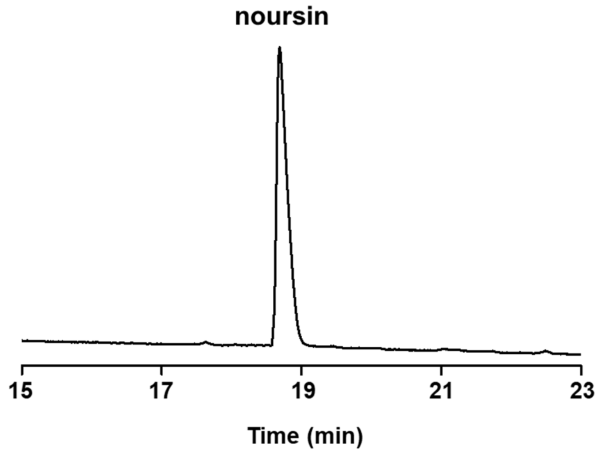

Supplementary Figure 2. HPLC analysis of the purified noursin sample. The analysis by HPLC was on an Ultimate Polar RP column (250 × 4.6 mm, 5 μm, Welch Technology Co., Ltd., Shanghai) by gradient elution of solvent A (H_2O with 0.1% formic acid) and solvent B (acetonitrile with 0.1% formic acid) with a flow rate of 1.0 mL/min over a 35 min period as follows: T = 0 min, 25% B; T = 5 min, 25% B; T = 25 min, 50% B; T = 27 min, 98% B; T = 33 min, 98% B; and T = 35 min, 25% B. The retention time of noursin is 18.7 min. The spectra was monitored at 200 nm.

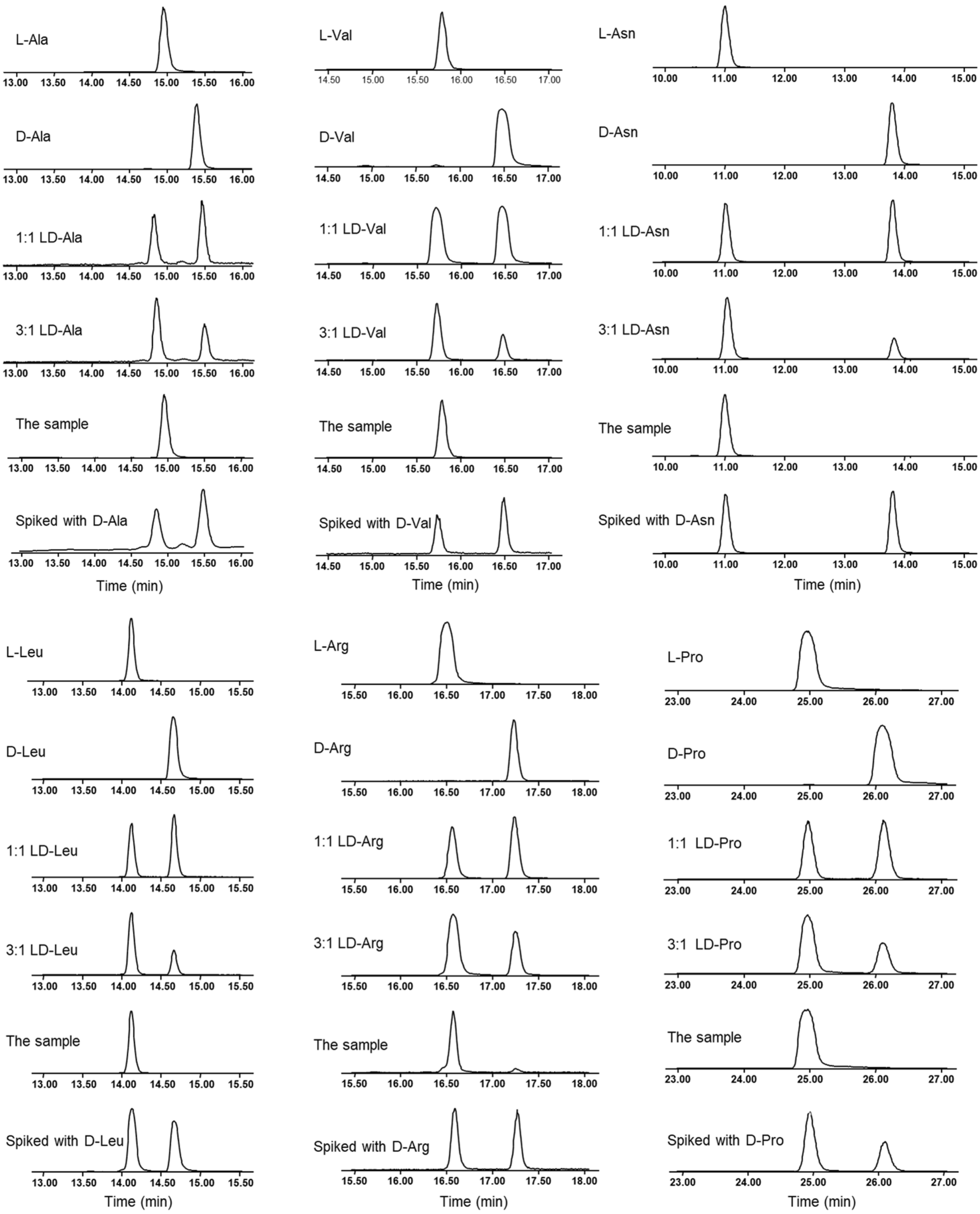

Supplementary Figure 3. Marfey's analysis of noursin. The Lab and Hbt derivatives were not detected under this analytical conditions of LC-MS.

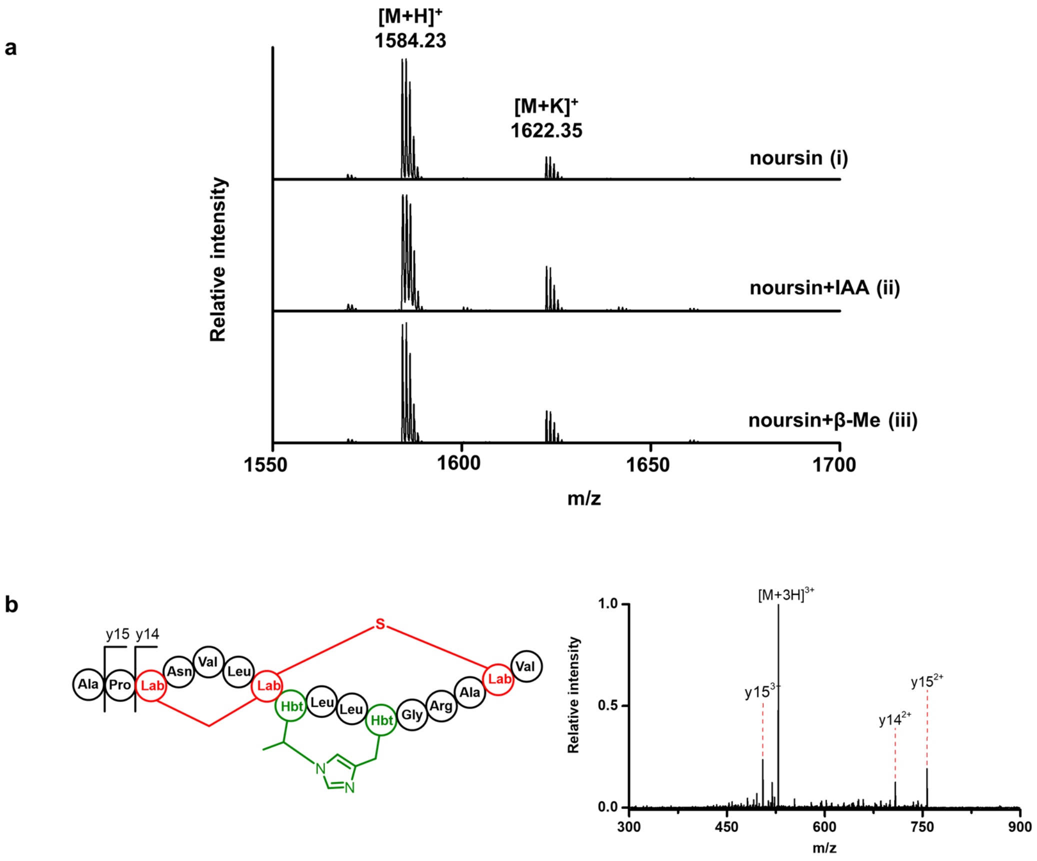

Species	Predicted Mass/Da	Observed Mass/Da	Error/ ppm	Species	Predicted Mass/Da	Observed Mass/Da	Error/ ppm	Species	Predicted Mass/Da	Observed Mass/Da	Error/ ppm
$y14^{2+}$	708.3824	708.3790	4.8	$y15^{3+}$	504.9417	504.9394	4.6	$y15^{2+}$	756.9088	756.9068	2.6

Supplementary Figure 4. MS and MS/MS analysis of noursin. (a). MALDI-TOF-MS analysis of noursin treated with IAA and βME. No adduct formation was observed. Assay conditions: (i) Noursin was incubated in 20 mM Tris-HCl, pH 8.0, for 1 hour at room temperature. Noursin: $M_{obs.}$ =1584.23 Da, $M_{calc.}$ =1583.84 Da. (ii) Noursin was incubated in 20 mM Tris-HCl, pH 8.0, with 0.5 mM IAA for 30 min at room temperature. (iii) Noursin was incubated in 20 mM Tris-HCl, pH 8.0, with 0.5 mM βME for 1 hour at 37 °C; (b). LC-MS/MS analysis of noursin. The *b* and *y* ions are listed in table and marked in the spectrum.

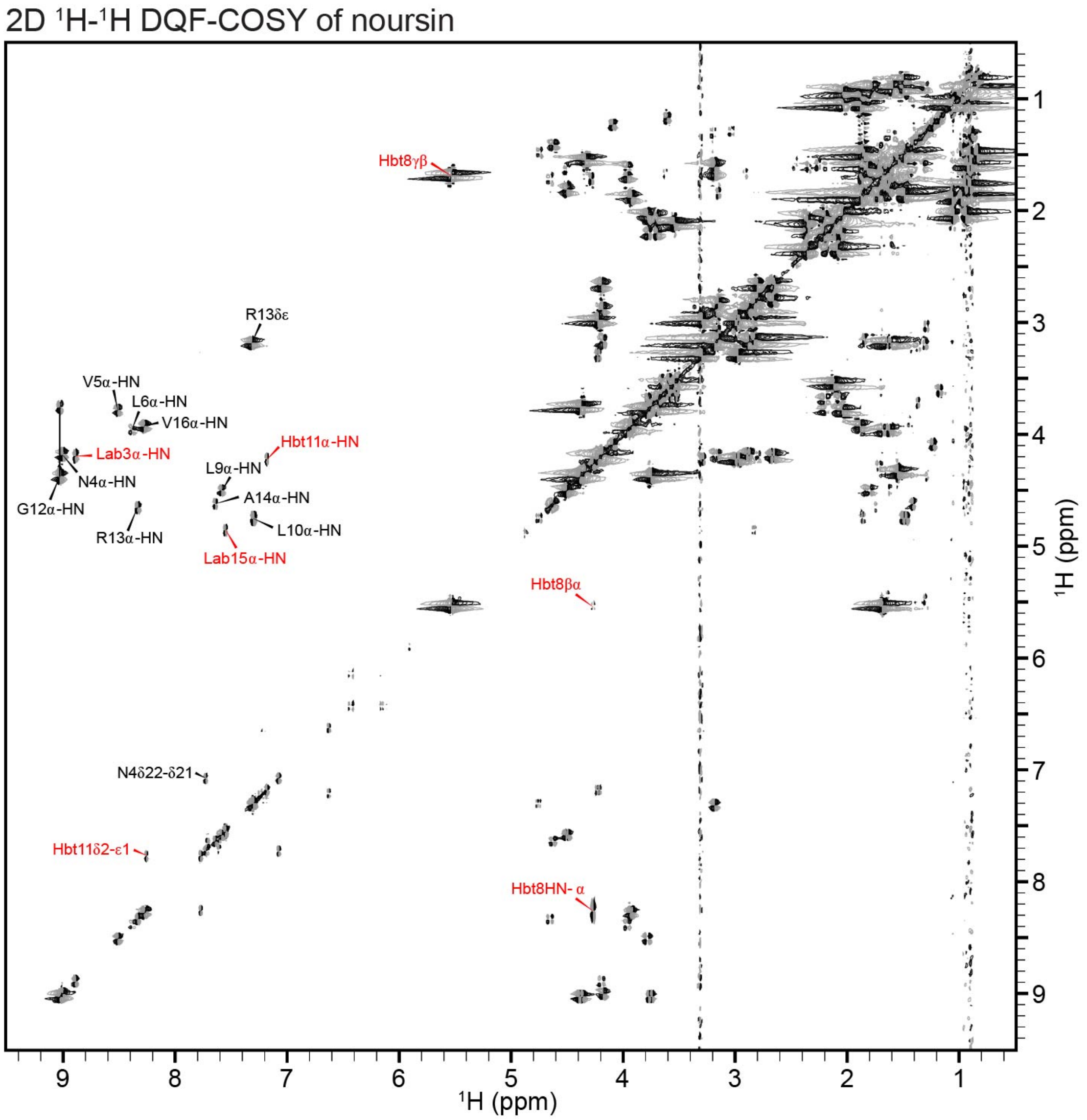

Supplementary Figure 5. 2D ^{1}H-^{1}H DQF-COSY spectrum of noursin in CD_3OH at 298 K. The assignments of crosslink-related residues were highlighted in red. No Hα signal was observed for the 7th residue. Unnatural amino acids, including Lab and Hbt, are indicated in three letter abbreviation. Natural amino acids are indicated in one letter abbreviation.

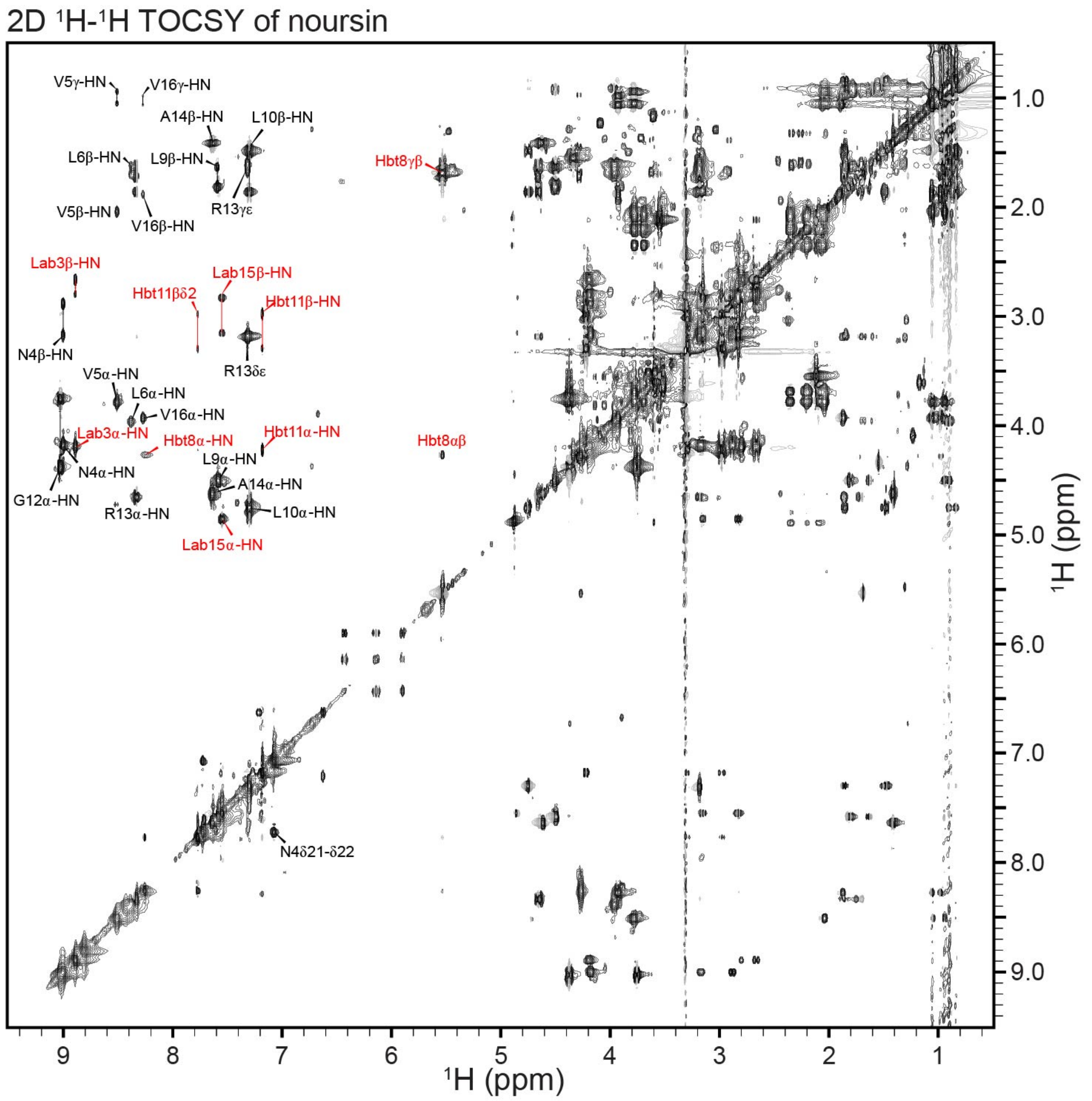

Supplementary Figure 6. 2D ^{1}H-^{1}H TOCSY spectrum of noursin in CD_3OH at 298 K with 30 ms of mixing time. The assignments of crosslink-related residues (Lab3, Lab7, Hbt8, Hbt11 and Lab15) were highlighted in red. Unnatural amino acids, including Lab and Hbt, are indicated in three letter abbreviation. Natural amino acids are indicated in one letter abbreviation.

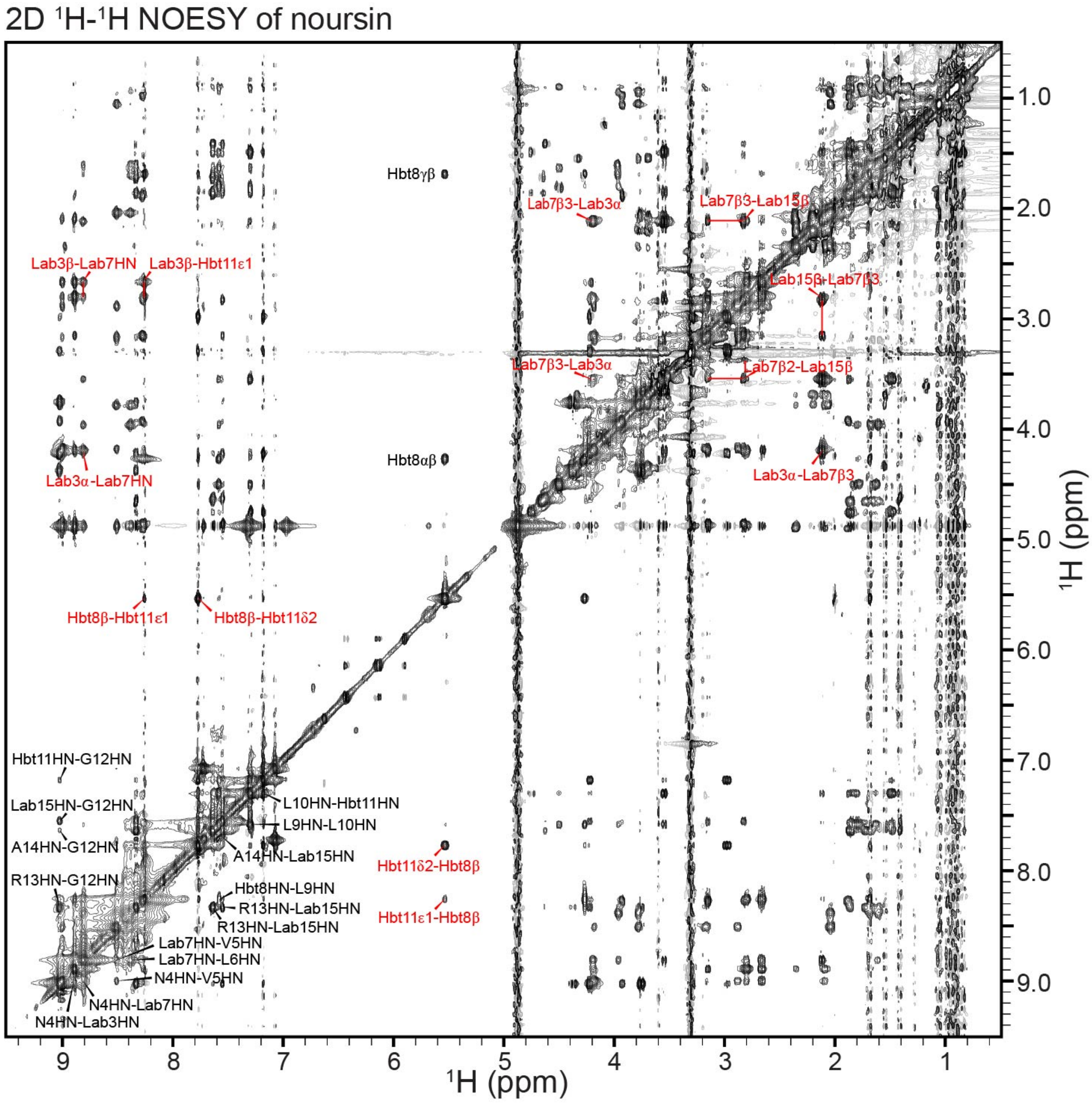

Supplementary Figure 7. 2D ^{1}H-^{1}H NOESY spectrum of noursin in CD_3OH at 298 K with 500 ms of mixing time. The strong NOE cross-peaks of the 3rd-to-7th, 7th-to-15th, and 8th-to-11th were observed abundantly and highlighted in red. Unnatural amino acids, including Lab and Hbt, are indicated in three letter abbreviation. Natural amino acids are indicated in one letter abbreviation.

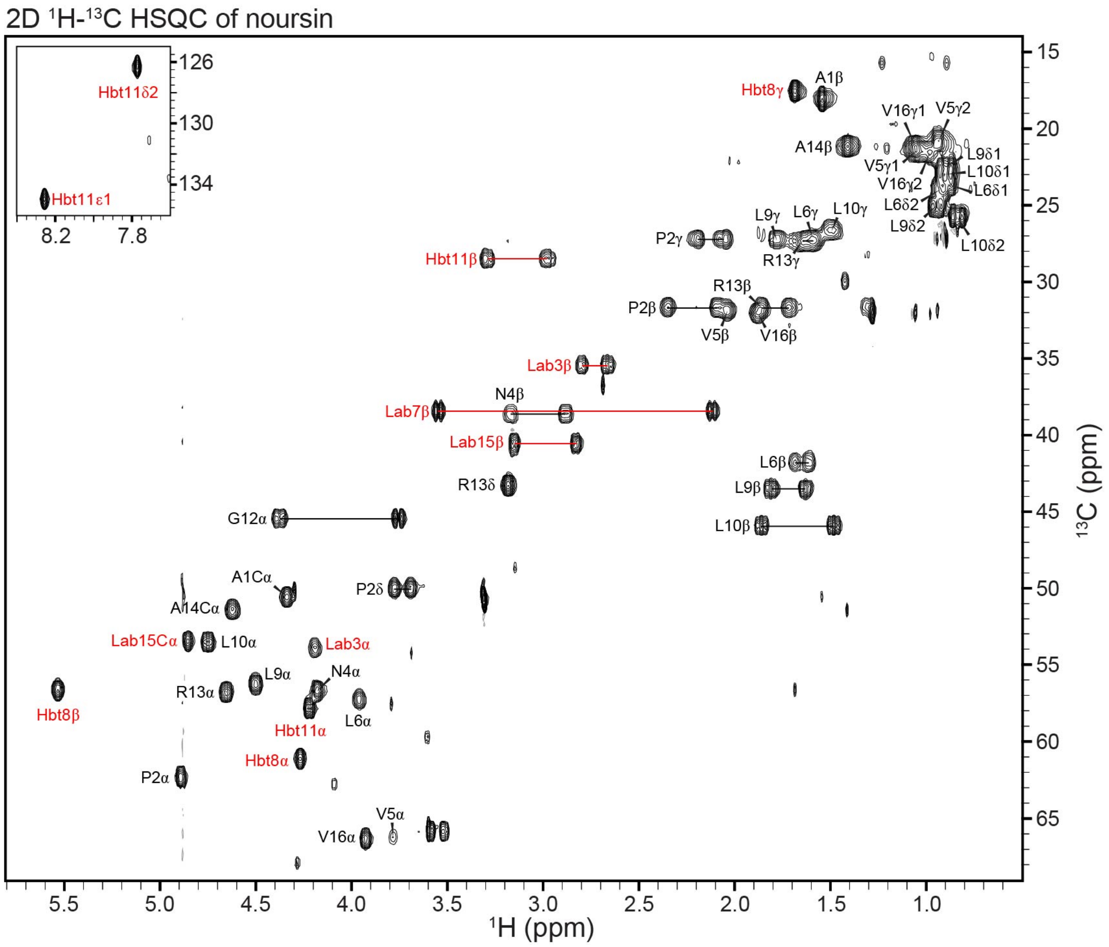

Supplementary Figure 8. 2D ^{1}H-^{13}C HSQC spectrum of noursin in CD_3OH at 298 K. The assignments of crosslinked residues (Lab3, Lab7, Hbt8, Hbt11 and Lab15) were highlighted in red. Except for Lab7, the Cα-Hα peaks of other residues were all observed. Unnatural amino acids, including Lab and Hbt, are indicated in three letter abbreviation. Natural amino acids are indicated in one letter abbreviation.

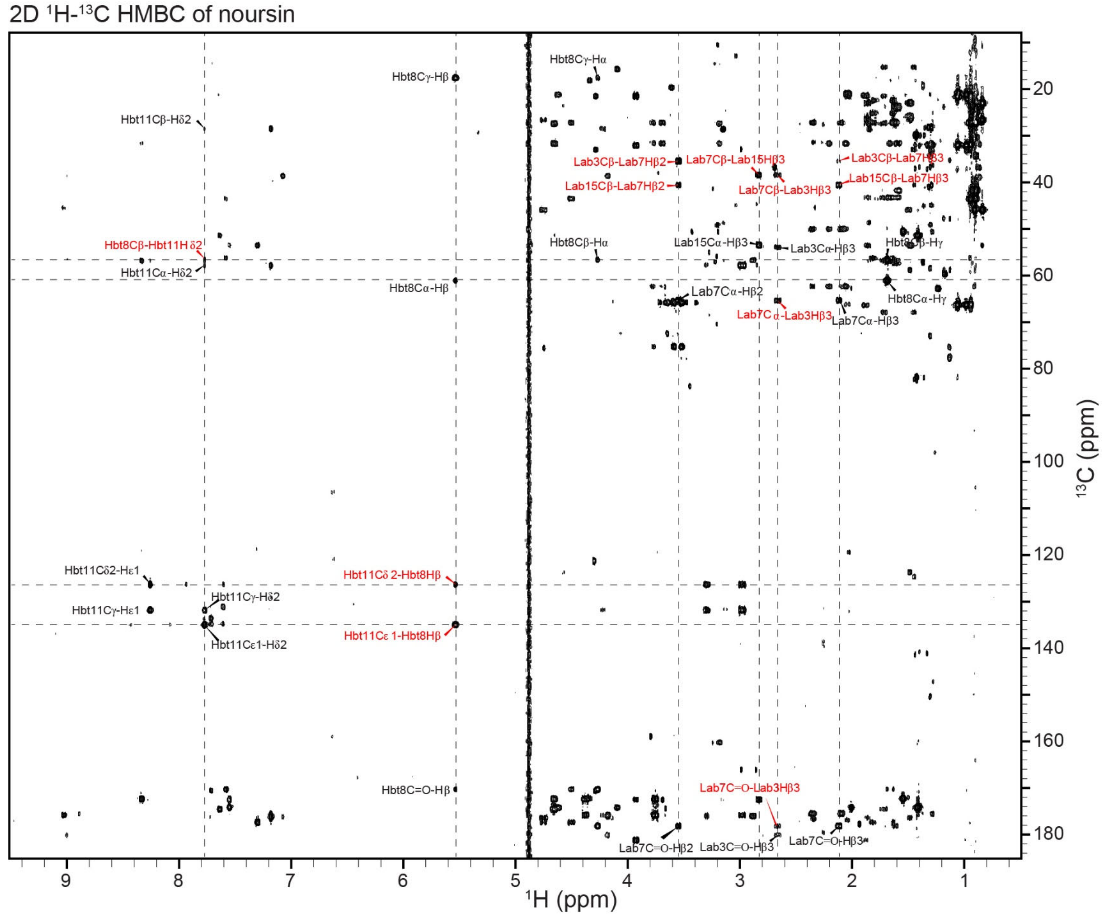

Supplementary Figure 9. 2D ^{1}H-^{13}C HMBC spectra of noursin in CD_3OH at 298 K. **a**, The heteronuclear cross-peaks of the 3rd-to-7th, 7th-to-15th, and 8th-to-11th were observed abundantly and highlighted in red. Unnatural amino acids, including Lab and Hbt, are indicated in three letter abbreviation. Natural amino acids are indicated in one letter abbreviation.

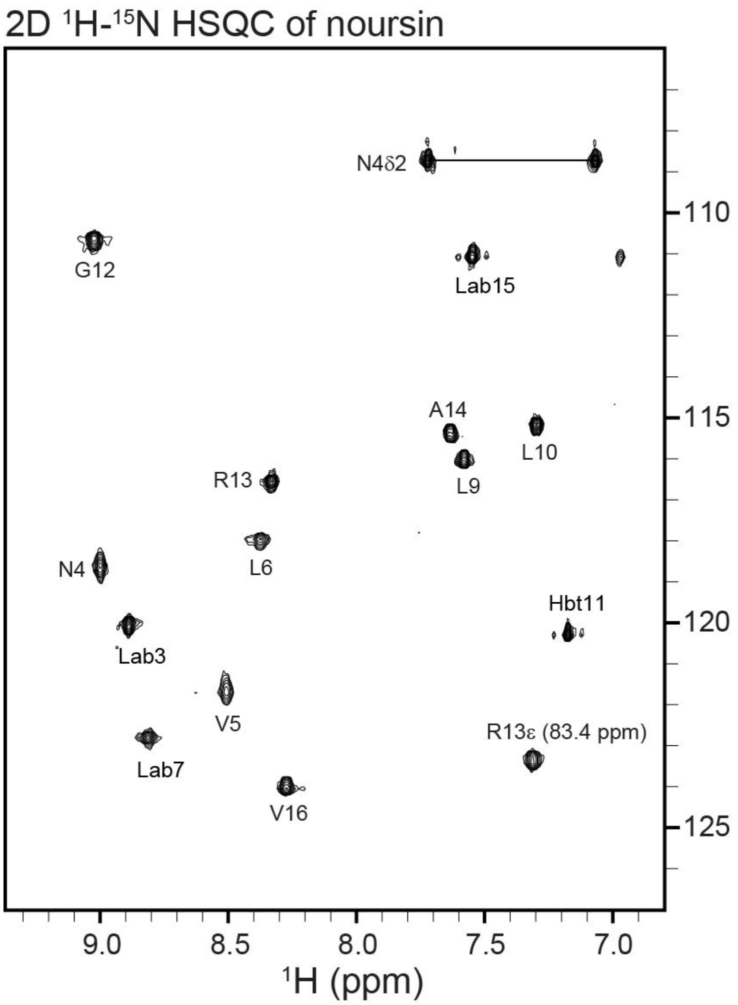

Supplementary Figure 10. 2D ^{1}H-^{15}N HSQC spectra of noursin in CD_3OH at 298 K. Except for the 1st, 2nd and 8th residues, signals of all the other residues were observed. Unnatural amino acids, including Lab and Hbt, are indicated in three letter abbreviation. Natural amino acids are indicated in one letter abbreviation.

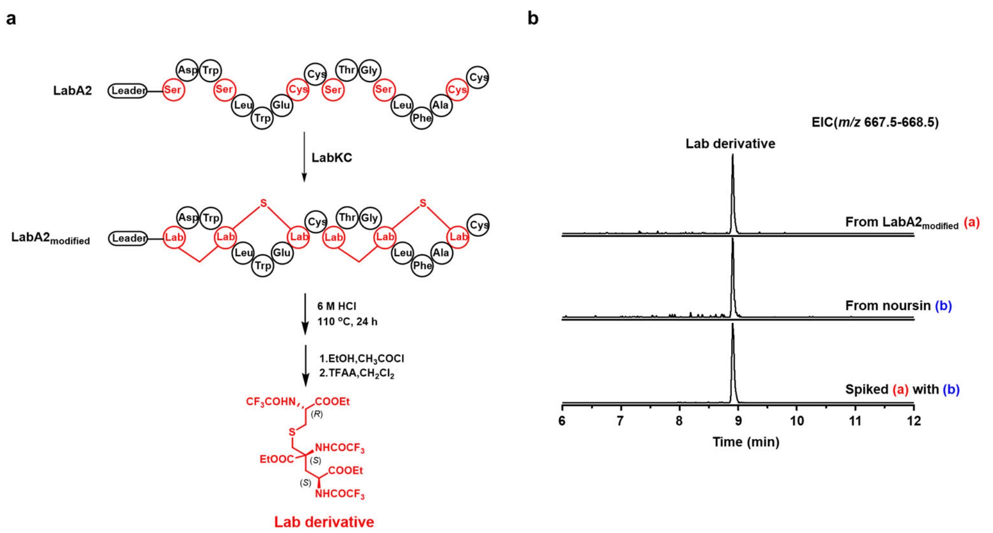

Supplementary Figure 11. The LC-MS analysis of the Lab derivatives of noursin and LabA2$_{\text{modified}}$. (a) LabA2$_{\text{modified}}$ was prepared by the modification of LabA2 by LabKC from the biosynthesis of labyrinthopeptin A2 *in vitro*. Assay conditions: (i) 20 mM Tris-HCl buffer (pH 8.0), 5 mM ATP, 1 mM $MgCl_2$, 0.1 mM TCEP, 50 µM LabA2, 50 µM LabKC. Noursin and LabA2$_{\text{modified}}$ were then hydrolyzed by 6 M HCl, modified by EtOH/CH_3COCl and trifluoroacetic anhydride to generate corresponding derivatives. (b) LC-MS analysis of the Lab derivatives of noursin and LabA2$_{\text{modified}}$. The extracted ion chromatogram of the Lab derivatives was shown with a mass window between m/z 667.5-668.5 Da. The signal peaks of the Lab derivatives generated from LabA2$_{\text{modified}}$ and noursin showed the same retention time of 8.9 min. The analysis by HPLC was performed on an ACQUITY UPLC C18 column (150 × 2.1 mm, 1.7 µm) by gradient elution of solvent A (H_2O with 0.1% formic acid) and solvent B (acetonitrile with 0.1% formic acid) with a flow rate of 0.2 mL/min over a 25 min period as follows: T = 0 min, 25% B; T = 20 min, 60% B; T = 25 min, 99% B.

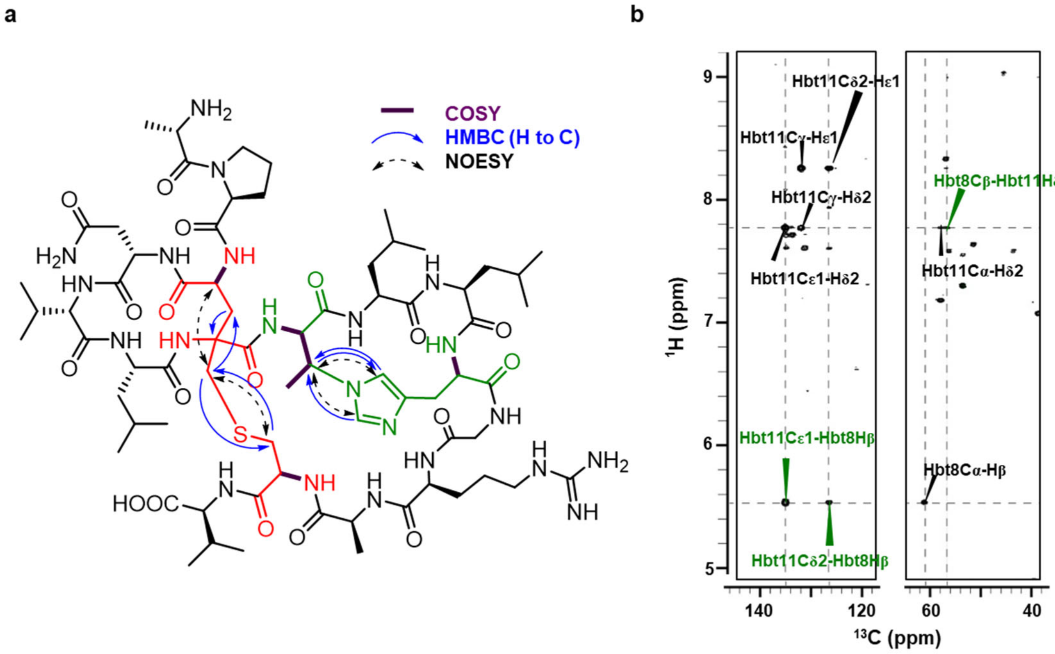

Supplementary Figure 12. The 2D ^{1}H-^{13}C HMBC spectrum showed the Hbt crosslink. (a). The chemical structure of noursin. The 2D NMR correlations of the Lab and the Hbt crosslink are shown. (b). Key heteronuclear cross-peaks of the Hbt crosslink in the 2D ^{1}H-^{13}C HMBC spectra of noursin.

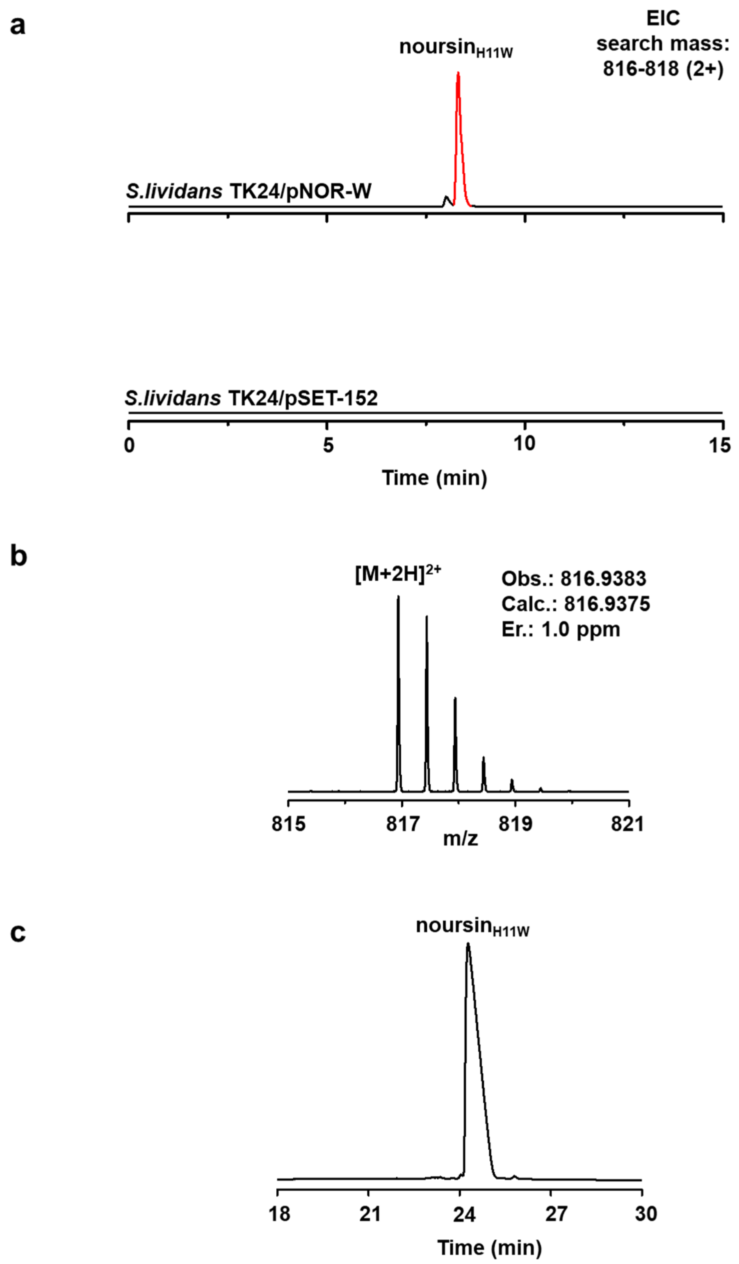

Supplementary Figure 13. Heterologous expression and isolation of noursin$_{H11W}$. (a). HR-HPLC-MS analysis of the extracts of *S. lividans* TK24/pNOR$_{H11W}$ and *S. lividans* TK24/pSET-152 as a control. The peak corresponding to noursin$_{H11W}$ is highlighted in red. (b). Mass spectra of noursin$_{H11W}$. The peak labelled in the spectra is monoisotopic peaks. (c). HPLC analysis of the purified noursin$_{H11W}$ sample. The analysis by HPLC was on an Ultimate Polar RP column (250 × 4.6 mm, 5 µm, Welch Technology Co., Ltd., Shanghai) by gradient elution of solvent A (H_2O with 0.1% formic acid) and solvent B (acetonitrile with 0.1% formic acid) with a flow rate of 1.0 mL/min over a 35 min period as follows: T = 0 min, 25% B; T = 5 min, 25% B; T = 25 min, 50% B; T = 27 min, 98% B; T = 33 min, 98% B; and T = 35 min, 25% B. The retention time of noursin is 24.6 min. The spectra was monitored at 200 nm.

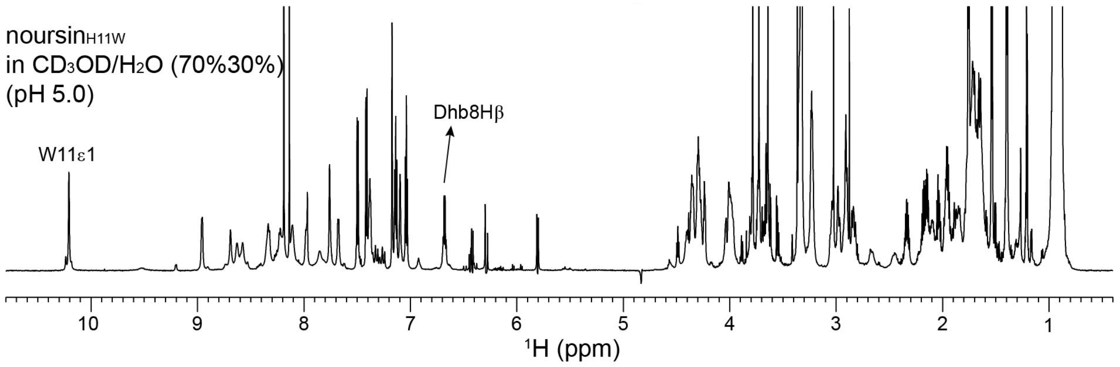

Supplementary Figure 14. ^{1}H NMR spectra of noursin$_{H11W}$ in CD_3OD/H_2O (70%/30%, pH ~5.0) at 298 K. Residues W11 and Dhb8 showed characteristic peaks in CD_3OD/H_2O, respectively.

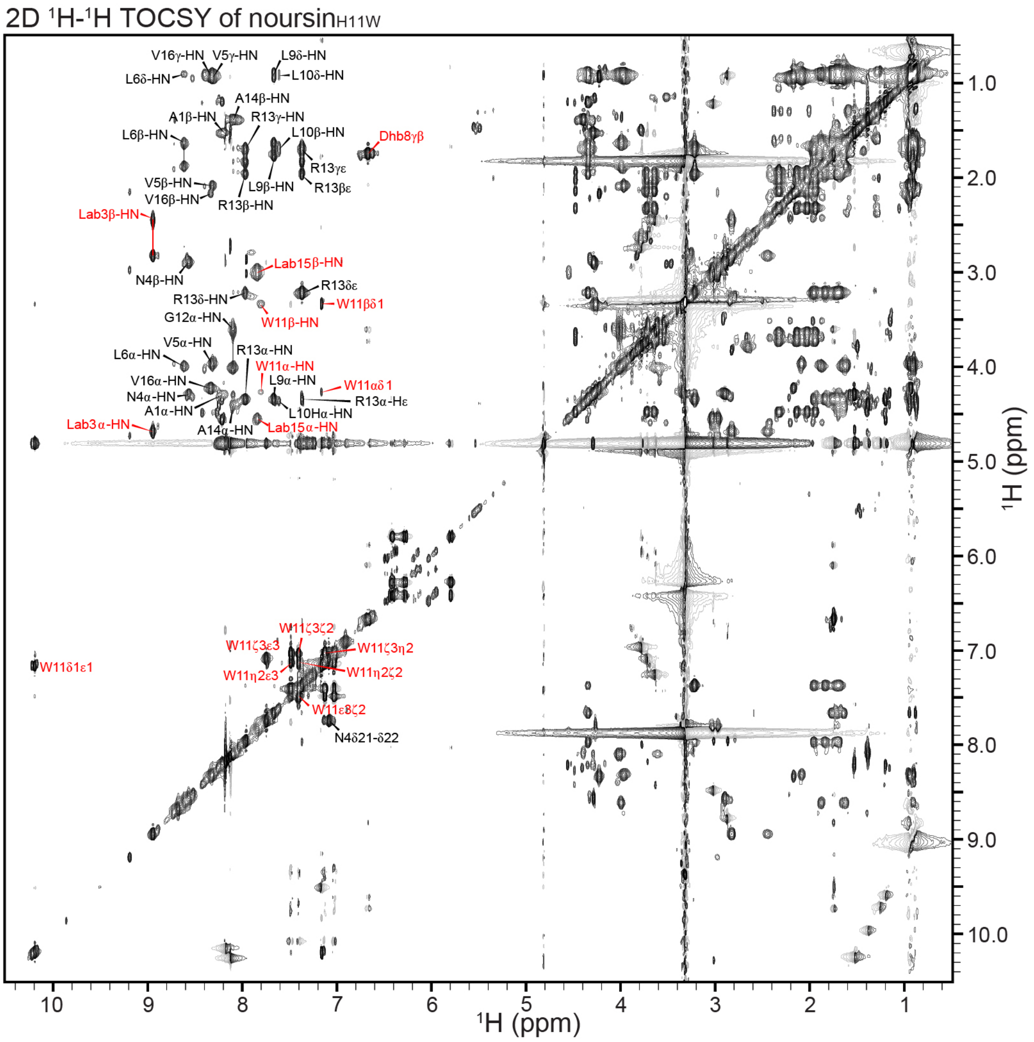

Supplementary Figure 15. 2D ^{1}H-^{1}H TOCSY spectrum of noursin$_{H11W}$ in CD_3OD/H_2O (70%/30%, pH ~5.0) at 298 K with 80 ms of mixing time. The assignments of Dhb8 and crosslink-related residues (Lab3, Lab7 and Lab15) were highlighted in red.

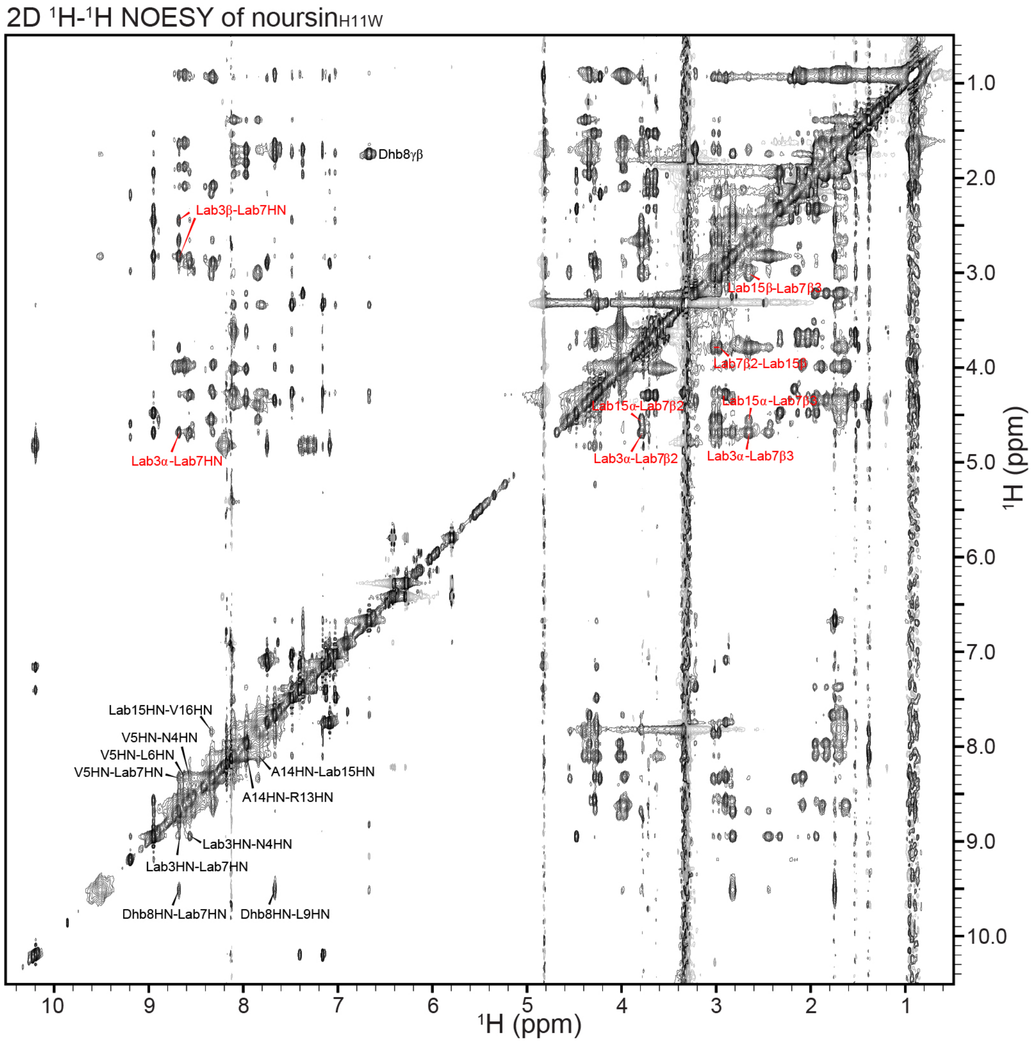

Supplementary Figure 16. 2D ^{1}H-^{1}H NOESY spectrum of noursin$_{H11W}$ in CD_3OD/H_2O (70%/30%, pH ~5.0) at 298 K with 500 ms of mixing time. The strong NOE cross-peaks of the 3rd-to-7th and 7th-to-15th were observed abundantly and highlighted in red.

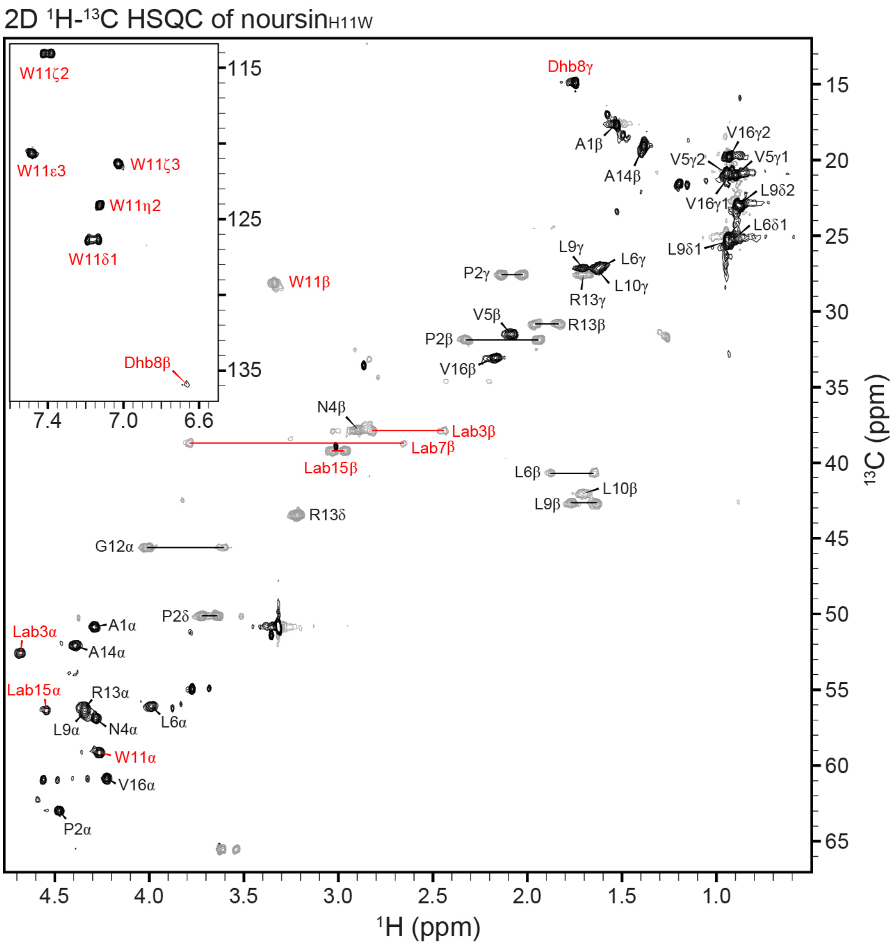

Supplementary Figure 17. 2D ^{1}H-^{15}N HSQC spectrum of noursin$_{H11W}$ in CD_3OD/H_2O (70%/30%, pH ~5.0) at 298 K. The assignments of Dhb8, W11, and the crosslinked residues (Lab3, Lab7, and Lab15) were highlighted in red. Signals of Dhb8 and the Lab crosslink are indicated in three letter abbreviation. Natural amino acids are indicated in one letter abbreviation.

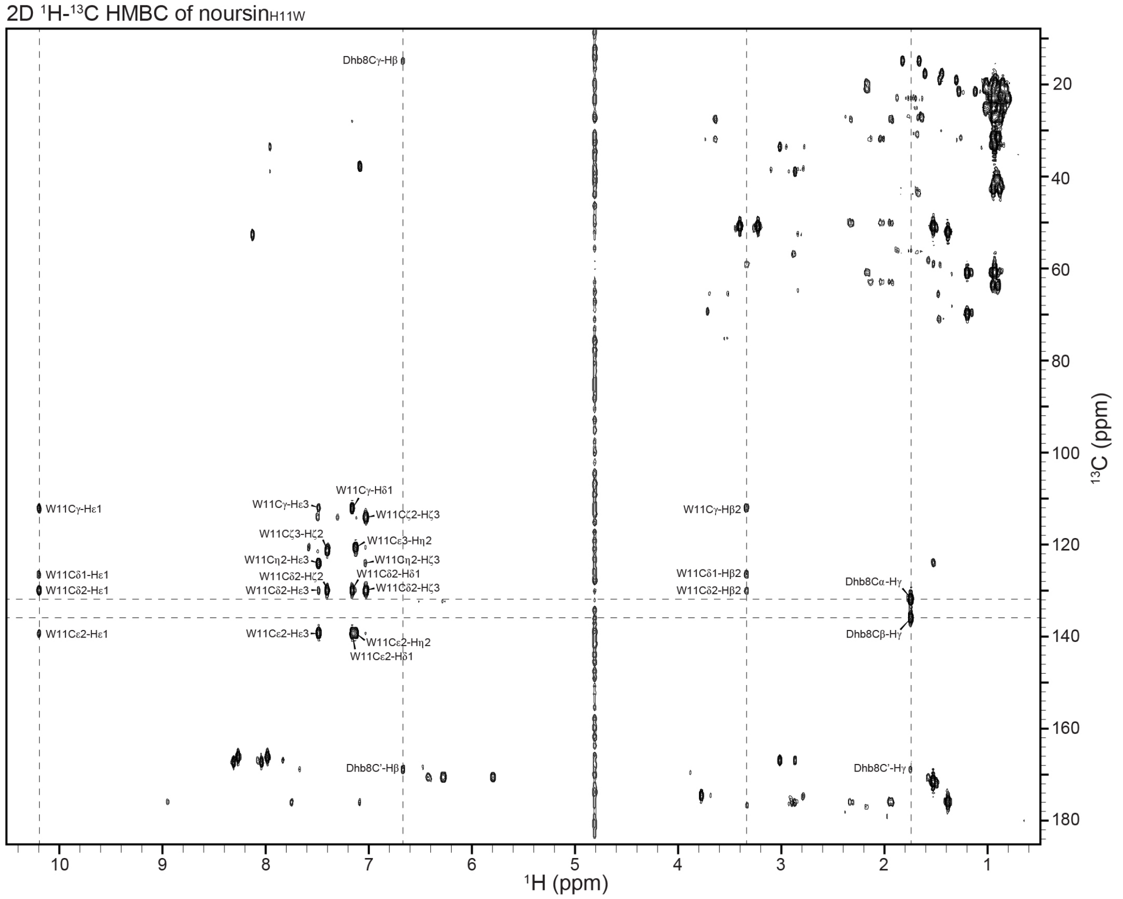

Supplementary Figure 18. 2D ^1H-^{13}C HMBC spectrum of noursin$_{H11W}$ in CD_3OD/H_2O (70%/30%, pH ~5.0) at 298 K. No cross peaks were observed for the 8th-to-11th residues, although most of resonances could be assigned. The chemical shifts of Dhb8Cα (131.8 ppm) and Dhb8Cβ (135.9 ppm) match the Cα=Cβ double bond situation well.

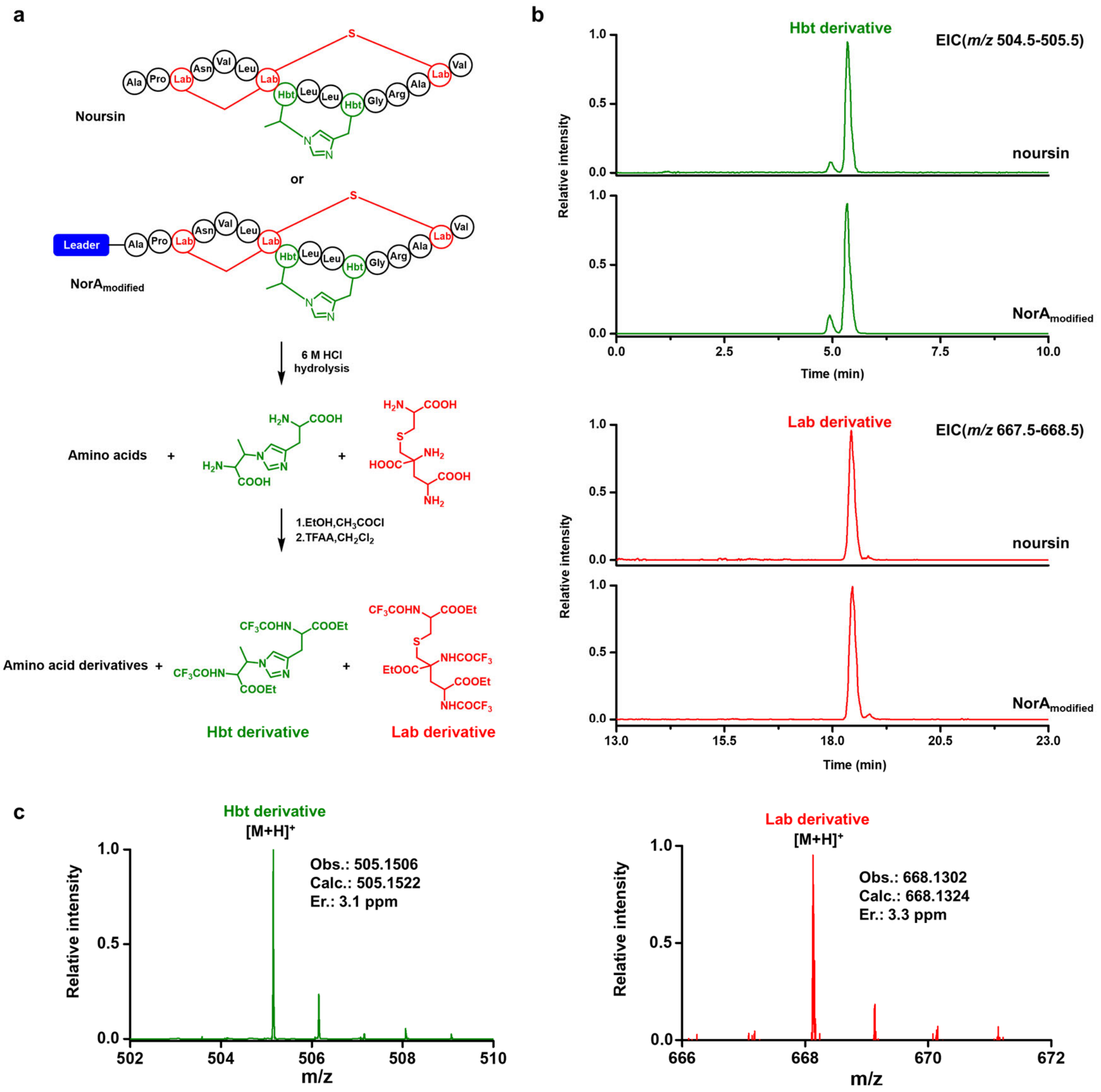

Supplementary Figure 19. LC-MS analysis of the Lab and the Hbt crosslinks in noursin and NorA$_{modified}$. (a) Noursin and NorA$_{modified}$ were hydrolyzed by 6 M HCl, modified by EtOH/CH_3COCl and trifluoroacetic anhydride to generate corresponding derivatives; (b) LC-MS analysis of the derivatized hydrolysates of noursin and NorA$_{modified}$ indicating the presence of the Lab and the Hbt crosslinks. The extracted ion chromatogram of the Hbt derivative with a mass window between m/z 504.5-505.5 Da. A peak with the retention time of 5.3 min was identified as the Hbt derivative. A minor peak shares the same mass-to-charge ratio with the retention time of 4.9 min was detected with a peak area of 8 % of that of the Hbt derivative. The extracted ion chromatogram of the Lab derivative with a mass window between m/z 667.5-668.5. The peak with the retention time of 18.3 min was identified as the Lab derivative. (c) MS spectra of the Hbt derivative and the Lab derivative. Hbt derivative: Obs. $[M+H]^+$ = 505.1506, Cacl.: $[M+H]^+$ = 505.1522, error = 3.1 p.p.m. Lab derivative: Obs. $[M+H]^+$ = 668.1302, Cacl.: $[M+H]^+$ = 668.1324, error = 3.3 p.p.m. HPLC analysis was performed on an ACQUITY UPLC C18 column (150 × 2.1 mm, 1.7 µm) by gradient elution of solvent A (H_2O with 0.1% formic acid) and solvent B (acetonitrile with 0.1% formic acid) with a flow rate of 0.2 mL/min over a 25 min period as follows: T = 0 min, 25% B; T = 20 min, 60% B; T = 25 min, 99% B.

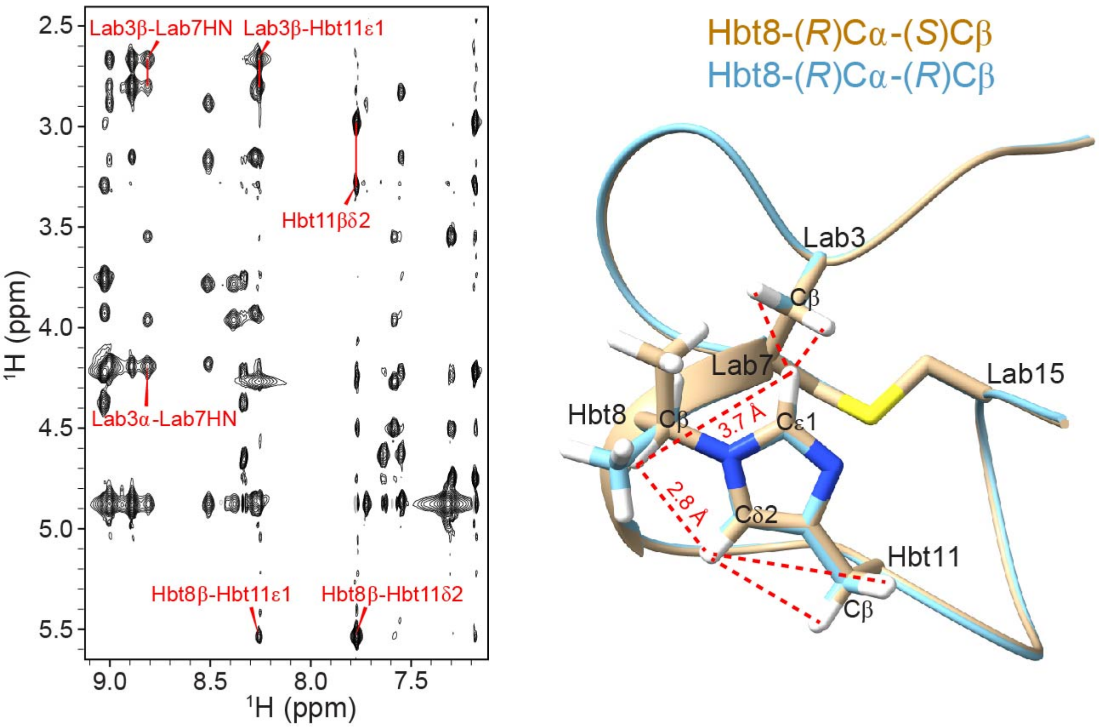

Supplementary Figure 20. The assignment of Cβ of Hbt8 in the *S* configuration. The local region of 2D ^{1}H-^{1}H NOESY spectrum of noursin was shown (left). The structures of noursin at the *R* (orange) and *S* (cyan) configurations of Hbt8 were aligned. The Hbt8(Hβ) exhibits stronger intensity of NOE peak to Hbt11(Hδ2) than to Hbt11(Hε1), indicating a shorter distance between Hbt11(Hδ2) and Hbt8(Hβ) than that between Hbt11(Hε1) and Hbt8(Hβ), and therefore matches the *S* configuration of Hbt8(Cβ).

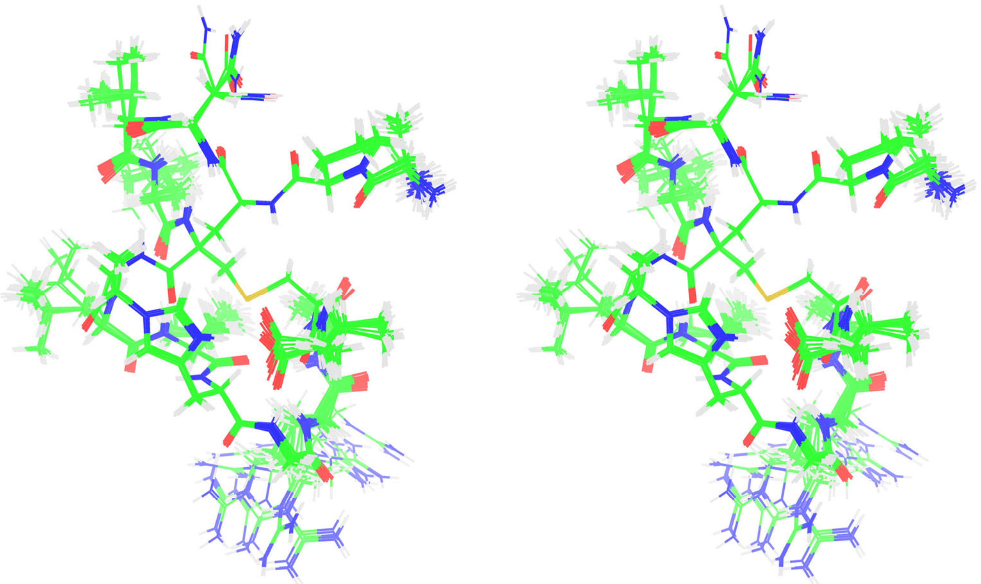

Supplementary Figure 21. A cross-eye stereo of the superimposed 20 lowest energy structures of noursin.

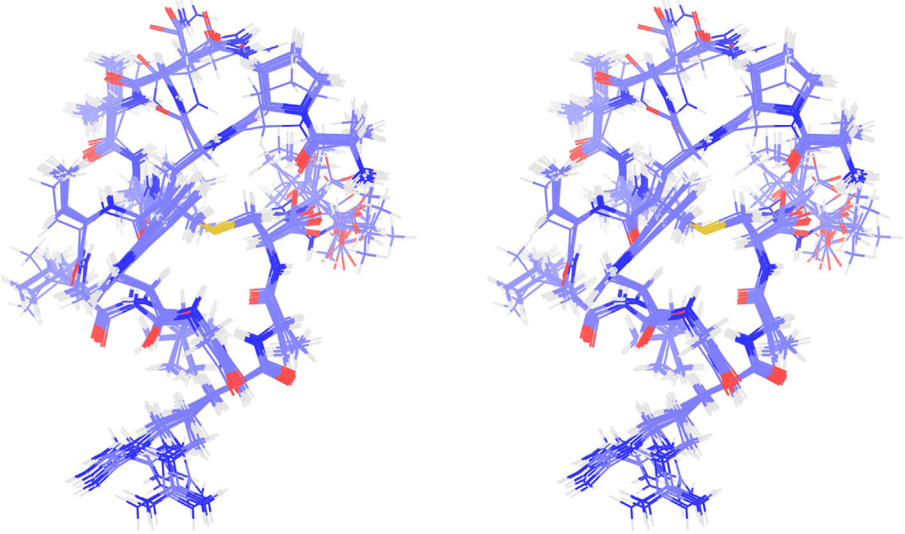

Supplementary Figure 22. A cross-eye stereo of the superimposed 20 lowest energy structures of noursin$_{H11W}$.

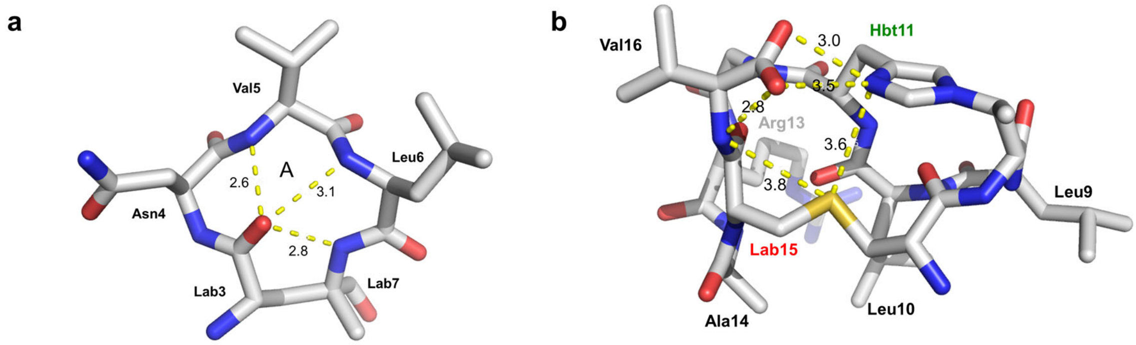

Supplementary Figure 23. The structure of ring A (a) and C-terminal residues (b) in the lowest energy conformer of noursin and the hydrogen bonding network.

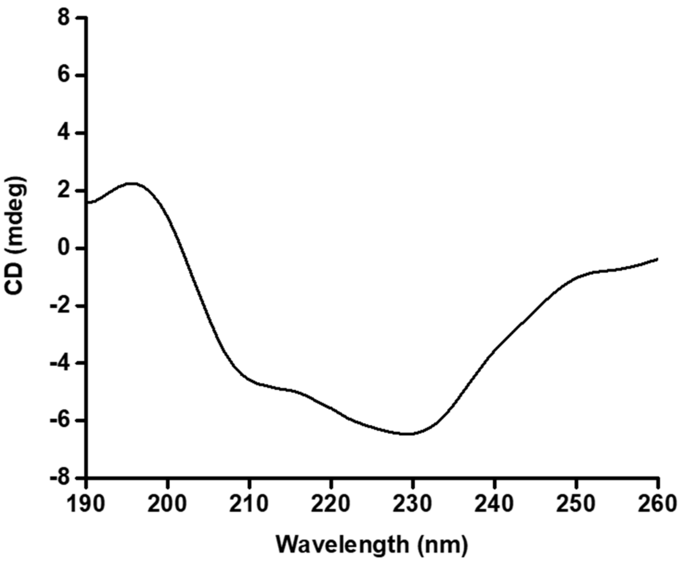

Supplementary Figure 24. The circular dichroism spectra of noursin in H_2O at 298 K.

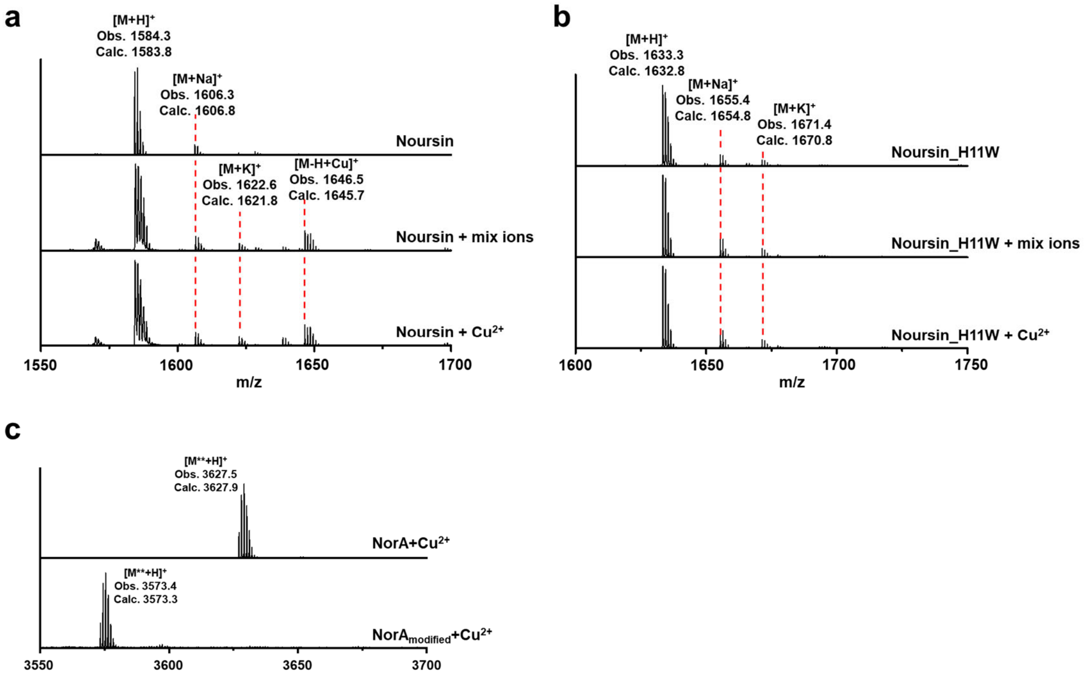

Supplementary Figure 25. The metal binding ability of noursin and its derivatives analyzed by MALDI-TOF-MS. (a). Noursin binds to Cu ions with specificity. The solution of mixed ions contains 1 mM $Fe_2(SO_4)_3$, $AlCl_3$, $CuSO_4$, $ZnCl_2$, $NiCl_2$, $MgSO_4$ and $MnCl_2$. (b). $Noursin_{H11W}$ does not bind to metal ions tested. (c). The modification of NorA by NorKC and the leader removal are both required for the copper-binding activity of noursin. MALDI-TOF-MS analysis of the NorA and $NorA_{modified}$ incubated with $CuSO_4$. Assay conditions: 50 µM NorA or $NorA_{modified}$, 50 µM $CuSO_4$. NorA: $M_{obs.}$ =3627.52 Da, $M_{calc.}$ =3627.87 Da. $NorA_{modified}$: $M_{obs.}$ =3573.43 Da, $M_{calc.}$ =3573.84 Da.

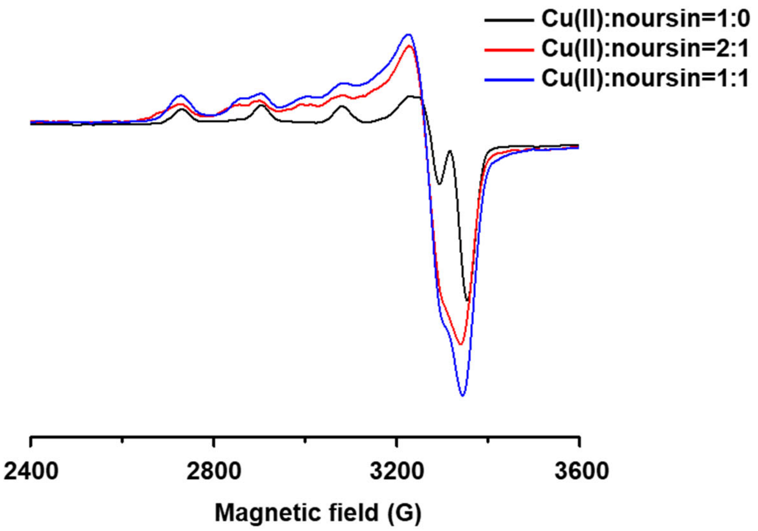

Supplementary Figure 26. The electro paramagnetic resonance (EPR) spectra of the mixed samples of noursin with $CuSO_4$ at 298 K. EPR spectra were acquired of the $CuSO_4$ (2 mM) incubated with 0, 0.5 and 1.0 equiv. of noursin for >10 min at room temperature in 20 mM HEPES buffer, pH 7.5, prior to freezing in liquid nitrogen. The spectra (20 K) was recorded at X-band on a Bruker EMX spectrometer

equipped with an ESR900 continuous flow cryostat (Oxford Instruments). The microwave frequency was 9.41 GHZ, the microwave power 10 mW, the field modulation 5 G, and the time constant and the conversion time were both 40.96 ms. Spectra were processed and simulated using WIN EPR and SIMFONIA (Bruker), respectively.

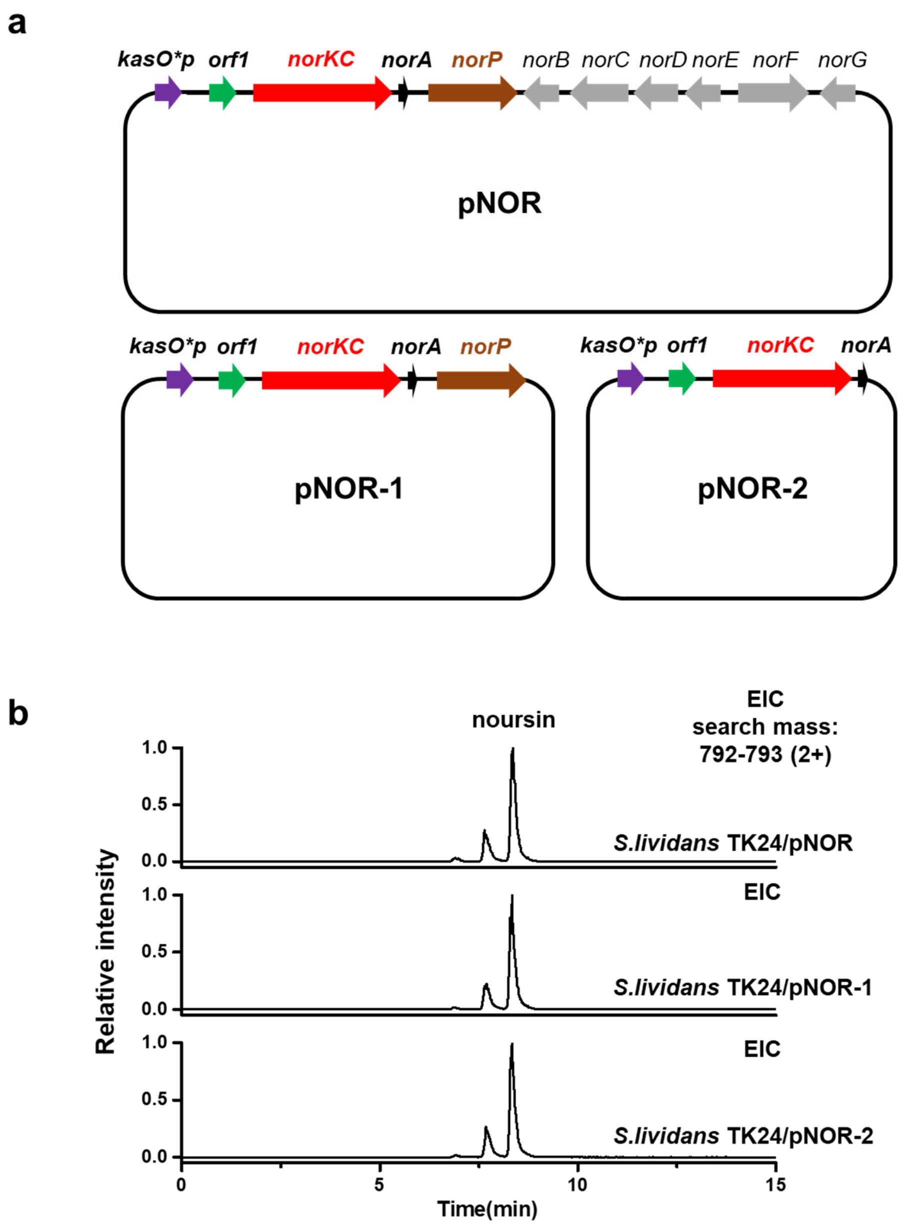

Supplementary Figure 27. Heterologous expression of the *nor* BGCs with gene deletions. (a). pNOR cosmids with the complete or partial *nor* BGC; (b). LC-HRMS analysis of the extracts of *S. lividans* TK24/pNOR, *S. lividans* TK24/ pNOR-1 and *S. lividans* TK24/ pNOR-2.

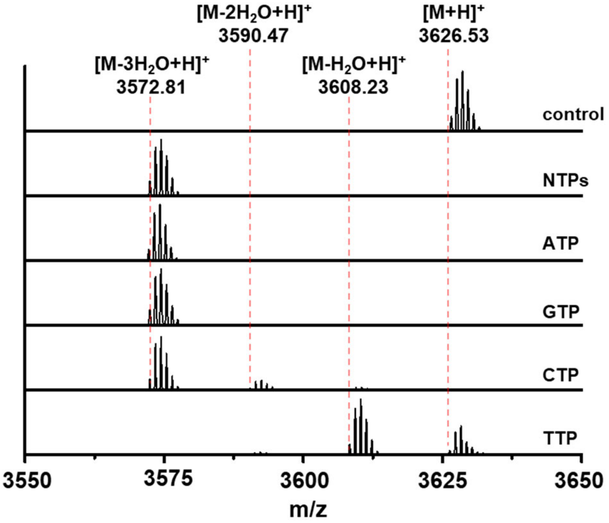

Supplementary Figure 28. Requirement of NTPs during the modification of NorA by NorKC. Assay conditions: 20 mM Tris-HCl buffer (pH 8.0), 5 mM NTPs, 1 mM $MgCl_2$, 0.1 mM TCEP, 50 μM NorA and 10 μM NorKC at 28 °C for 1 h. NorA: $M_{calc.}$=3627.87 Da, $M_{obs.}$=3626.53 Da; NorA-H_2O: $M_{calc.}$=3609.85 Da, $M_{obs.}$=3608.23 Da; NorA-2H_2O: $M_{calc.}$=3591.83 Da, $M_{obs.}$=3590.47 Da; NorA-3H_2O: $M_{calc.}$=3573.84 Da, $M_{obs.}$=3572.81 Da.

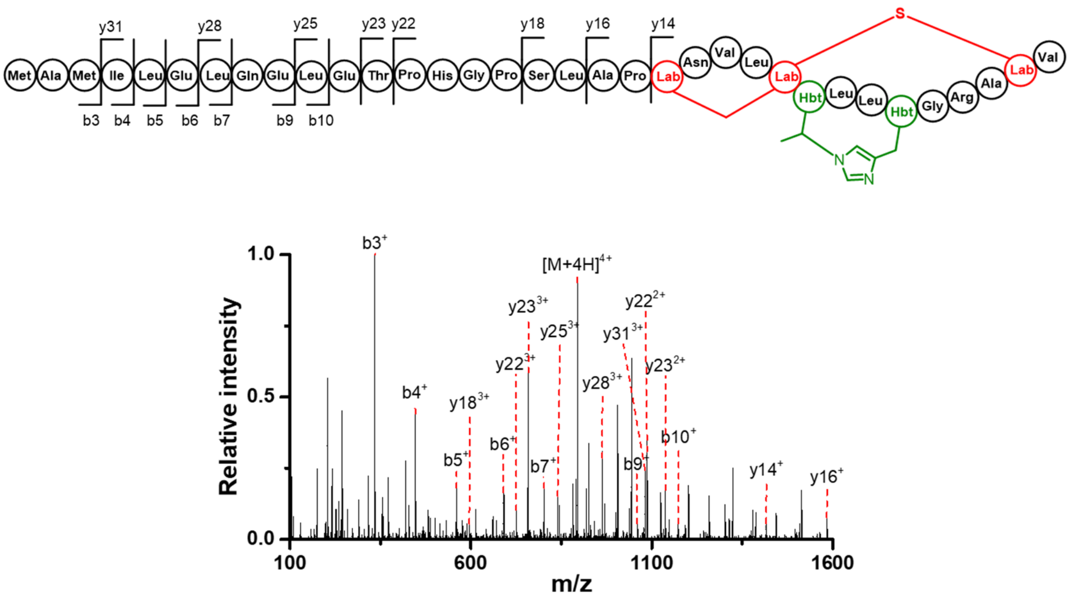

Species	Predicted Mass/Da	Observed Mass/Da	Error/ ppm	Species	Predicted Mass/Da	Observed Mass/Da	Error/ ppm
b3	334.1254	334.1261	5.0	y16	1583.8474	1583.8501	1.7
b4	447.2094	447.2108	3.1	$y18^{3+}$	595.3261	595.3234	4.5
b5	560.2935	560.2953	3.2	$y22^{3+}$	724.7213	724.7231	2.5
b6	689.3361	689.3389	4.1	$y23^{2+}$	1137.1022	1137.1055	3.0
b7	802.4201	802.4235	4.2	$y25^{3+}$	839.1128	839.1108	2.4
b9	1059.5213	1059.5212	0.9	$y28^{3+}$	962.5078	962.5125	4.9
b10	1172.6054	1172.6095	3.5	$y31^{3+}$	1080.9114	1080.9140	2.4
y14	1415.7575	1415.7586	0.8				

Supplementary Figure 29. Tandem MS analysis of $NorA_{modified}$ generated *in vitro*. The *b* and *y* ions are listed in table and marked in the spectrum.

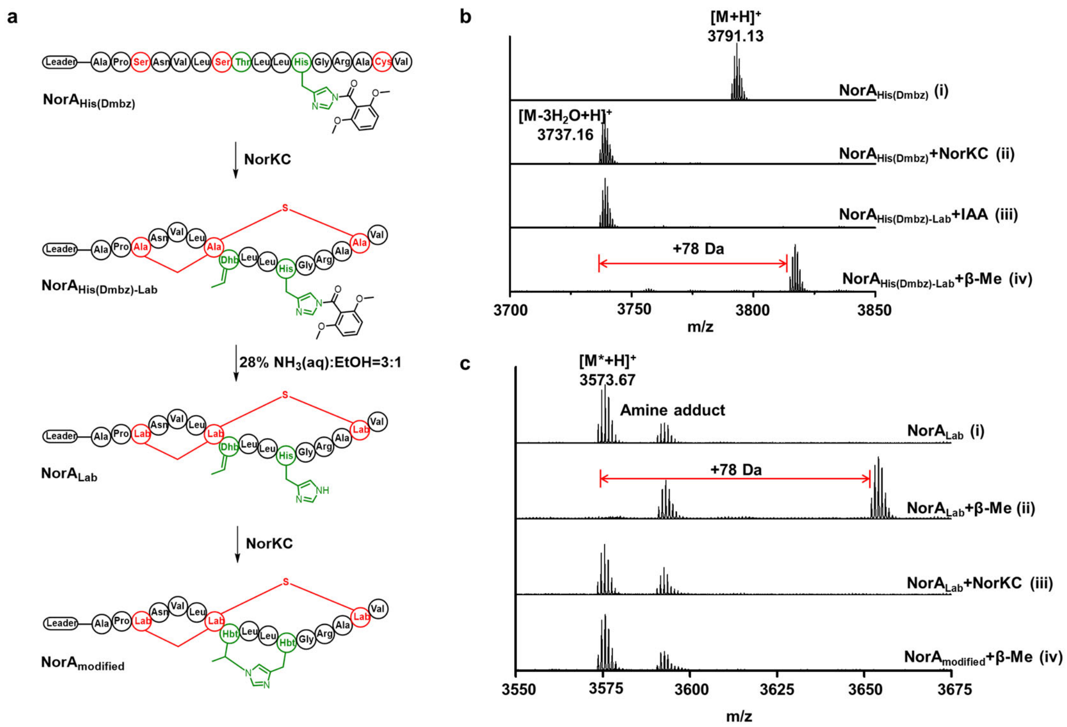

Supplementary Figure 30. The formation of the Lab motif catalyzed by NorKC is independent of the formation of the Hbt motif. (a) Schematic illustration of the assay design. (b) MALDI-TOF-MS analysis of the modification of $\mathrm{NorA}_{\mathrm{His(Dmbz)}}$ by NorKC *in vitro*. Assay conditions: (i) 20 mM Tris-HCl buffer (pH 8.0), 5 mM ATP, 1 mM $MgCl_2$, 0.1 mM TCEP, 50 µM $\mathrm{NorA}_{\mathrm{His(Dmbz)}}$, 10 µM NorKC. $\mathrm{NorA}_{\mathrm{His(Dmbz)}}$: $M_{\mathrm{obs.}}$ =3791.13 Da, $M_{\mathrm{calc.}}$ =3791.36 Da; (ii) $\mathrm{NorA}_{\mathrm{His(Dmbz)}}$ incubated with NorKC (10 µM) at 28 °C for 1 h. $\mathrm{NorA}_{\mathrm{His(Dmbz)\text{-}Lab}}$: $M_{\mathrm{obs.}}$ =3737.16 Da, $M_{\mathrm{calc.}}$ =3737.24 Da; (iii) $\mathrm{NorA}_{\mathrm{His(Dmbz)\text{-}Lab}}$ treated by IAA did not result in any mass change; (iv) $\mathrm{NorA}_{\mathrm{His(Dmbz)\text{-}Lab}}$ treated by β-Me led to the formation of β-Me adduct. (c) MALDI-TOF-MS analysis of the modification of $\mathrm{NorA}_{\mathrm{Lab}}$ by NorKC *in vitro*. Assay conditions: (i) $\mathrm{NorA}_{\mathrm{His(Dmbz)\text{-}Lab}}$ treated by ammonolysis reagent (28% NH_3: EtOH=3:1) at 25 °C for 1 h to remove the protecting group yielded $\mathrm{NorA}_{\mathrm{Lab}}$ ($M_{\mathrm{obs.}}$ =3573.67 Da, $M_{\mathrm{calc.}}$ =3573.84 Da); (ii) $\mathrm{NorA}_{\mathrm{Lab}}$ treated by β-Me led to the formation of β-Me adduct; (iii) $\mathrm{NorA}_{\mathrm{Lab}}$ incubated with NorKC (10 µM) at 28 °C for 1 h yielded $\mathrm{NorA}_{\mathrm{modified}}$; (iv) $\mathrm{NorA}_{\mathrm{modified}}$ treated by βMe did not result in any mass change.

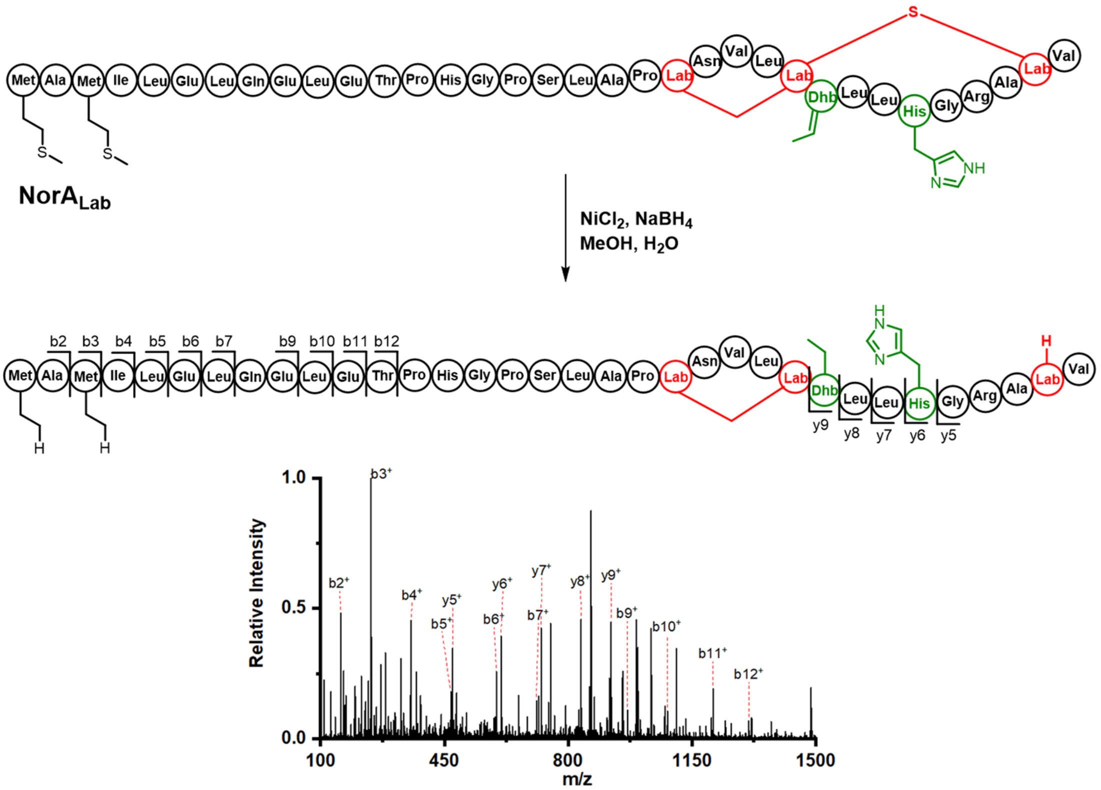

Species	Predicted Mass/Da	Observed Mass/Da	Error/ ppm	Species	Predicted Mass/Da	Observed Mass/Da	Error/ ppm
b2	157.0972	157.0972	0.0	b11	1209.6726	1209.6769	3.6
b3	242.1500	242.1503	1.2	b12	1310.7203	1310.7243	3.1
b4	355.2341	355.2342	0.3	y5	473.2831	473.2835	0.8
b5	468.3181	468.3177	0.9	y6	610.3420	610.3428	1.3
b6	597.3607	597.3614	1.2	y7	723.4261	723.4270	1.2
b7	710.4448	710.4461	1.8	y8	836.5101	836.5116	1.8
b9	967.5460	967.5484	2.5	y9	921.5629	921.5653	2.6
b10	1080.6300	1080.6333	3.1				

Supplementary Figure 31. NorA$_{Lab}$ was partially linearized through desulfurization and the LC-MS/MS analysis of linearized NorA$_{Lab}$. The *b* and *y* ions are listed in table and marked in the spectrum.

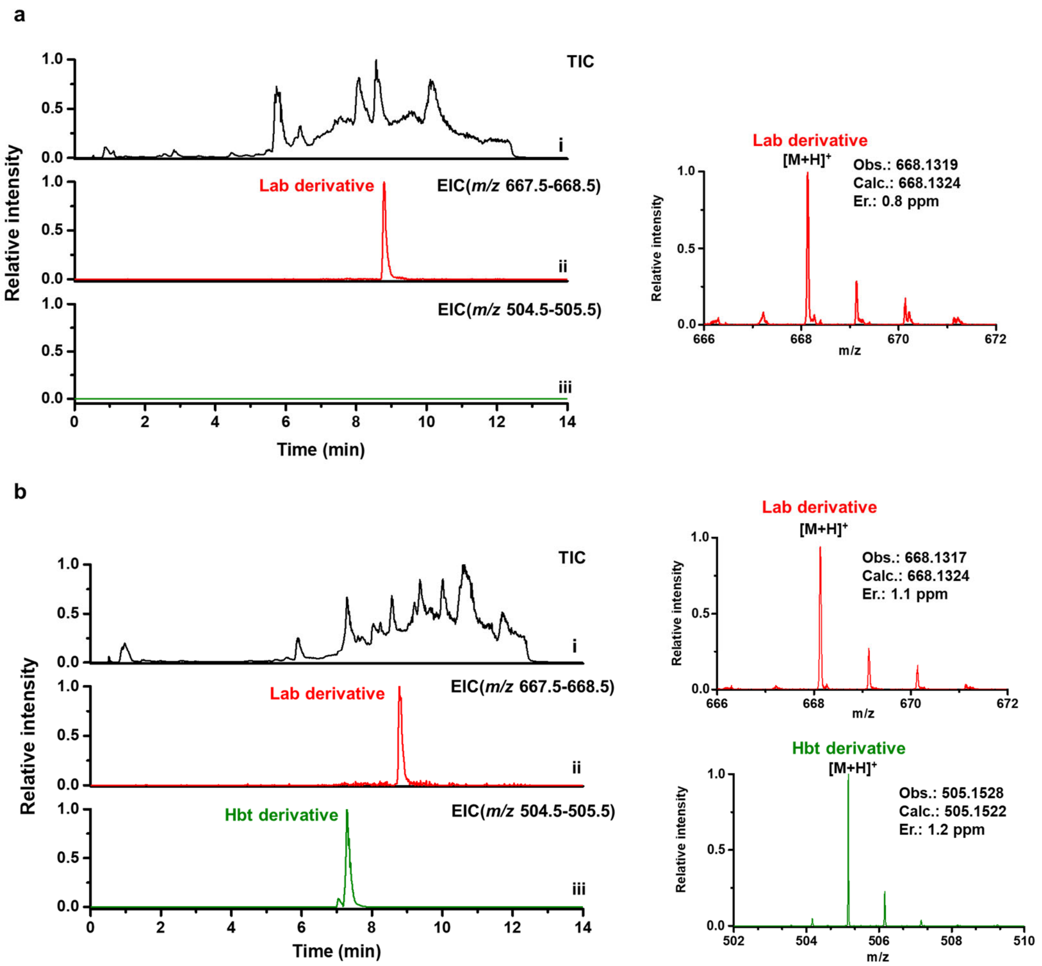

Supplementary Figure 32. LC-MS analysis of the Lab and the Hbt derivatives from NorA$_{Lab}$ and NorA$_{modified}$. (a) LC-MS analysis of the hydrolysate and *N*-trifluoroacetyl/ethylester derivatization of NorA$_{His(Dmbz)-Lab}$, and HRMS analysis of Lab derivative with $[M+H]^+$ = 668.1319 (Cacl.: $[M+H]^+$ = 668.1324, error = 0.8 p.p.m.). (i) The total ion chromatography; (ii) The extracted ion chromatogram of the Lab derivative with a mass window between m/z 667.5-668.5. A peak with the retention time of 8.9 min was identified as the Lab derivative; (iii) The extracted ion chromatogram of the Hbt derivative with a mass window between m/z 504.5-505.5; (b) LC-MS analysis of the derivatized hydrolysate of NorA$_{modified}$, and MS spectra of Lab derivative with $[M+H]^+$ = 668.1317 (Cacl.: $[M+H]^+$ = 668.1324, error = 1.1 p.p.m.), and Hbt derivative with $[M+H]^+$ = 505.1528 (Cacl.: $[M+H]^+$ = 505.1522, error = 1.2 p.p.m.). (i) The total ion chromatography; (ii) The extracted ion chromatogram of the Lab derivative with a mass window between m/z 667.5-668.5. A peak with the retention time of 8.9 min was identified as the Lab derivative; (iii) The extracted ion chromatogram of the Hbt derivative with a mass window between m/z 504.5-505.5. A peak with the retention time of 7.3 min was identified as the Hbt derivative. The analysis by HPLC was on an ACQUITY UPLC C18 column (150 × 2.1 mm, 1.7 µm) by gradient elution of solvent A (H_2O with 0.1% formic acid) and solvent B (acetonitrile with 0.1% formic acid) with a flow rate of 0.2 mL/min over a 15 min period as follows: T = 0 min, 5% B; T = 2 min, 5% B; T = 12 min, 98% B; and T = 15 min, 98% B.

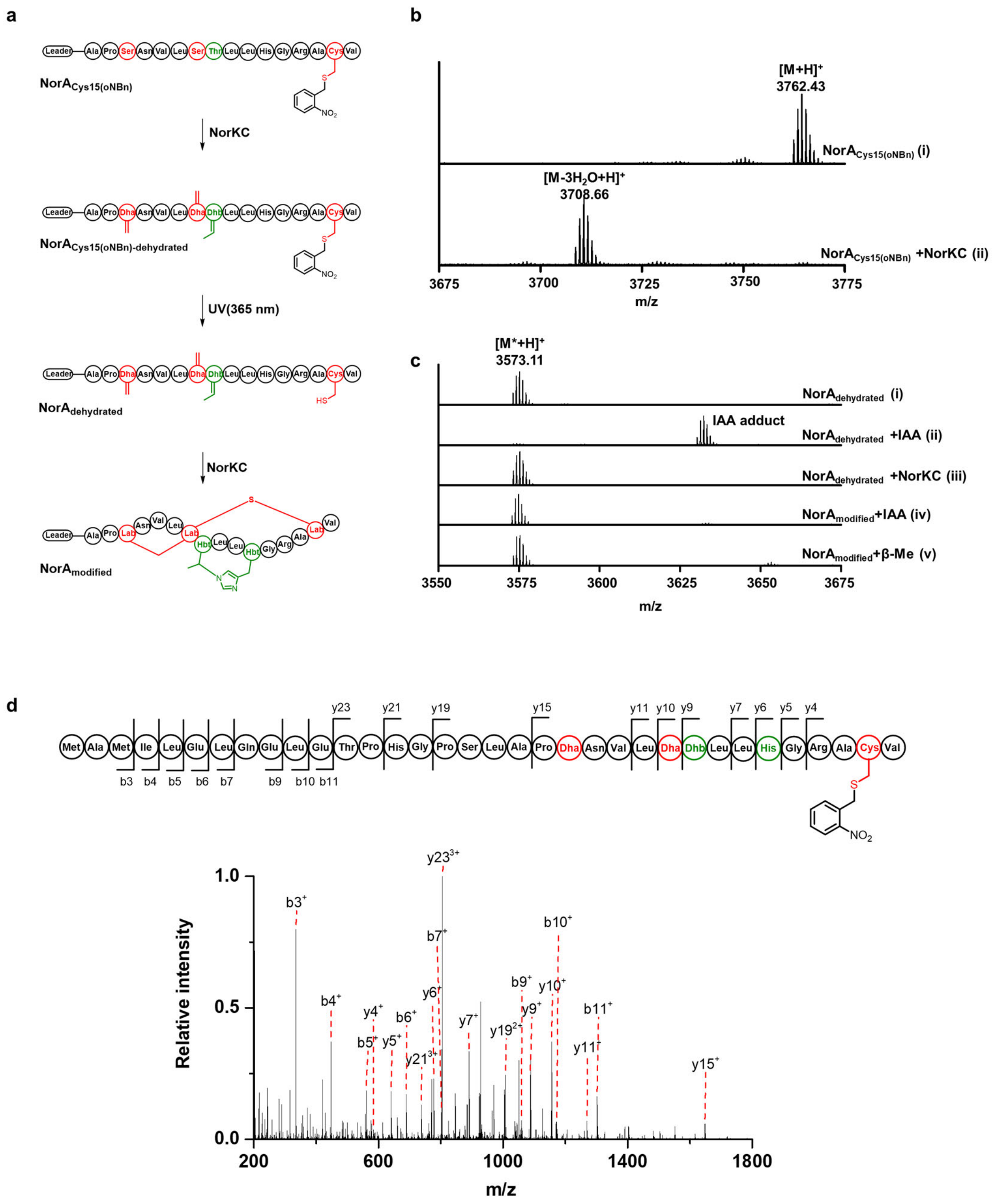

Species	Predicted Mass/Da	Observed Mass/Da	Error/ ppm	Species	Predicted Mass/Da	Observed Mass/Da	Error/ ppm
b3	334.1254	334.1262	2.4	y6	777.3461	777.3498	4.8
b4	447.2094	447.2105	2.5	y7	890.4302	890.4338	4.0
b5	560.2935	560.2955	3.6	y9	1086.5513	1086.5564	4.7
b6	689.3361	689.3381	2.9	y10	1155.5728	1155.5779	4.4
b7	802.4201	802.4228	3.4	y11	1268.6568	1268.6610	3.3
b9	1059.5213	1059.5249	3.4	y15	1647.8424	1647.8487	3.8
b10	1172.6054	1172.6096	3.6	$y19^{2+}$	1008.5278	1008.5290	1.2
b11	1301.6480	1301.6448	2.5	$y21^{3+}$	737.3811	737.3835	3.3
y4	583.2658	583.2679	3.6	$y23^{2+}$	1204.6182	1204.6125	4.7
y5	640.2872	640.2883	1.7				

Supplementary Figure 33. Sequential modification of $NorA_{Cys15(oNBn)}$ by NorKC. (a) Modification of $NorA_{Cys15(oNBn)}$ by NorKC. (b) MALDI-TOF-MS analysis of NorKC-modified $NorA_{Cys15(oNBn)}$ *in vitro*: (i) $NorA_{Cys15(oNBn)}$ (50 µM) in 20 mM Tris-HCl buffer (pH 8.0), 5 mM ATP, 1 mM $MgCl_2$, 0.1 mM TCEP. $NorA_{Cys15(oNBn)}$: $M_{obs.}$ =3762.43 Da, $M_{calc.}$ =3762.58 Da; (ii) $NorA_{Cys15(oNBn)}$ (50 µM) was incubated with NorKC (10 µM) at 28 °C for 1 h. Product $NorA_{Cys15(oNBn)\text{-}dehydrated}$: $M_{obs.}$ =3708.66 Da, $M_{calc.}$ =3708.89 Da; (c) MALDI-TOF-MS analysis of the modification of $NorA_{dehydrated}$ by NorKC *in vitro*: (i) $NorA_{Cys15(oNBn)\text{-}dehydrated}$ was irradiated by 365 nm light for 5 min to remove the protecting group. Product $NorA_{dehydrated}$:

$M_{obs.}$=3573.11 Da, $M_{calc.}$=3573.84 Da; (ii) NorA$_{dehydrated}$ was modified by IAA; (iii) NorA$_{dehydrated}$ was modified by NorKC (10 μM) at 28 °C for 1 h to generate NorA$_{modified}$; Incubation of NorA$_{modified}$ with IAA (iv) or βMe (v) did not result in any mass change. (d) LC-MS/MS analysis of NorA$_{Cys15(oNBn)-dehydrated}$. The *b* and *y* ions are listed in table and marked in the spectrum.

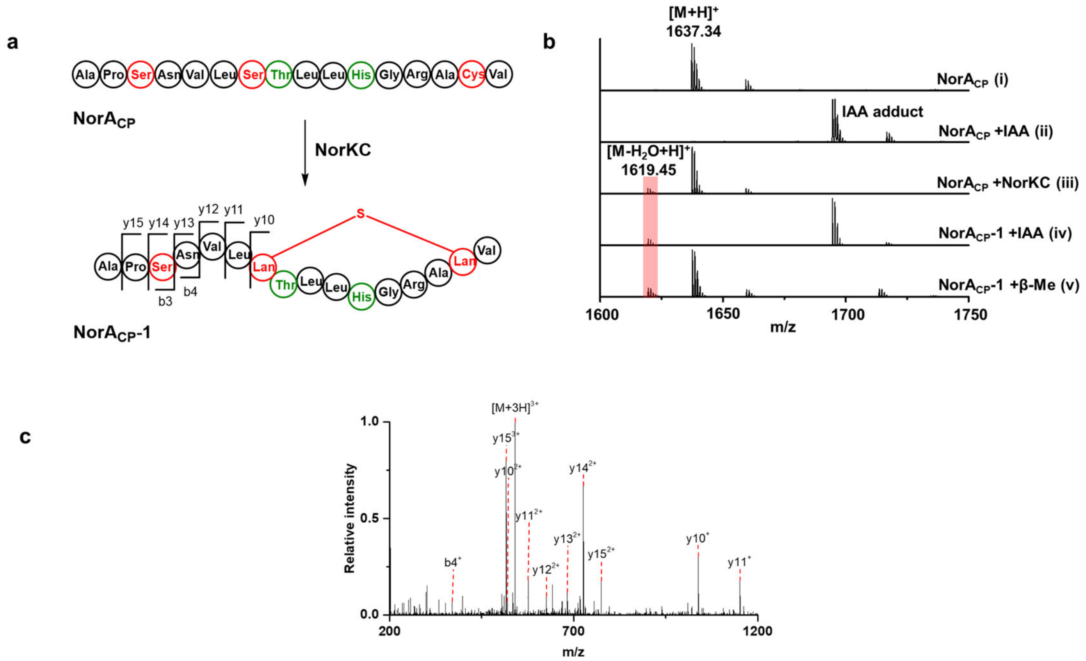

Species	Predicted Mass/Da	Observed Mass/Da	Error/ ppm	Species	Predicted Mass/Da	Observed Mass/Da	Error/ ppm
b4	370.1721	370.1733	3.2	y13^{2+}	682.8770	682.8747	3.4
y10	1038.5513	1038.5549	3.5	y14^{2+}	726.3930	726.3931	0.1
y10^{2+}	519.7793	519.7791	0.4	y15^{2+}	774.9194	774.9211	2.2
y11	1151.6353	1151.6361	0.7	y15^{3+}	516.9487	516.9491	0.8
y11^{2+}	576.3213	576.3204	1.6				

Supplementary Figure 34. Modification of NorA$_{CP}$ by NorKC is incomplete and inefficient. (a) and (b) NorKC only modified a very small portion of NorA$_{CP}$ by one-fold dehydration. Assay conditions: 20 mM Tris-HCl buffer (pH 8.0), 5 mM ATP, 1 mM MgCl$_2$, 0.1 mM TCEP, 50 µM NorA$_{CP}$ and 10 µM NorKC. NorA$_{CP}$: $M_{obs.}$ =1637.34 Da, $M_{calc.}$ =1637.88 Da; NorA$_{CP}$-1: $M_{obs.}$ =1619.87 Da, $M_{calc.}$ =1619.45 Da; (c) LC-MS/MS analysis of NorA$_{CP}$-1 indicated the formation of a Lan crosslink. The *b* and *y* ions are listed in table and marked in the spectrum.

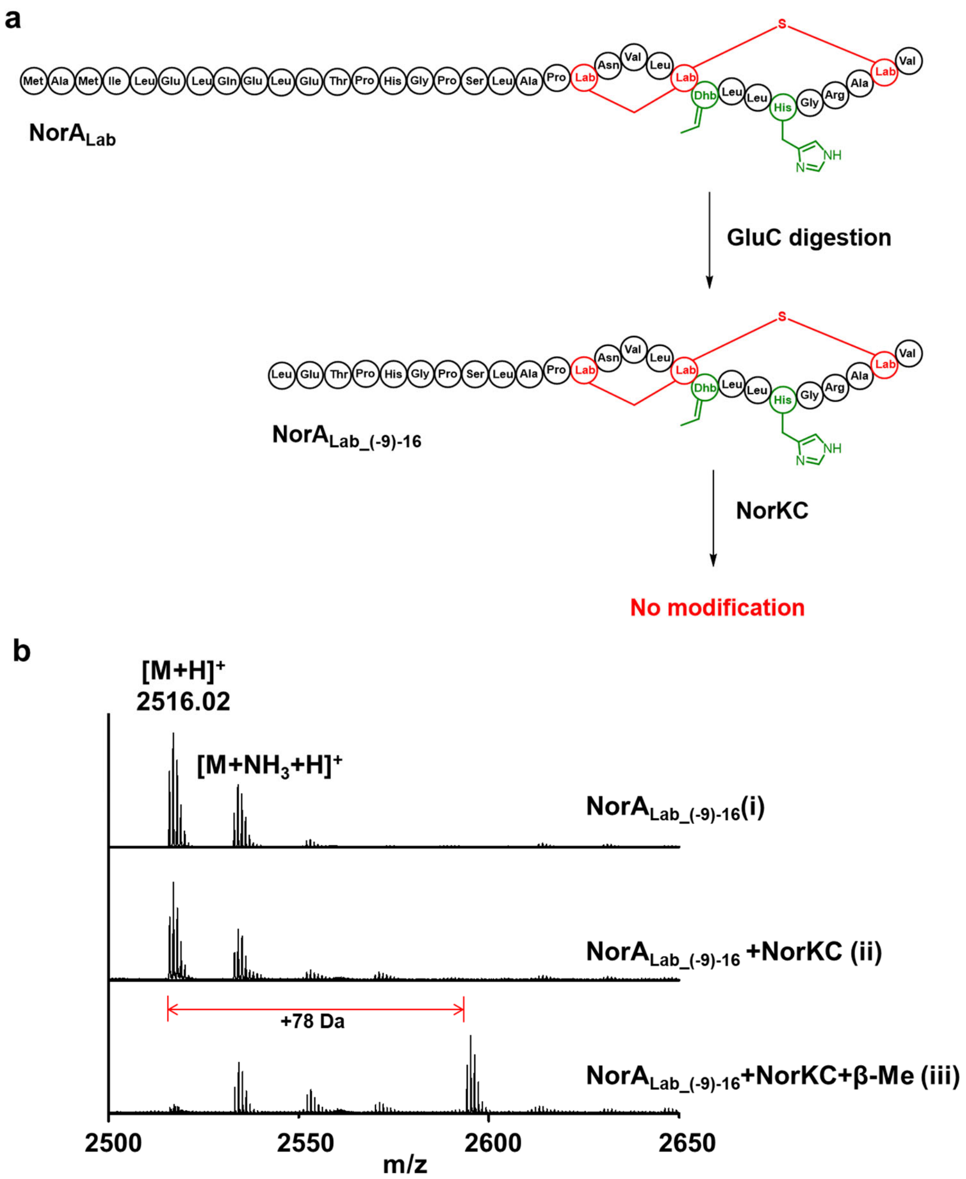

Supplementary Figure 35. The formation of the Hbt crosslink by NorKC requires the leader peptide. (a) NorA$_{Lab_(-9)-16}$ was not modified by NorKC; (b) βME fully converted NorKC-treated NorA$_{Lab_(-9)-16}$ peptide to the corresponding adduct *in vitro*, indicating that no Hbt was formed. Assay conditions: (i) NorA$_{Lab_(-9)-16}$ standard: $M_{obs.}$ =2516.02 Da, $M_{calc.}$ =2516.33 Da; (ii) NorA$_{Lab_(-9)-16}$ (50 µM) was incubated with NorKC (10 µM) in 20 mM Tris-HCl buffer (pH 8.0), 5 mM ATP, 1 mM $MgCl_2$, 0.1 mM TCEP at 28 °C for 1 h; (iii) The NorKC-treated NorA$_{Lab_(-9)-16}$ was incubated with 1 mM βME.

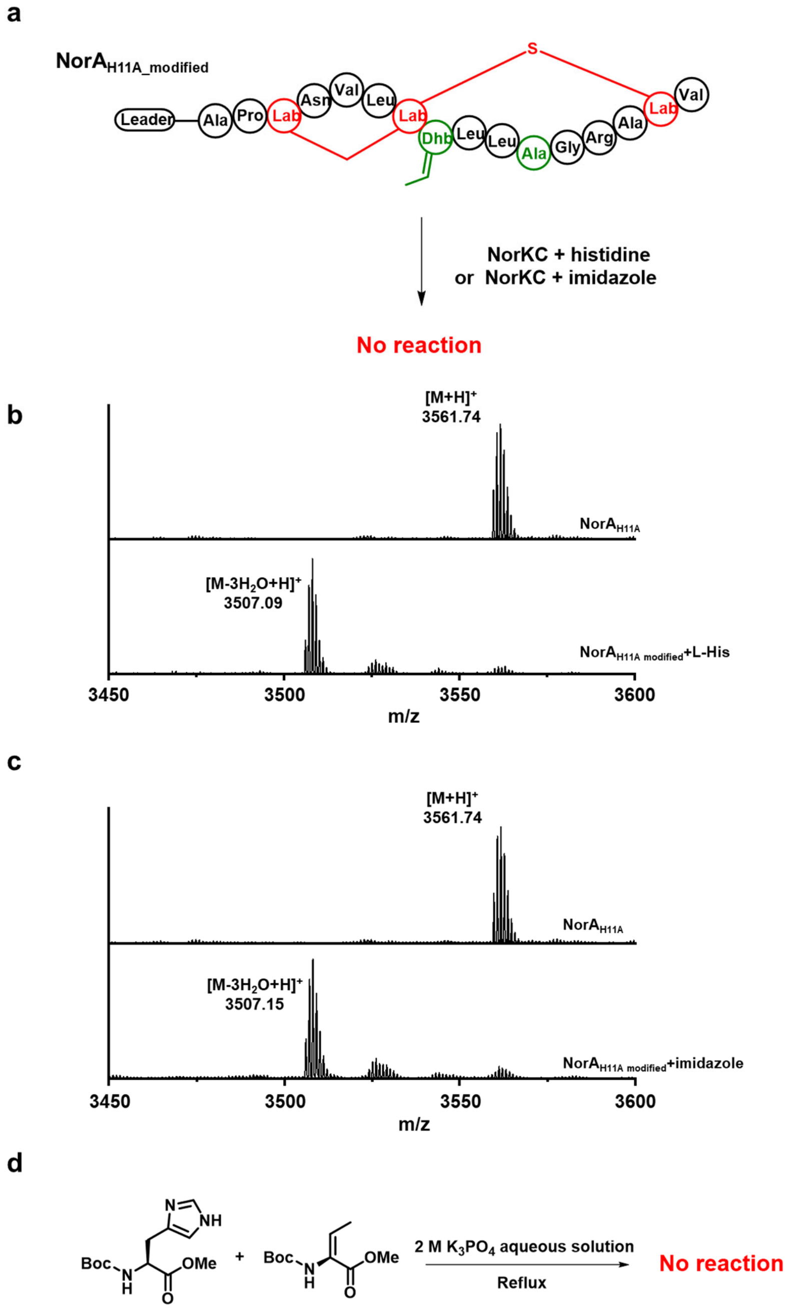

Supplementary Figure 36. Histidine or imidazole does not reactive with a Dhb derivative or the Dhb8 residue in NorA$_{H11A_modified}$. (a) Histidine or imidazole does not react with NorA$_{H11A_modified}$ in the presence of NorKC; (b) MALDI-TOF-MS analysis of the sample of NorA$_{H11A_modified}$ with histidine and NorKC *in vitro*. NorA$_{H11A_modified}$ (50 µM) was incubated with NorKC (10 µM) in 20 mM Tris-HCl buffer (pH 8.0), 5 mM ATP, 1 mM $MgCl_2$, 0.1 mM TCEP, 100 µM histidine at 28 °C for 1 h; (c) MALDI-TOF-MS analysis of the sample of NorA$_{H11A_modified}$ with imidazole and NorKC *in vitro*. NorA$_{H11A_modified}$ (50 µM) was incubated with NorKC (10 µM) in 20 mM Tris-HCl buffer (pH 8.0), 5 mM ATP, 1 mM $MgCl_2$, 0.1 mM TCEP, 100 µM imidazole at 28 °C for 1 h; NorA$_{H11A}$: $M_{obs.}$ =3561.74 Da, $M_{calc.}$ =3561.84 Da; NorA$_{H11A_modified}$: $M_{obs.}$ =3507.15 Da, $M_{calc.}$ =3507.81 Da. (d). Histidine does not react with a Dhb derivative in alkaline aqueous conditions by reflux. A solution of Dhb derivative (17.9 mg, 0.084 mmol) and Boc-His-OMe (29.6 mg, 0.11 mmol) in 0.2 M K_3PO_4 solution (1 mL) was refluxed overnight in a 10 mL schlenk tube. The formation of a His-Dhb adduct was not observed.

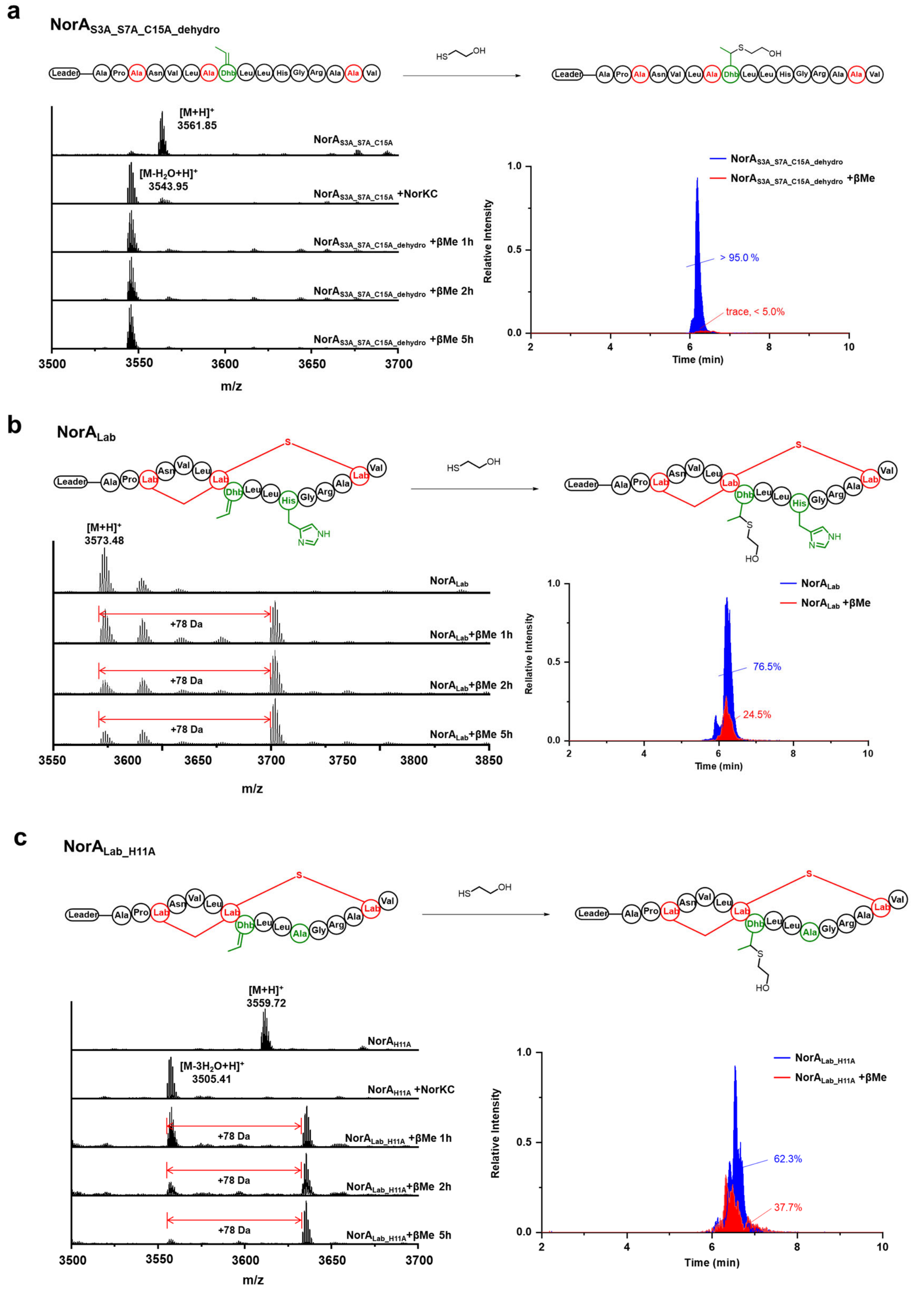

Supplementary Figure 37. The formation of a Lab ring in NorA increases the reactivity of Dhb8. (a. Left) MALDI-TOF-MS analysis of $\text{NorA}_{\text{S3A\_S7A\_C15A\_dehydro}}$ incubated with 1 mM βME for 1 h, 2 h and 5 h. $\text{NorA}_{\text{S3A\_S7A\_C15A}}$: $M_{\text{obs.}}$ =3561.85 Da, $M_{\text{calc.}}$ =3561.56 Da; $\text{NorA}_{\text{S3A\_S7A\_C15A\_dehydro}}$: $M_{\text{obs.}}$ =3543.95 Da, $M_{\text{calc.}}$ =3543.55 Da; (a. Right) LC-MS analysis of the reaction of $\text{NorA}_{\text{S3A\_S7A\_C15A\_dehydro}}$ with 1 mM βME for 1 h. The signal of $\text{NorA}_{\text{S3A\_S7A\_C15A\_dehydro}}$ was labeled by blue, and the signal of its βME adduct was labeled by red. (b. Left) MALDI-TOF-MS analysis of NorA_{Lab} incubated with 1 mM βME for 1 h, 2 h

and 5 h. $NorA_{Lab}$: $M_{obs.}$ =3573.48 Da, $M_{calc.}$ =3573.74 Da; (b. Right) LC-MS analysis of the reaction of $NorA_{Lab}$ with 1 mM βME for 1 h. The signal of $NorA_{Lab}$ was labeled by blue, and the signal of its βME adduct was labeled by red. (c. Left) MALDI-TOF-MS analysis of the reaction of $NorA_{H11A\text{-}Lab}$ with 1 mM βME for 1 h, 2 h and 5 h. $NorA_{H11A}$: $M_{obs.}$ =3559.72 Da, $M_{calc.}$ =3559.53 Da; $NorA_{H11A\_Lab}$: $M_{obs.}$ =3505.41 Da, $M_{calc.}$ =3505.53 Da; (c. Right) LC-MS analysis of the reaction of $NorA_{H11A\_Lab}$ with 1 mM βME for 1 h. The signal of $NorA_{H11A\_Lab}$ was labeled by blue, and the signal of its βME-adduct was labeled by red.

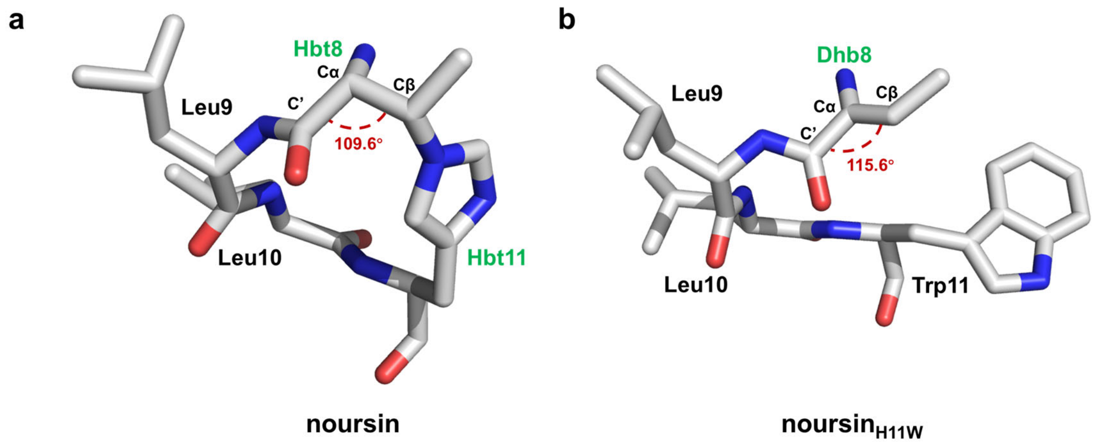

Supplementary Figure 38. The C'-Cα-Cβ bond angle of Hbt8 residue and Dhb8 in NMR structures of noursin (a) and $noursin_{H11W}$ (b), respectively. Similar to the NMR structure of noursin, three-dimensional conformations of $noursin_{H11W}$ were modeled using ^{1}H-^{1}H distance constraints derived from 2D ^{1}H-^{1}H NOESY spectra. The Xplor-NIH software package was used for the structure calculation with the configuration of the Lab crosslink set as *3S*,*7S*,*15R*. The PDB file of the NMR 3D structure of $noursin_{H11W}$ is contained in the .zip file named SI_data.zip.

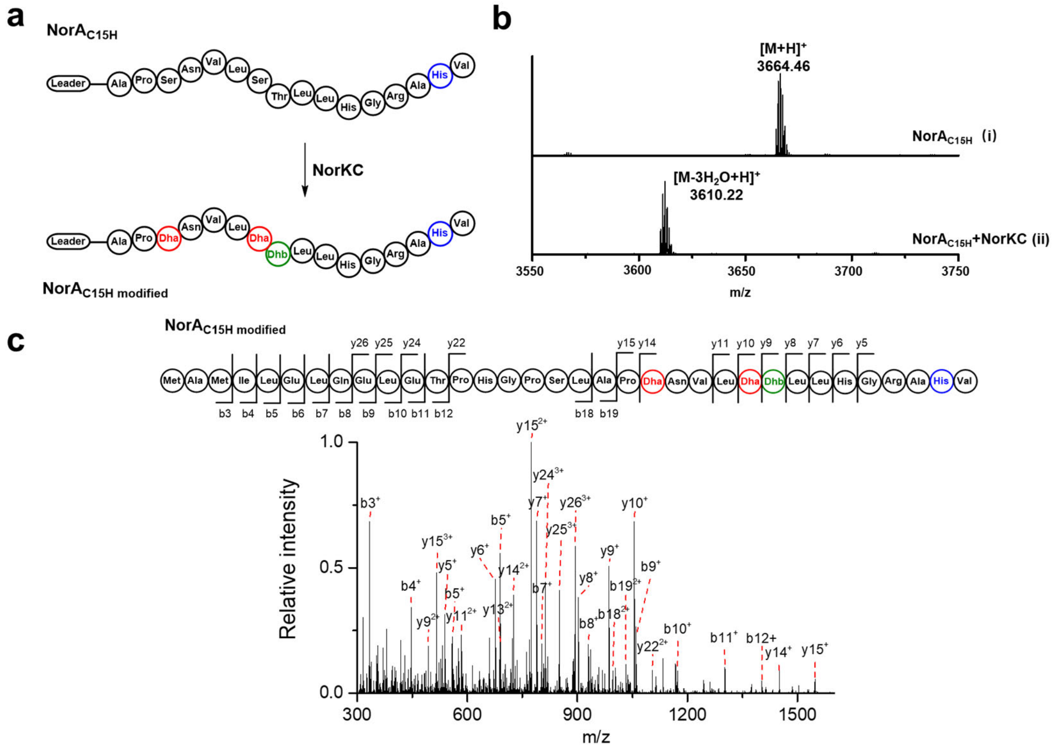

Species	Predicted Mass/Da	Observed Mass/Da	Error/ ppm	Species	Predicted Mass/Da	Observed Mass/Da	Error/ ppm
b3	334.1254	334.1256	0.6	y8	902.5319	902.5301	2.0
b4	447.2094	447.2087	1.6	y9	985.5690	985.5682	0.8
b5	560.2935	560.2907	5.0	y10	1054.5904	1054.5909	0.5
b6	689.3361	689.3364	0.4	y11	1167.6745	1167.6750	0.4
b7	802.4201	802.4185	2.0	y14	1449.8072	1449.8044	1.9
b8	930.4787	930.4813	2.8	y15	1546.8600	1546.8617	1.1
b9	1059.5213	1059.5218	0.5	$y9^{2+}$	493.2881	493.2874	1.4
b10	1172.6054	1172.6004	4.3	$y13^{2+}$	690.8965	690.8951	2.0
b11	1301.6480	1301.6530	3.8	$y14^{2+}$	725.4073	725.4074	0.1
b12	1402.6956	1402.6934	1.6	$y15^{2+}$	773.9336	773.9339	0.4
$b18^{2+}$	996.0025	996.0007	1.8	$y15^{3+}$	516.2915	516.2919	0.8
$b19^{2+}$	1031.5210	1031.5227	1.6	$y22^{2+}$	1103.6032	1103.6088	5.1
y5	539.3049	539.3041	1.5	$y24^{3+}$	812.7680	812.7683	0.4
y6	676.3638	676.3634	0.6	$y25^{3+}$	850.4627	850.4646	2.2
y7	789.4478	789.4464	1.8	$y26^{3+}$	893.4769	893.4772	0.3

Supplementary Figure 39. Modification of NorA$_{C15H}$ by NorKC. (a) Modification of NorA$_{C15H}$ by NorKC. (b) MALDI-TOF-MS analysis of NorKC-modified NorA$_{C15H}$ *in vitro*: (i) 20 mM Tris-HCl buffer (pH 8.0), 5 mM ATP, 10 mM $MgCl_2$, 0.1 mM TCEP, 50 µM NorA$_{C15H}$, 10 µM NorKC. NorA$_{C15H}$: $M_{obs.}$ =3664.46 Da, $M_{calc.}$ =3661.92 Da; (ii) NorA$_{C15H}$ incubated with NorKC (10 µM) at 28 °C for 1 h. Product NorA$_{C15H\ modified}$: $M_{obs.}$ =3610.22 Da, $M_{calc.}$ =3607.89 Da; (c) LC-MS/MS analysis of NorA$_{C15H\ modified}$. The *b* and *y* ions are listed in table and marked in the spectrum.

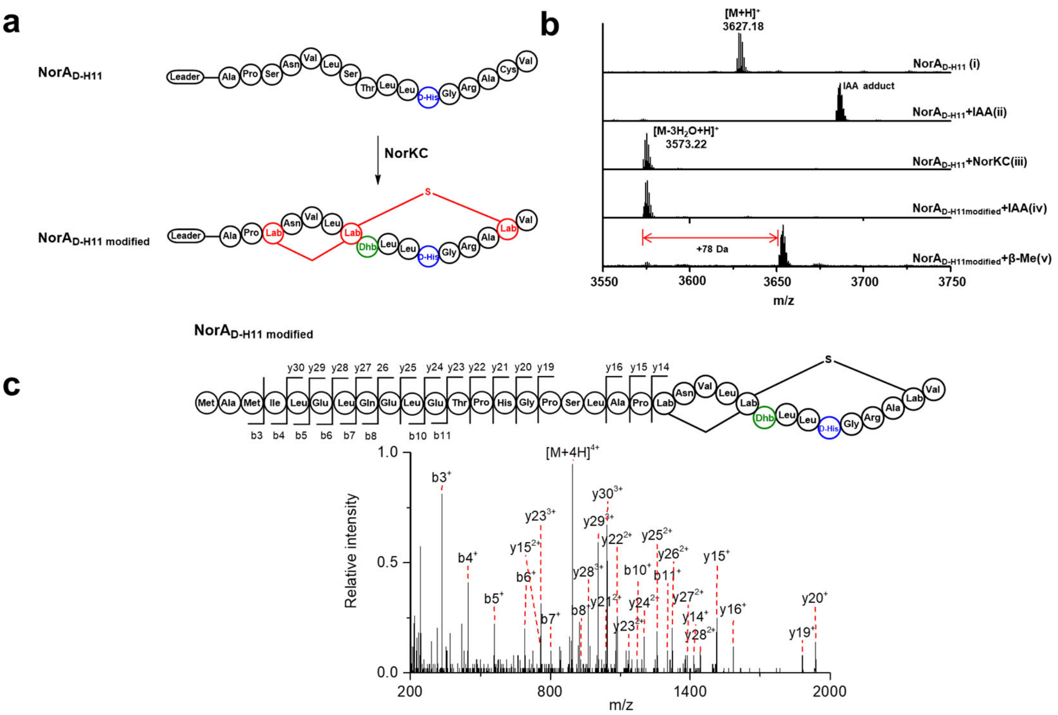

Species	Predicted Mass/Da	Observed Mass/Da	Error/ ppm	Species	Predicted Mass/Da	Observed Mass/Da	Error/ ppm
b3	334.1254	334.1247	2.1	y20	1938.0377	1938.0293	4.3
b4	447.2094	447.2085	2.0	$y21^{2+}$	1038.0519	1038.0515	0.4
b5	560.2935	560.2915	3.6	$y22^{2+}$	1086.5783	1086.5769	1.3
b6	689.3361	689.3358	0.4	$y23^{2+}$	1137.1022	1137.1043	1.8
b7	802.4201	802.4238	4.6	$y23^{3+}$	758.4039	758.4032	0.9
b8	930.4787	930.4744	4.6	$y24^{2+}$	1201.6235	1201.6188	3.9
b10	1172.6054	1172.5998	4.8	$y25^{2+}$	1258.1655	1258.1598	4.5
b11	1301.6480	1301.6442	2.9	$y26^{2+}$	1322.6868	1322.6804	4.8
y14	1415.7575	1415.7528	3.3	$y27^{2+}$	1386.7161	1386.7171	0.7
y15	1512.8103	1512.8121	1.2	$y28^{2+}$	1443.2581	1443.2607	1.8
$y15^{2+}$	756.9088	756.9058	4.0	$y28^{3+}$	962.5078	962.5087	0.9
y16	1583.8474	1583.8462	0.8	$y29^{3+}$	1005.5221	1005.5217	0.4
y19	1881.0162	1881.0256	5.0	$y30^{3+}$	1043.2167	1043.2178	1.1

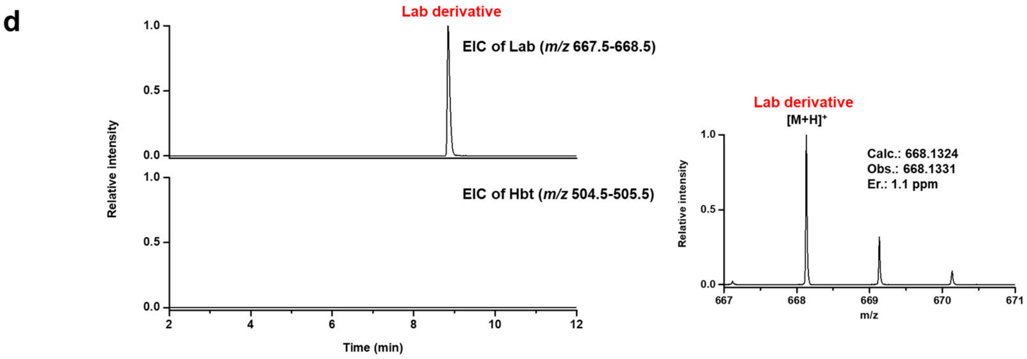

Supplementary Figure 40. Modification of NorA$_{D\text{-}H11}$ by NorKC. (a) Modification of NorA$_{D\text{-}H11}$ by NorKC. (b) MALDI-TOF-MS analysis of the modification of NorA$_{D\text{-}H11}$ by NorKC *in vitro*: (i) 20 mM Tris-HCl buffer (pH 8.0), 5 mM ATP, 10 mM $MgCl_2$, 0.1 mM TCEP, 50 µM NorA$_{D\text{-}H11}$, 10 µM NorKC. NorA$_{D\text{-}H11}$: $M_{calc.}$ =3627.86 Da, $M_{obs.}$ =3627.18 Da. (ii) NorA$_{D\text{-}H11}$ treated by IAA led to the formation of IAA adduct; (iii) NorA$_{D\text{-}H11}$ incubated with NorKC (10 µM) at 28 °C for 1 h. NorA$_{D\text{-}H11\ modified}$: $M_{calc.}$ =3573.82 Da, $M_{obs.}$ =3573.22 Da; (iv) NorA$_{D\text{-}H11\ modified}$ treated by IAA did not result in any mass change; (v) NorA$_{D\text{-}H11\ modified}$ treated by β-Me led to the formation of β-Me adduct. (c) LC-MS/MS analysis of NorA$_{D\text{-}H11\ modified}$. The *b* and *y* ions are listed in table and marked in the spectrum. (d) LC-MS analysis of

the derivatized hydrolysate of $NorA_{D\text{-}H11\ modified}$, and HRMS analysis of the Lab derivative: $[M+H]^+$ = 668.1331 (Cacl.: $[M+H]^+$ = 668.1324, error = 1.1 p.p.m.). The extracted ion chromatogram of the Lab derivative with a mass window between m/z 667.5-668.5 Da. A peak with the retention time of 8.9 min was identified as the Lab derivative. The analysis by HPLC was on an ACQUITY UPLC C18 column (150 × 2.1 mm, 1.7 µm) by gradient elution of solvent A (H_2O with 0.1% formic acid) and solvent B (acetonitrile with 0.1% formic acid) with a flow rate of 0.2 mL/min over a 15 min period as follows: T = 0 min, 5% B; T = 2 min, 5% B; T = 12 min, 98% B; and T = 15 min, 98% B.

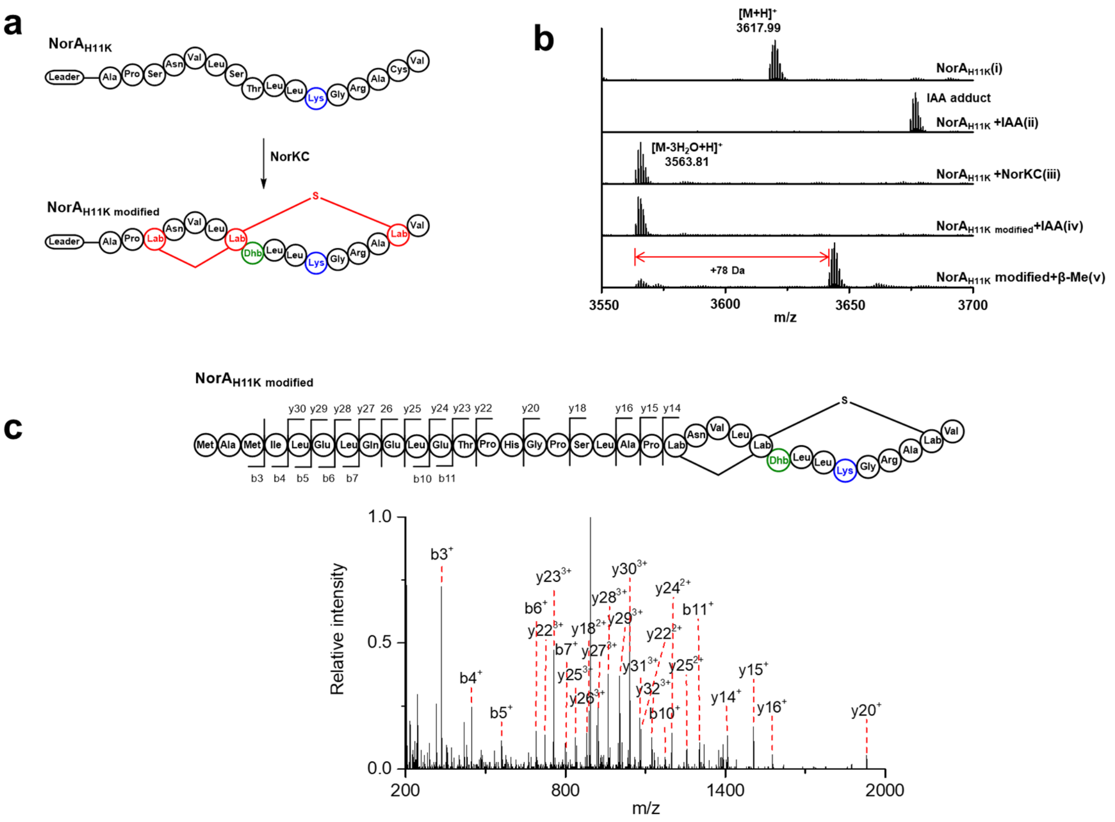

Species	Predicted Mass/Da	Observed Mass/Da	Error/ ppm	Species	Predicted Mass/Da	Observed Mass/Da	Error/ ppm
b3	334.1254	334.1250	1.2	y22^{2+}	1082.0964	1082.1007	4.0
b4	447.2094	447.2090	0.9	y22^{3+}	721.7333	721.7298	4.8
b5	560.2935	560.2925	1.8	y23^{3+}	755.4159	755.4150	1.2
b6	689.3361	689.3363	0.3	y24^{2+}	1197.1415	1197.1425	0.8
b7	802.4201	802.4165	4.5	y25^{2+}	1253.6835	1253.6797	3.0
b10	1172.6054	1172.5989	5.5	y25^{3+}	836.1248	836.1224	2.9
b11	1301.6480	1301.6506	2.0	y26^{3+}	879.1390	879.1448	6.6
y14	1406.7936	1406.7868	4.8	y27^{3+}	921.8252	921.8239	1.4
y15	1503.8463	1503.8501	2.5	y28^{3+}	959.5198	959.5160	4.0
y16	1574.8834	1574.8843	0.6	y29^{3+}	1002.5340	1002.5378	3.8
y18^{2+}	888.0034	888.0074	4.5	y30^{3+}	1040.2287	1040.2225	6.0
y20	1929.0738	1929.0833	4.9				

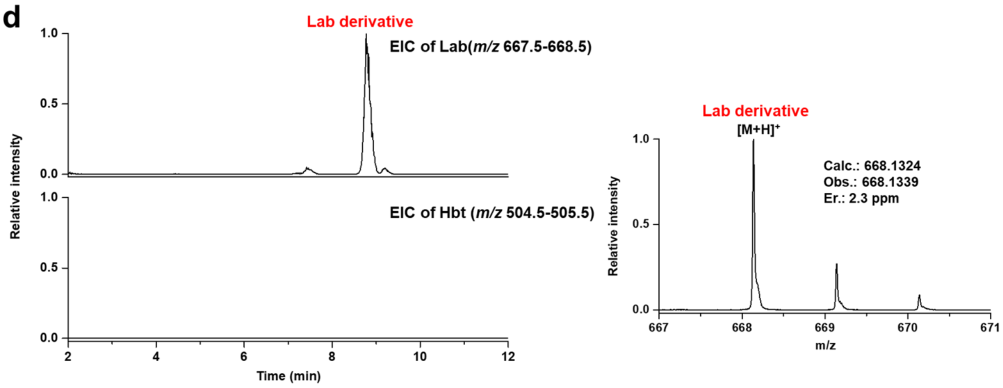

Supplementary Figure 41. Modification of NorA$_{H11K}$ by NorKC. (a) Modification of NorA$_{H11K}$ by NorKC. (b) MALDI-TOF-MS analysis of the modification of NorA$_{H11K}$ by NorKC *in vitro*: (i) 20 mM Tris-HClbuffer (pH 8.0), 5 mM ATP, 10 mM $MgCl_2$, 0.1 mM TCEP, 50 µM NorA$_{H11K}$, 10 µM NorKC. NorA$_{H11K}$: $M_{calc.}$ =3618.90 Da, $M_{obs.}$ =3617.99 Da. (ii) NorA$_{H11K}$ treated by IAA led to the formation of IAA adduct; (iii) NorA$_{H11K}$ incubated with NorKC (10 µM) at 28 °C for 1 h. NorA$_{H11K\ modified}$: $M_{calc.}$ =3564.87 Da, $M_{obs.}$ =3563.81 Da; (iv) NorA$_{H11K\ modified}$ treated by IAA did not result in any mass change; (v) NorA$_{H11K\ modified}$ treated by β-Me led to the formation of β-Me adduct. (c) LC-MS/MS analysis of

$NorA_{H11K\ modified}$. The *b* and *y* ions are listed in table and marked in the spectrum. (d) LC-MS analysis of the derivatized hydrolysate of $NorA_{H11K\ modified}$, and HRMS analysis of the Lab derivative: $[M+H]^+$ = 668.1339 (Cacl.: $[M+H]^+$ = 668.1324, error = 2.3 p.p.m.). The extracted ion chromatogram of the Lab derivative with a mass window between m/z 667.5-668.5 Da. A peak with the retention time of 8.8 min was identified as the Lab derivative. The analysis by HPLC was on an ACQUITY UPLC C18 column (150 × 2.1 mm, 1.7 μm) by gradient elution of solvent A (H_2O with 0.1% formic acid) and solvent B (acetonitrile with 0.1% formic acid) with a flow rate of 0.2 mL/min over a 15 min period as follows: T = 0 min, 5% B; T = 2 min, 5% B; T = 12 min, 98% B; and T = 15 min, 98% B.

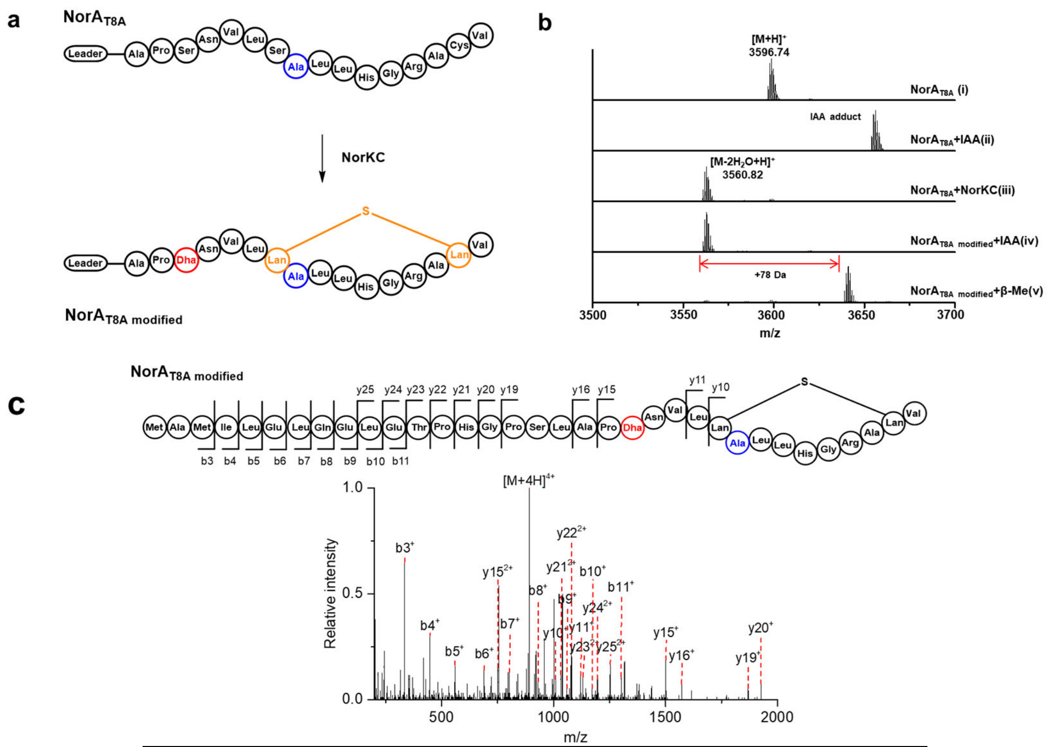

Species	Predicted Mass/Da	Observed Mass/Da	Error/ ppm	Species	Predicted Mass/Da	Observed Mass/Da	Error/ ppm
b3	334.1254	334.1250	1.2	y15	1500.8103	1514.7991	7.5
b4	447.2094	447.2071	5.1	y15^{2+}	750.9088	750.9095	0.9
b5	560.2935	560.2909	4.6	y16	1571.8474	1571.8498	1.5
b6	689.3361	689.3368	1.0	y19	1869.0163	1869.0044	6.4
b7	802.4201	802.4136	8.1	y20	1926.0377	1926.0298	4.1
b8	930.4787	930.4777	1.1	y21^{2+}	1032.0520	1032.0581	5.9
b9	1059.5213	1059.5156	5.4	y22^{2+}	1080.5783	1080.5777	0.6
b10	1172.6054	1172.5977	6.6	y23^{2+}	1131.1022	1131.1023	0.1
b11	1301.6480	1301.6445	2.7	y24^{2+}	1195.6235	1195.6208	2.3
y10	1008.5407	1008.5437	2.9	y25^{2+}	1252.1655	1252.1642	1.0
y11	1121.6248	1121.6215	2.9				

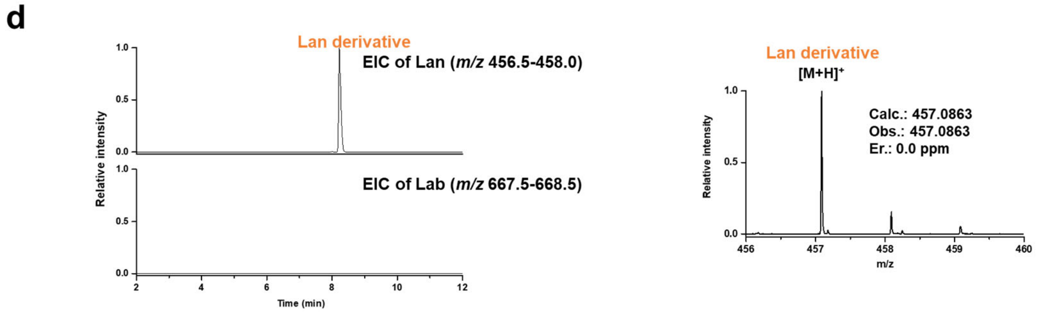

Supplementary Figure 42. Modification of NorA$_{T8A}$ by NorKC. (a) Modification of NorA$_{T8A}$ by NorKC. (b) MALDI-TOF-MS analysis of the modification of NorA$_{T8A}$ by NorKC *in vitro*: (i) 20 mM Tris-HCl buffer (pH 8.0), 5 mM ATP, 10 mM $MgCl_2$, 0.1 mM TCEP, 50 µM NorA$_{T8A}$, 10 µM NorKC. NorA$_{T8A}$: $M_{calc.}$ =3597.85 Da, $M_{obs.}$ =3596.74 Da. (ii) NorA$_{T8A}$ treated by IAA led to the formation of IAA adduct; (iii) NorA$_{T8A}$ incubated with NorKC (10 µM) at 28 °C for 1 h. NorA$_{T8A\ modified}$: $M_{calc.}$ =3561.83 Da, $M_{obs.}$ =3560.82 Da; (iv) NorA$_{T8A\ modified}$ treated by IAA did not result in any mass change; (v) NorA$_{T8A\ modified}$ treated by β-Me led to the formation of β-Me adduct. (c) LC-MS/MS analysis of NorA$_{T8A\ modified}$. The *b* and *y* ions are listed in table and marked in the spectrum. (d) LC-MS analysis of the derivatized hydrolysate of NorA$_{T8A\ modified}$, and HRMS analysis of the Lan derivative: $[M+H]^+$ = 457.0863 (Cacl.: $[M+H]^+$ = 457.0863, error = 0.0 p.p.m.). The extracted ion chromatogram of the Lan derivative with a mass window between m/z 456.5-458.0 Da. A peak with the retention time of 8.2 min was identified as

the Lan derivative. The analysis by HPLC was on an ACQUITY UPLC C18 column (150 × 2.1 mm, 1.7 μm) by gradient elution of solvent A (H_2O with 0.1% formic acid) and solvent B (acetonitrile with 0.1% formic acid) with a flow rate of 0.2 mL/min over a 15 min period as follows: T = 0 min, 5% B; T = 2 min, 5% B; T = 12 min, 98% B; and T = 15 min, 98% B.

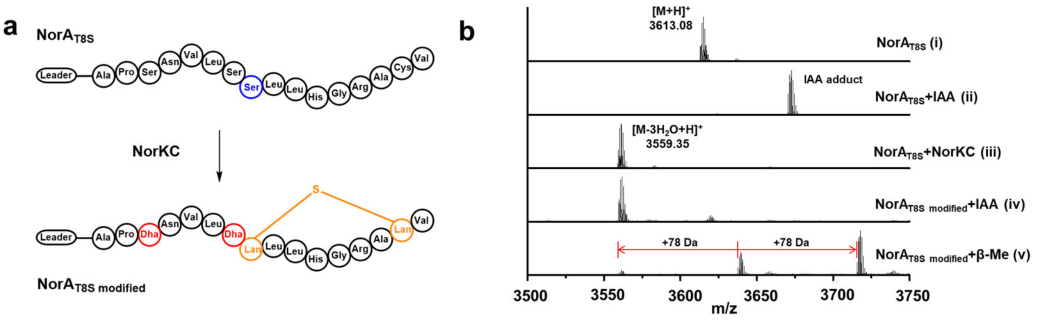

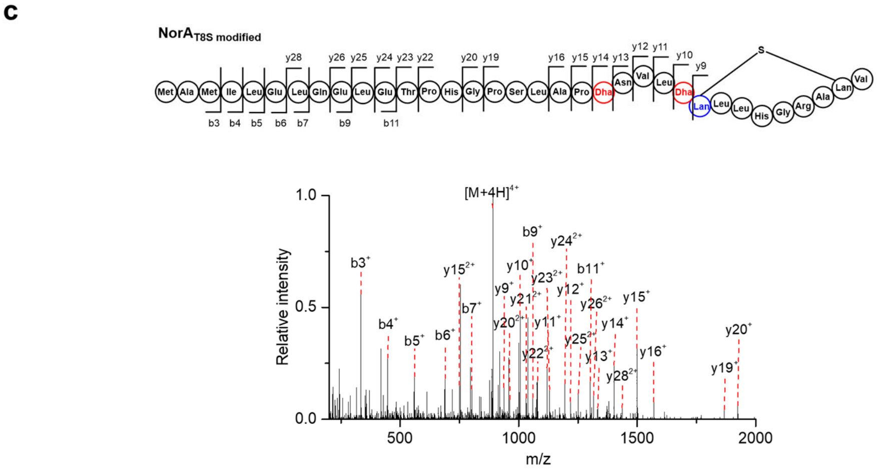

Species	Predicted Mass/Da	Observed Mass/Da	Error/ ppm	Species	Predicted Mass/Da	Observed Mass/Da	Error/ ppm
b3	334.1254	334.1256	0.6	y15	1498.7946	1498.7905	2.7
b4	447.2094	447.2084	2.2	y16	1569.8317	1569.8298	1.2
b5	560.2935	560.2936	0.2	y19	1867.0006	1866.9834	9.2
b6	689.3361	689.3370	1.3	y20	1924.0221	1924.0221	0.0
b7	802.4201	802.4193	1.0	$y20^{2+}$	962.5147	962.5143	0.4
b9	1059.5213	1059.5186	2.5	$y21^{2+}$	1031.0441	1031.0402	3.8
b11	1301.6480	1301.6452	2.2	$y22^{2+}$	1079.5705	1079.5739	3.1
y9	937.5036	937.5054	1.9	$y23^{2+}$	1130.0943	1130.0933	0.9
y10	1006.5250	1006.5235	1.5	$y24^{2+}$	1194.6156	1194.6096	5.0
y11	1119.6091	1119.6074	1.5	$y25^{2+}$	1251.1577	1251.1598	1.7
y12	1218.6775	1218.6715	4.9	$y26^{2+}$	1315.6790	1315.6763	2.1
y13	1332.7204	1332.7169	2.6	$y28^{2+}$	1436.2503	1436.2544	2.9
y14	1401.7419	1401.7388	2.2				

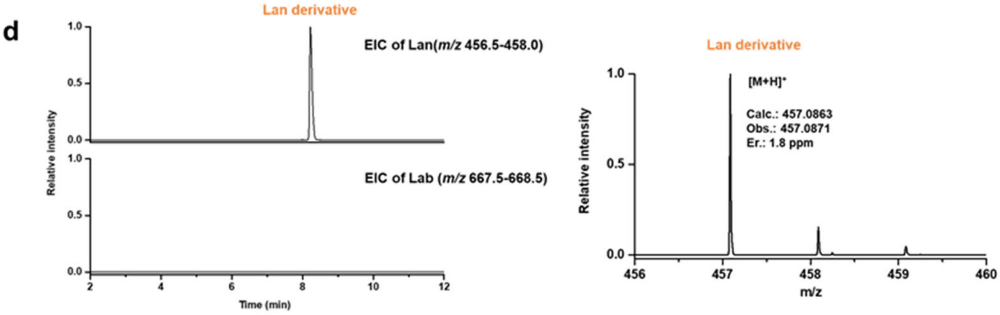

Supplementary Figure 43. Modification of NorA$_{T8S}$ by NorKC. (a) Modification of NorA$_{T8S}$ by NorKC. (b) MALDI-TOF-MS analysis of the modification of NorA$_{T8S}$ by NorKC *in vitro*: (i) 20 mM Tris-HCl buffer (pH 8.0), 5 mM ATP, 10 mM $MgCl_2$, 0.1 mM TCEP, 50 µM NorA$_{T8S}$, 10 µM NorKC. NorA$_{T8S}$: $M_{calc.}$ =3613.85 Da, $M_{obs.}$ =3613.08 Da. (ii) NorA$_{T8S}$ treated by IAA led to the formation of IAA adduct; (iii) NorA$_{T8S}$ incubated with NorKC (10 µM) at 28 °C for 1 h. NorA$_{T8S\ modified}$: $M_{calc.}$ =3559.82 Da, $M_{obs.}$ =3559.35 Da; (iv) NorA$_{T8S\ modified}$ treated by IAA did not result in any mass change; (v) NorA$_{T8S\ modified}$

treated by β-Me led to the formation of 2-fold β-Me adduct. (c) LC-MS/MS analysis of $NorA_{T8S\ modified}$. The *b* and *y* ions are listed in table and marked in the spectrum. (d) LC-MS analysis of the derivatized hydrolysate of $NorA_{T8S\ modified}$, and HRMS analysis of the Lan derivative: $[M+H]^+$ = 457.0871 (Cacl.: $[M+H]^+$ = 457.0863, error = 1.8 p.p.m.). The extracted ion chromatogram of the Lan derivative with a mass window between m/z 456.5-458.0 Da. A peak with the retention time of 8.2 min was identified as the Lan derivative. The analysis by HPLC was on an ACQUITY UPLC C18 column (150 × 2.1 mm, 1.7 µm) by gradient elution of solvent A (H_2O with 0.1% formic acid) and solvent B (acetonitrile with 0.1% formic acid) with a flow rate of 0.2 mL/min over a 15 min period as follows: T = 0 min, 5% B; T = 2 min, 5% B; T = 12 min, 98% B; and T = 15 min, 98% B.

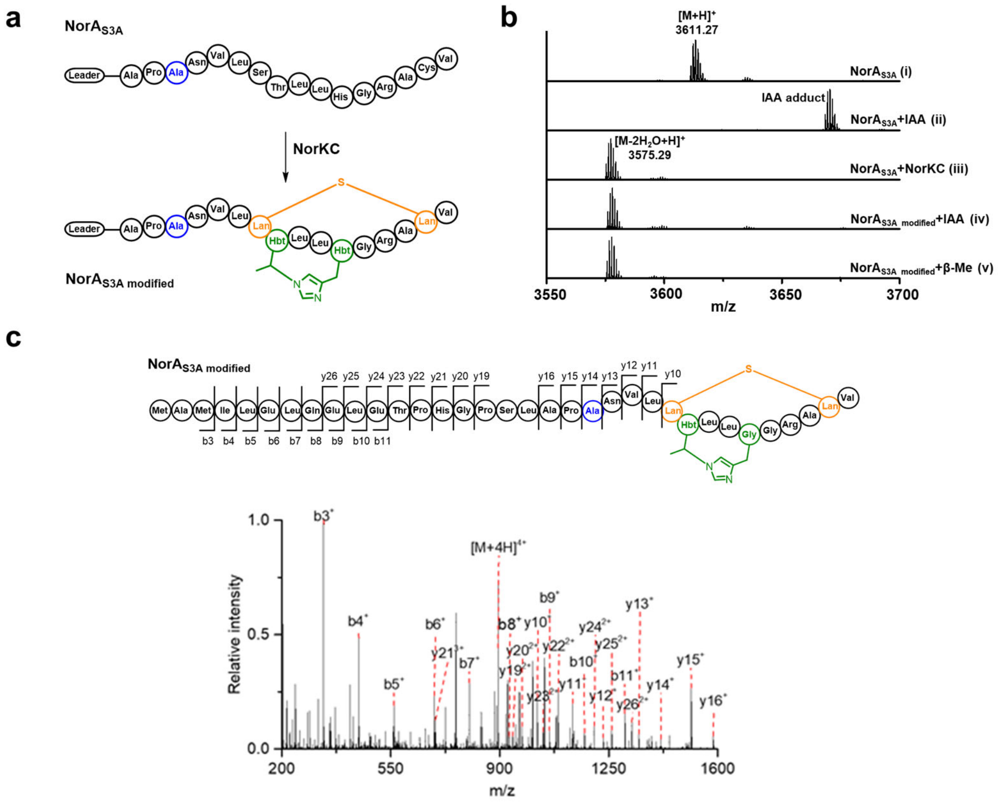

Species	Predicted Mass/Da	Observed Mass/Da	Error/ ppm	Species	Predicted Mass/Da	Observed Mass/Da	Error/ ppm
b3	334.1254	334.1258	1.2	y13	1346.7361	1346.7360	0.1
b4	447.2094	447.2100	1.3	y14	1417.7732	1417.7719	0.9
b5	560.2935	560.2945	1.8	y15	1514.8260	1514.8276	1.1
b6	689.3361	689.3377	2.3	y16	1585.8631	1585.8647	1.0
b7	802.4201	802.4256	6.9	$y19^{2+}$	942.0196	942.0205	1.0
b8	930.4787	930.4807	2.1	$y20^{2+}$	970.5303	970.5348	4.6
b9	1059.5213	1059.5246	3.1	$y21^{3+}$	693.0423	693.0474	7.4
b10	1172.6054	1172.6061	0.6	$y22^{2+}$	1087.5862	1087.5891	2.7
b11	1301.6480	1301.6526	3.5	$y23^{2+}$	1138.1100	1138.1142	3.7
y10	1020.5407	1020.5430	2.3	$y24^{2+}$	1202.6313	1202.6353	3.3
y11	1133.6247	1133.6272	2.2	$y25^{2+}$	1259.1733	1259.1783	4.0
y12	1232.6932	1232.6944	1.0	$y26^{2+}$	1323.6946	1323.6954	0.6

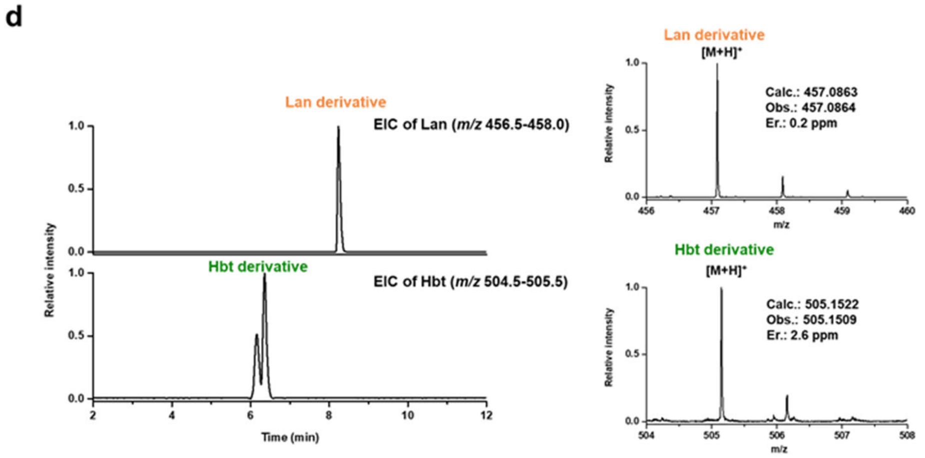

Supplementary Figure 44. Modification of NorA$_{S3A}$ by NorKC. (a) Modification of NorA$_{S3A}$ by NorKC. (b) MALDI-TOF-MS analysis of the modification of NorA$_{S3A}$ by NorKC *in vitro*: (i) 20 mM Tris-HCl buffer (pH 8.0), 5 mM ATP, 10 mM $MgCl_2$, 0.1 mM TCEP, 50 µM NorA$_{S3A}$, 10 µM NorKC. NorA$_{S3A}$: $M_{calc.}$ =3611.87 Da, $M_{obs.}$ =3611.27 Da. (ii) NorA$_{S3A}$ treated by IAA led to the formation of IAA adduct; (iii) NorA$_{S3A}$ incubated with NorKC (10 µM) at 28 °C for 1 h. NorA$_{S3A\ modified}$: $M_{calc.}$ =3575.85 Da, $M_{obs.}$

=3575.29 Da; (iv) $NorA_{S3A\ modified}$ treated by IAA did not result in any mass change; (v) $NorA_{S3A\ modified}$ treated by βME did not result in any mass change. (c) LC-MS/MS analysis of $NorA_{S3A\ modified}$. The *b* and *y* ions are listed in table and marked in the spectrum. (d) LC-MS analysis of the derivatized hydrolysate of $NorA_{S3A\ modified}$, and HRMS analysis of the Lan derivative: $[M+H]^+$ = 457.0864 (Cacl.: $[M+H]^+$ = 457.0863, error = 0.2 p.p.m.) and the Hbt derivative: $[M+H]^+$ = 505.1509 (Cacl.: $[M+H]^+$ = 505.1522, error = 2.6 p.p.m.). The extracted ion chromatogram of the Lan derivative with a mass window between m/z 456.5-458.0 Da. A peak with the retention time of 8.2 min was identified as the Lan derivative. The extracted ion chromatogram of the Hbt derivative with a mass window between m/z 504.5-505.5 Da. A peak with the retention time of 6.3 min was identified as the Hbt derivative. The analysis by HPLC was on an ACQUITY UPLC C18 column (150 × 2.1 mm, 1.7 μm) by gradient elution of solvent A (H_2O with 0.1% formic acid) and solvent B (acetonitrile with 0.1% formic acid) with a flow rate of 0.2 mL/min over a 15 min period as follows: T = 0 min, 5% B; T = 2 min, 5% B; T = 12 min, 98% B; and T = 15 min, 98% B.

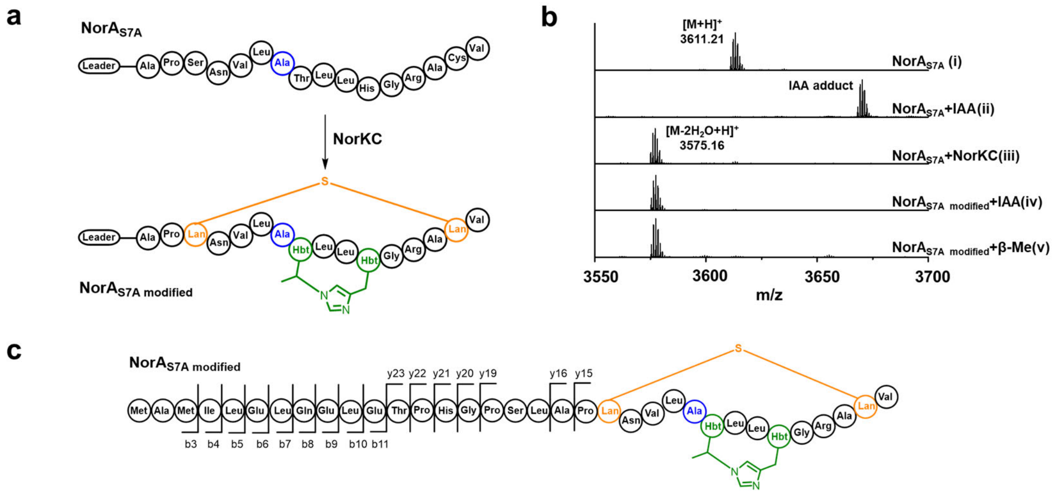

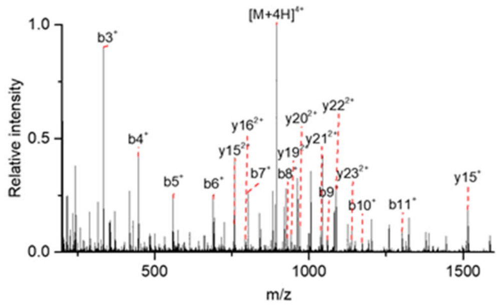

Species	Predicted Mass/Da	Observed Mass/Da	Error/ ppm	Species	Predicted Mass/Da	Observed Mass/Da	Error/ ppm
b3	334.1254	334.1250	1.2	y15	1514.8260	1514.8273	0.9
b4	447.2094	447.2084	2.2	$y15^{2+}$	757.9166	757.9179	1.7
b5	560.2935	560.2934	0.2	$y16^{2+}$	793.4352	793.4331	2.6
b6	689.3361	689.3354	1.0	$y19^{2+}$	942.0196	942.0196	0.0
b7	802.4201	802.4229	3.5	$y20^{2+}$	970.5303	970.5314	1.1
b8	930.4787	930.4785	0.2	$y21^{2+}$	1039.0598	1039.0641	4.1
b9	1059.5213	1059.5223	0.9	$y22^{2+}$	1087.5862	1087.5879	1.6
b10	1172.6054	1172.6018	3.1	$y23^{2+}$	1138.1100	1138.1107	0.6
b11	1301.6480	1301.6489	0.7				

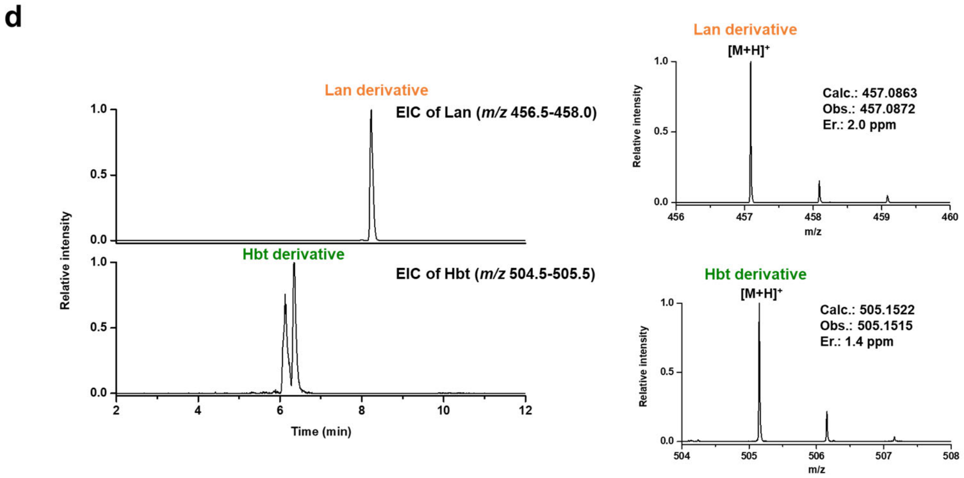

Supplementary Figure 45. Modification of NorA$_{S7A}$ by NorKC. (a) Modification of NorA$_{S7A}$ by NorKC. (b) MALDI-TOF-MS analysis of the modification of NorA$_{S7A}$ by NorKC *in vitro*: (i) 20 mM Tris-HCl buffer (pH 8.0), 5 mM ATP, 10 mM $MgCl_2$, 0.1 mM TCEP, 50 μM NorA$_{S7A}$, 10 μM NorKC. NorA$_{S7A}$: $M_{calc.}$ =3611.87 Da, $M_{obs.}$ =3611.21 Da. (ii) NorA$_{S7A}$ treated by IAA led to the formation of IAA adduct; (iii) NorA$_{S7A}$ incubated with NorKC (10 μM) at 28 °C for 1 h. NorA$_{S7A\ modified}$: $M_{calc.}$ =3575.85 Da, $M_{obs.}$

=3575.16 Da; (iv) $NorA_{S7A\ modified}$ treated by IAA did not result in any mass change; (v) $NorA_{S7A\ modified}$ treated by βME did not result in any mass change. (c) LC-MS/MS analysis of $NorA_{S7A\ modified}$. The *b* and *y* ions are listed in table and marked in the spectrum. (d) LC-MS analysis of the derivatized hydrolysate of $NorA_{S7A\ modified}$, and HRMS analysis of the Lan derivative: $[M+H]^+$ = 457.0872 (Cacl.: $[M+H]^+$ = 457.0863, error = 2.0 p.p.m.) and the Hbt derivative: $[M+H]^+$ = 505.1515 (Cacl.: $[M+H]^+$ = 505.1522, error = 1.4 p.p.m.). The extracted ion chromatogram of the Lan derivative with a mass window between m/z 456.5-458.0 Da. A peak with the retention time of 8.2 min was identified as the Lan derivative. The extracted ion chromatogram of the Hbt derivative with a mass window between m/z 504.5-505.5 Da. A peak with the retention time of 6.4 min was identified as the Hbt derivative. The analysis by HPLC was on an ACQUITY UPLC C18 column (150 × 2.1 mm, 1.7 μm) by gradient elution of solvent A (H_2O with 0.1% formic acid) and solvent B (acetonitrile with 0.1% formic acid) with a flow rate of 0.2 mL/min over a 15 min period as follows: T = 0 min, 5% B; T = 2 min, 5% B; T = 12 min, 98% B; and T = 15 min, 98% B.

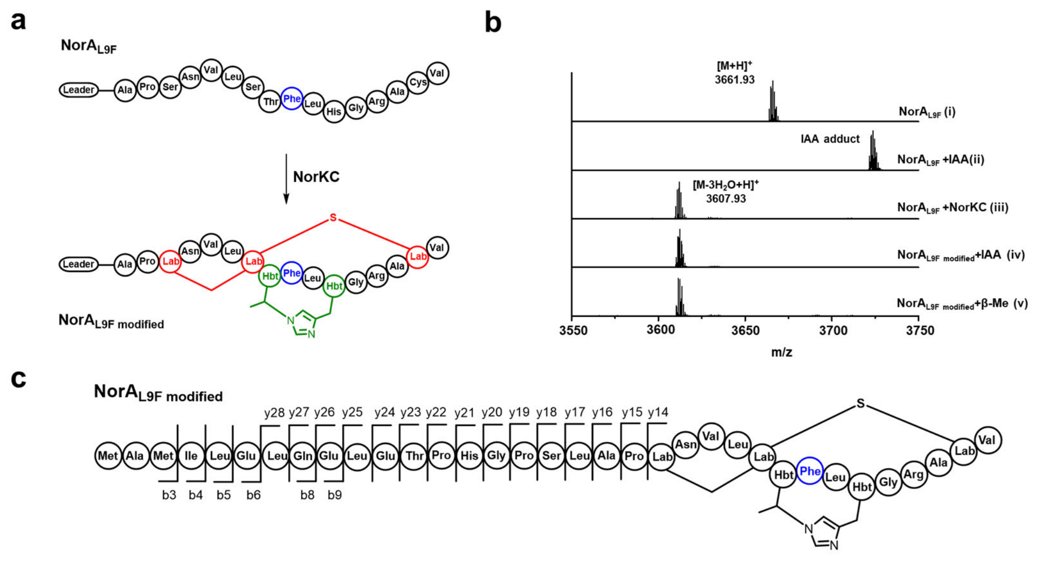

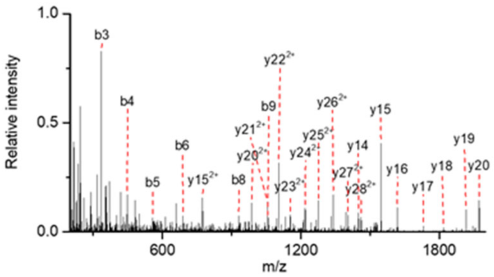

Species	Predicted Mass/Da	Observed Mass/Da	Error/ ppm	Species	Predicted Mass/Da	Observed Mass/Da	Error/ ppm
b3	334.1254	334.1245	2.7	y19	1915.0006	1914.9972	1.8
b4	447.2094	447.2064	6.7	y20	1972.0221	1972.0254	1.7
b5	560.2935	560.2915	3.6	y20^{2+}	986.5147	986.5165	1.8
b6	689.3361	689.3328	4.8	y21^{2+}	1055.0441	1055.0469	2.7
b8	930.4787	930.4750	4.0	y22^{2+}	1103.5705	1103.5723	1.6
b9	1059.5213	1059.5233	1.9	y23^{2+}	1154.0943	1154.0915	2.4
y14	1449.7419	1449.7395	1.7	y24^{2+}	1218.6156	1218.6162	0.5
y15	1546.7946	1546.7911	2.3	y25^{2+}	1275.1577	1275.1587	0.8
y16	1617.8317	1617.8301	1.0	y26^{2+}	1339.6790	1339.6781	0.7
y17	1730.9158	1730.9126	1.8	y27^{2+}	1403.7083	1403.7068	1.1
y18	1817.9478	1817.9459	1.0	y28^{2+}	1460.2503	1460.2489	1.0

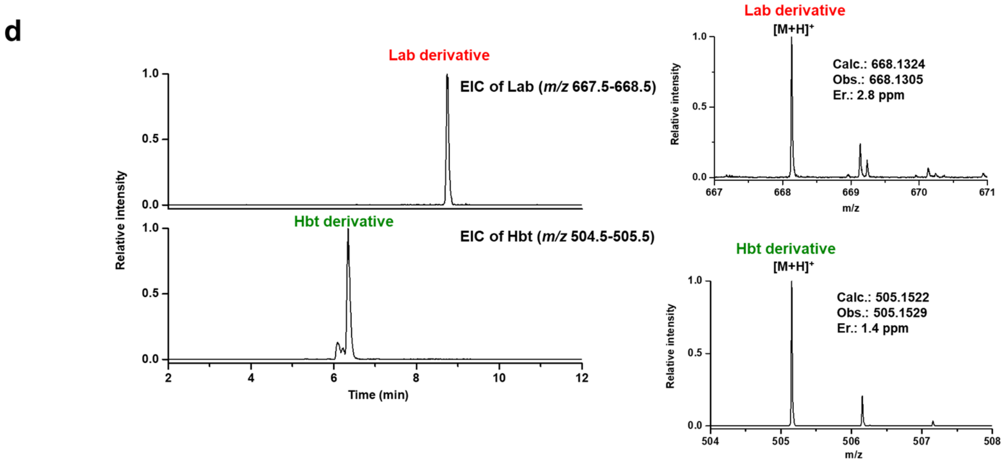

Supplementary Figure 46. NorKC catalyzes the formation of the Lab and the Hbt motif in the NorA$_{L9F}$ peptide. (a) Modification of NorA$_{L9F}$ by NorKC. (b) MALDI-TOF-MS analysis of the modification of NorA$_{L9F}$ by NorKC *in vitro*: (i) 20 mM Tris-HCl buffer (pH 8.0), 5 mM ATP, 10 mM $MgCl_2$, 0.1 mM TCEP, 50 μM NorA$_{L9F}$, 10 μM NorKC. NorA$_{L9F}$: $M_{calc.}$ =3661.85 Da, $M_{obs.}$ =3661.93 Da. (ii) NorA$_{L9F}$

treated by IAA led to the formation of IAA adduct; (iii) NorA$_{\text{L9F}}$ incubated with NorKC (10 μM) at 28 °C for 1 h. NorA$_{\text{L9F modified}}$: $M_{\text{calc.}}$ =3607.82 Da, $M_{\text{obs.}}$ =3607.93 Da; (iv) NorA$_{\text{L9F modified}}$ treated by IAA did not result in any mass change; (v) NorA$_{\text{L9F modified}}$ treated by βME did not result in any mass change. (c) LC-MS/MS analysis of NorA$_{\text{L9F modified}}$. The *b* and *y* ions are listed in table and marked in the spectrum. (d) LC-MS analysis of the derivatized hydrolysate of NorA$_{\text{L9F modified}}$, and HRMS analysis of the Lab derivative: $[M+H]^+$ =668.1305 (Cacl.: $[M+H]^+$ = 668.1324, error = 2.8 p.p.m.) and the Hbt derivative: $[M+H]^+$ = 505.1529 (Cacl.: $[M+H]^+$ = 505.1522, error = 1.4 p.p.m.). The extracted ion chromatogram of the Lab derivative with a mass window between m/z 667.5-668.5 Da. A peak with the retention time of 8.8 min was identified as the Lab derivative. The extracted ion chromatogram of the Hbt derivative with a mass window between m/z 504.5-505.5 Da. A peak with the retention time of 6.3 min was identified as the Hbt derivative. The analysis by HPLC was on an ACQUITY UPLC C18 column (150 × 2.1 mm, 1.7 μm) by gradient elution of solvent A (H_2O with 0.1% formic acid) and solvent B (acetonitrile with 0.1% formic acid) with a flow rate of 0.2 mL/min over a 15 min period as follows: T = 0 min, 5% B; T = 2 min, 5% B; T = 12 min, 98% B; and T = 15 min, 98% B.

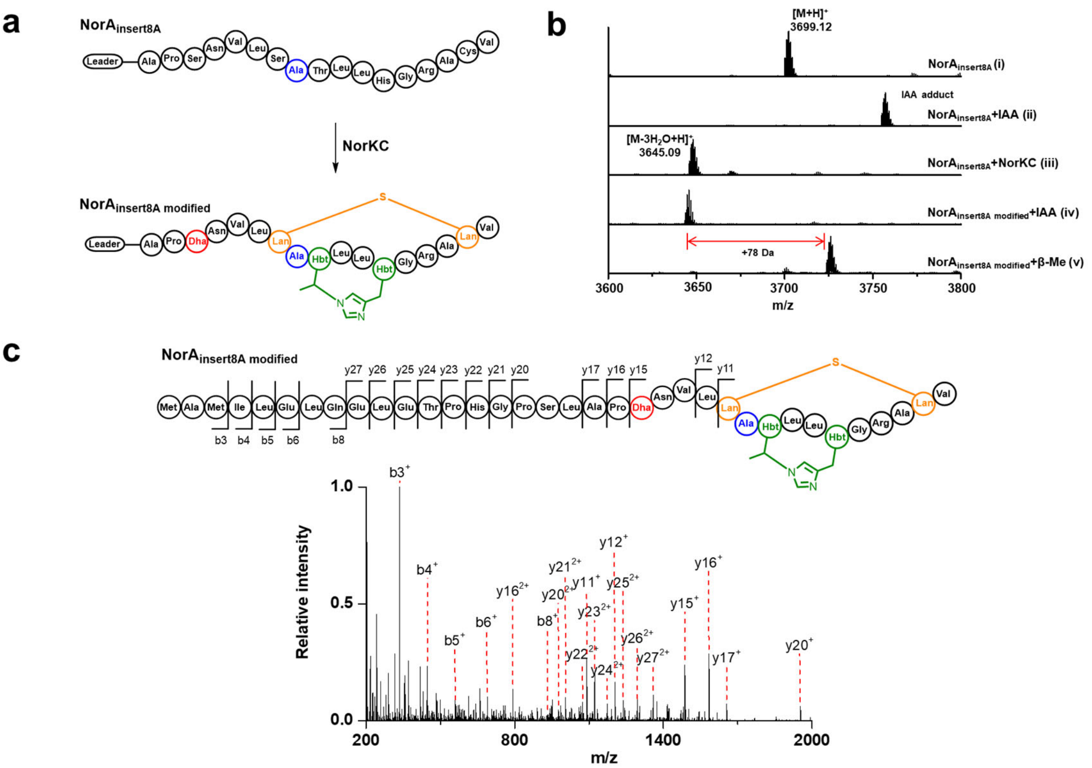

Species	Predicted Mass/Da	Observed Mass/Da	Error/ ppm	Species	Predicted Mass/Da	Observed Mass/Da	Error/ ppm
b3	334.1254	334.1261	2.1	y20	1952.0534	1952.0605	3.6
b4	447.2094	447.2100	1.3	$y16^{2+}$	792.4273	792.4241	4.0
b5	560.2935	560.2946	2.0	$y20^{2+}$	976.5303	976.5305	0.2
b6	689.3361	689.3367	0.9	$y21^{2+}$	1005.0411	1005.0452	4.1
b8	930.4787	930.4768	2.0	$y22^{2+}$	1073.5705	1073.5726	2.0
y11	1091.5778	1091.5824	4.2	$y23^{2+}$	1122.0969	1122.0941	2.5
y12	1204.6619	1204.6679	5.0	$y24^{2+}$	1172.6207	1172.6241	2.9
y15	1486.7946	1486.7998	3.5	$y25^{2+}$	1237.1421	1237.1478	4.6
y16	1583.8474	1583.8467	0.4	$y26^{2+}$	1293.6841	1293.6851	0.8
y17	1654.8845	1654.8897	3.1	$y27^{2+}$	1358.2053	1358.2101	3.5

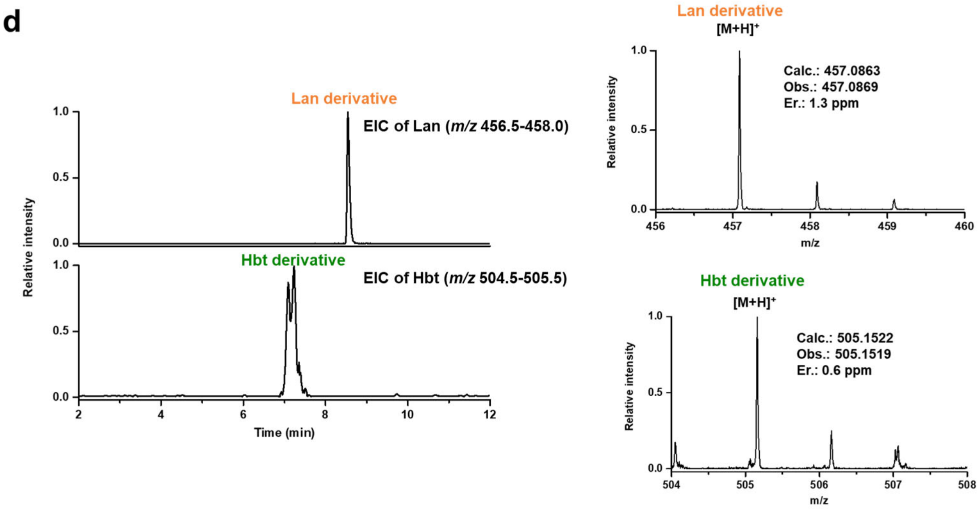

Supplementary Figure 47. NorKC catalyzes the formation of a Lan and a Hbt motif in the NorA$_{insert8A}$ peptide. (a) Modification of NorA$_{insert8A}$ by NorKC. (b) MALDI-TOF-MS analysis of the modification of NorA$_{insert8A}$ by NorKC *in vitro*: (i) 20 mM Tris-HCl buffer (pH 8.0), 5 mM ATP, 10 mM $MgCl_2$, 0.1 mM TCEP, 50 μM NorA$_{insert8A}$, 10 μM NorKC. NorA$_{insert8A}$: $M_{calc.}$ =3698.90 Da, $M_{obs.}$ =3699.12 Da. (ii) NorA$_{insert8A}$ treated by IAA led to the formation of IAA adduct; (iii) NorA$_{insert8A}$ incubated with NorKC (10 μM) at 28 °C for 1 h. NorA$_{insert8A\ modified}$: $M_{calc.}$ =3644.87 Da, $M_{obs.}$ =3645.09 Da; (iv) NorA$_{insert8A\ modified}$

treated by IAA did not result in any mass change; (v) $NorA_{insert8A\ modified}$ treated by β-Me led to the formation of β-Me adduct. (c) LC-MS/MS analysis of $NorA_{insert8A\ modified}$. The *b* and *y* ions are listed in table and marked in the spectrum. (d) LC-MS analysis of the derivatized hydrolysate of $NorA_{insert8A\ modified}$, and HRMS analysis of the Lan derivative: $[M+H]^+$ =457.0869 (Cacl.: $[M+H]^+$ = 457.0863, error = 1.3 p.p.m.) and the Hbt derivative: $[M+H]^+$ = 505.1519 (Cacl.: $[M+H]^+$ = 505.1522, error = 0.6 p.p.m.). The extracted ion chromatogram of the Lan derivative with a mass window between m/z 456.5-458.0 Da. A peak with the retention time of 8.7 min was identified as the Lab derivative. The extracted ion chromatogram of the Hbt derivative with a mass window between m/z 504.5-505.5 Da. A peak with the retention time of 7.2 min was identified as the Hbt derivative. The analysis by HPLC was on an ACQUITY UPLC C18 column (150 × 2.1 mm, 1.7 μm) by gradient elution of solvent A (H_2O with 0.1% formic acid) and solvent B (acetonitrile with 0.1% formic acid) with a flow rate of 0.2 mL/min over a 15 min period as follows: T = 0 min, 5% B; T = 2 min, 5% B; T = 12 min, 98% B; and T = 15 min, 98% B.

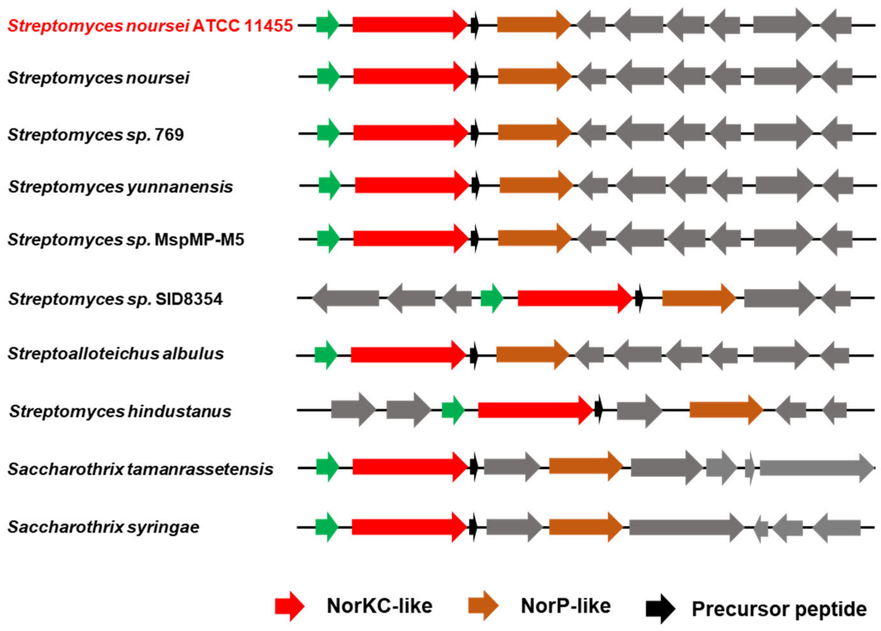

Supplementary Figure 48. BGCs homologous to the *nor* BGC found in bacterial genomes. The accession numbers of NorKC-like proteins as follow: WP_146051080.1 (*Streptomyces noursei*), AJC61591.1 (*Streptomyces sp.* 769), SHM76145.1 (*Streptomyces yunnanensis*), WP_158690164.1 (*Streptomyces sp.* MspMP-M5), WP_018539796.1 (*Streptomyces sp.* SID8354), WP_189866512.1 (*Streptomyces albulus*), WP_184696628.1 (*Saccharothrix tamanrassetensis*), WP_051766684.1 (*Saccharothrix syringae*).

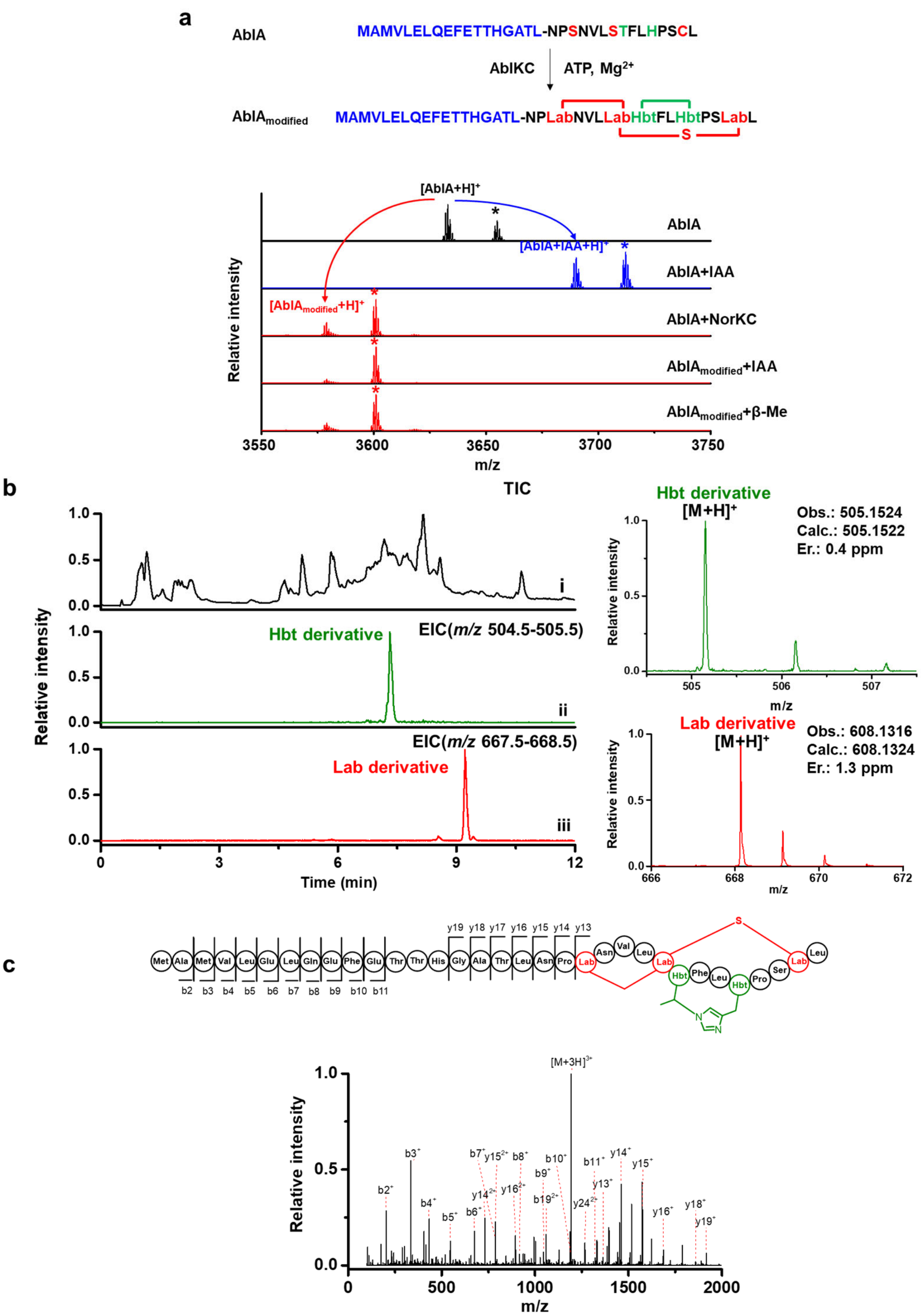

Species	Predicted Mass/Da	Observed Mass/Da	Error/ ppm	Species	Predicted Mass/Da	Observed Mass/Da	Error/ ppm
b2	203.0853	203.0849	2.0	y13	1363.6895	1363.6827	5.0
b3	334.1257	334.1254	0.9	y14	1460.7418	1460.7354	4.4
b4	433.1939	433.1938	0.2	y15	1574.7857	1574.7784	4.6
b5	546.2784	546.2779	0.9	y16	1687.8581	1687.8624	2.5
b6	675.3211	675.3205	0.9	y18	1859.9537	1859.9472	3.5
b7	788.4021	788.4045	3.0	y19	1916.9704	1916.9687	0.9
b8	916.4658	916.4631	2.9	$b19^{2+}$	1059.0057	1059.0058	0.1
b9	1045.5102	1045.5057	4.3	$y14^{2+}$	730.8707	730.8714	1.0
b10	1192.5711	1192.5741	2.5	$y15^{2+}$	787.8918	787.8928	1.3
b11	1321.6152	1321.6167	1.1	$y17^{2+}$	894.9544	894.9587	4.8

Supplementary Figure 49. AblKC catalyzes the formation of the Lab and the Hbt motif in the AblA peptide. (a) MALDI-TOF-MS analysis of the modification of AblA by AblKC. AblA: $M_{calc.}$=3631.33 Da, $M_{obs.}$=3630.92 Da. AblA$_{modified}$: $M_{calc.}$=3577.25 Da, $M_{obs.}$=3576.83 Da. [M+Na]$^+$ was labeled by *. Assay

conditions: 20 mM Tris-HCl buffer (pH 8.0), 1 mM ATP, 1 mM $MgCl_2$, 0.1 mM TCEP, 50 μM AblA and 10 μM AblKC at 28 °C for 1 h. (b) LC-MS analysis of the derivatized hydrolysate of $AblA_{modified}$, and HRMS analysis of the Hbt derivative: $[M+H]^+$ = 505.1524 (Cacl.: $[M+H]^+$ = 505.1522, error = 0.4 p.p.m.) and the Lab derivative: $[M+H]^+$ = 608.1316 (Cacl.: $[M+H]^+$ = 608.1324, error = 1.3 p.p.m.). (i) The total ion chromatography; (ii) The extracted ion chromatogram of the Hbt derivative with a mass window between m/z 504.5-505.5 Da. A peak with the retention time of 7.4 min was identified as the Hbt derivative. (iii) The extracted ion chromatogram of the Lab derivative with a mass window between m/z 667.5-668.5 Da. A peak with the retention time of 9.2 min was identified as the Lab derivative. The analysis by HPLC was on an ACQUITY UPLC C18 column (150 × 2.1 mm, 1.7 μm) by gradient elution of solvent A (H_2O with 0.1% formic acid) and solvent B (acetonitrile with 0.1% formic acid) with a flow rate of 0.2 mL/min over a 15 min period as follows: T = 0 min, 5% B; T = 2 min, 5% B; T = 12 min, 98% B; and T = 15 min, 98% B; (c) LC-MS/MS analysis of $AblA_{modified}$. The *b* and *y* ions are listed in table and marked in the spectrum.

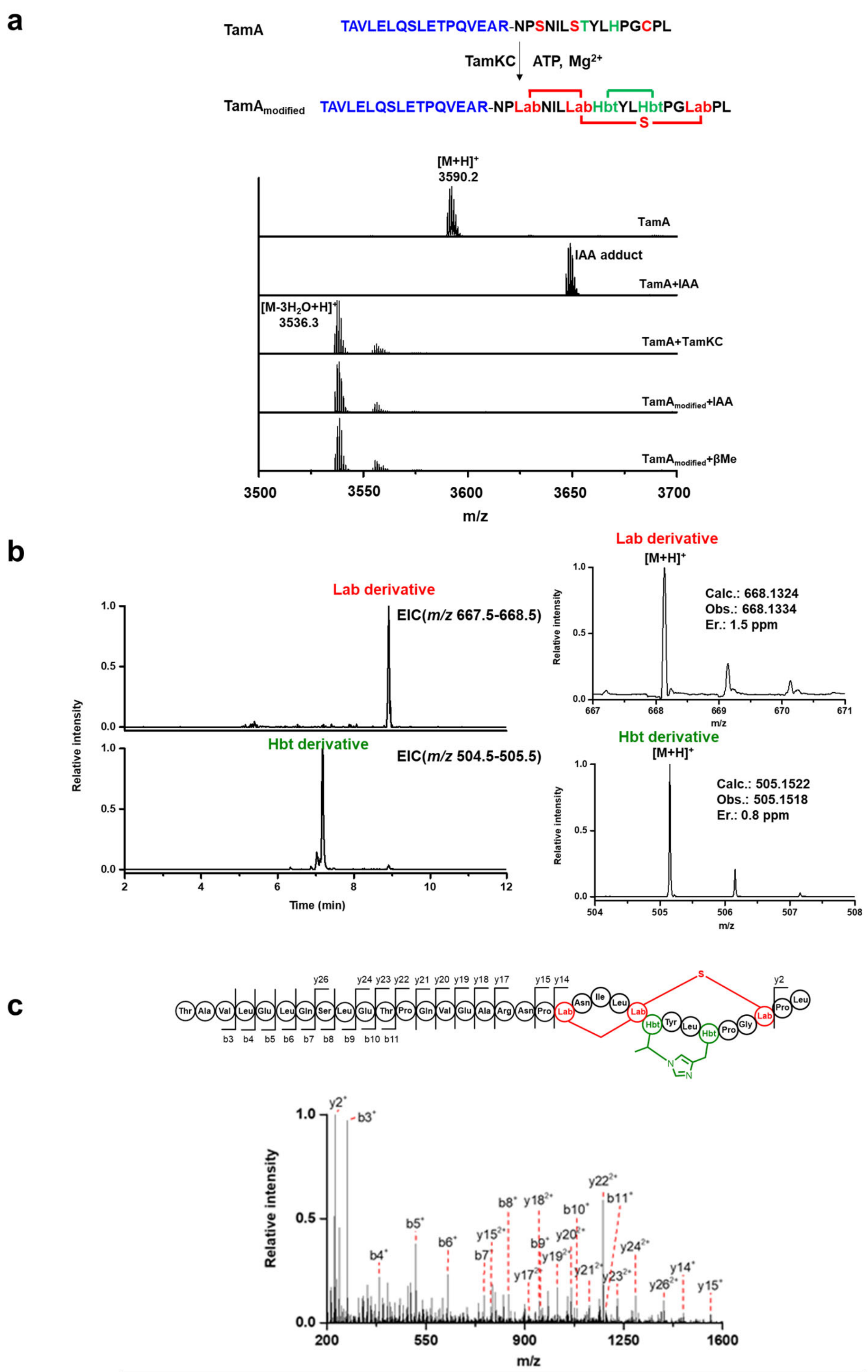

Species	Predicted Mass/Da	Observed Mass/Da	Error/ ppm	Species	Predicted Mass/Da	Observed Mass/Da	Error/ ppm
b3	272.1605	272.1601	1.5	y15	1557.7881	1557.7876	0.3
b4	385.2445	385.2439	1.6	$y15^{2+}$	779.3977	779.3943	4.4
b5	514.2871	514.2869	0.4	$y17^{2+}$	914.4697	914.4712	1.6
b6	627.3712	627.3706	1.0	$y18^{2+}$	949.9883	949.9879	0.4
b7	755.4298	755.4307	1.2	$y19^{2+}$	1014.5096	1014.5126	3.0
b8	842.4618	842.4645	3.2	$y20^{2+}$	1064.0438	1064.0455	1.6
b9	955.5459	955.5424	3.7	$y21^{2+}$	1128.0731	1128.0777	4.1
b10	1084.5885	1084.5878	0.6	$y22^{2+}$	1176.5995	1176.6015	1.7
b11	1185.6361	1185.6369	0.7	$y23^{2+}$	1227.1233	1227.1289	4.6
y2	229.1547	229.1543	1.7	$y24^{2+}$	1291.6446	1291.6498	4.0
y14	1460.7354	1460.7341	0.9	$y26^{2+}$	1391.7026	1391.7068	3.0

Supplementary Figure 50. TamKC catalyzes the formation of the Lab and the Hbt motif in the TamA

peptide. (a) MALDI-TOF-MS analysis of the modification of TamA by TamKC. TamA: $M_{calc.}$=3590.5 Da, $M_{obs.}$=3590.2 Da. $TamA_{modified}$: $M_{calc.}$=3536.2 Da, $M_{obs.}$=3536.3 Da. Assay conditions: 20 mM Tris-HCl buffer (pH 8.0), 1 mM ATP, 1 mM $MgCl_2$, 0.1 mM TCEP, 50 µM TamA and 10 µM TamKC at 28 °C for 1 h. (b) LC-MS analysis of the derivatized hydrolysate of $TamA_{modified}$, and HRMS analysis of the Hbt derivative: $[M+H]^+$ = 505.1518 (Cacl.: $[M+H]^+$ = 505.1522, error = 0.8 p.p.m.) and the Lab derivative: $[M+H]^+$ = 608.1334 (Cacl.: $[M+H]^+$ = 608.1324, error = 1.5 p.p.m.). The extracted ion chromatogram of the Hbt derivative with a mass window between m/z 504.5-505.5 Da. A peak with the retention time of 7.2 min was identified as the Hbt derivative. The extracted ion chromatogram of the Lab derivative with a mass window between m/z 667.5-668.5 Da. A peak with the retention time of 8.9 min was identified as the Lab derivative. The analysis by HPLC was on an ACQUITY UPLC C18 column (150 × 2.1 mm, 1.7 µm) by gradient elution of solvent A (H_2O with 0.1% formic acid) and solvent B (acetonitrile with 0.1% formic acid) with a flow rate of 0.2 mL/min over a 15 min period as follows: T = 0 min, 5% B; T = 2 min, 5% B; T = 12 min, 98% B; and T = 15 min, 98% B; (c) LC-MS/MS analysis of $TamA_{modified}$. The *b* and *y* ions are listed in table and marked in the spectrum.

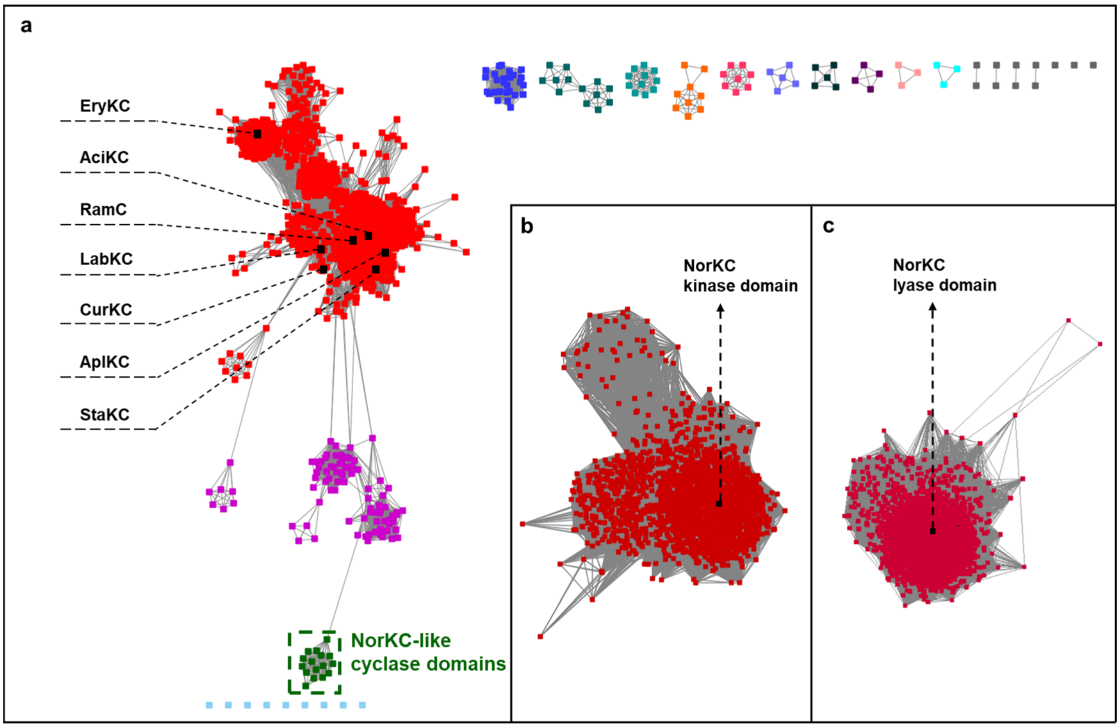

Supplementary Figure 51. The colored SSN for the cyclase domains (a), kinase domains (b), and lyase domains (c) of class III lanthipeptide synthases. The lyase, kinase and cyclase domains of NorKC were used as BLAST inputs to gather additional class III lanthipeptide synthases (n=2650). Sequences were analyzed by EFI-EST (E-Value: 5, Fraction: 1, Filter Value: 50) and visualized using Cytoscape.

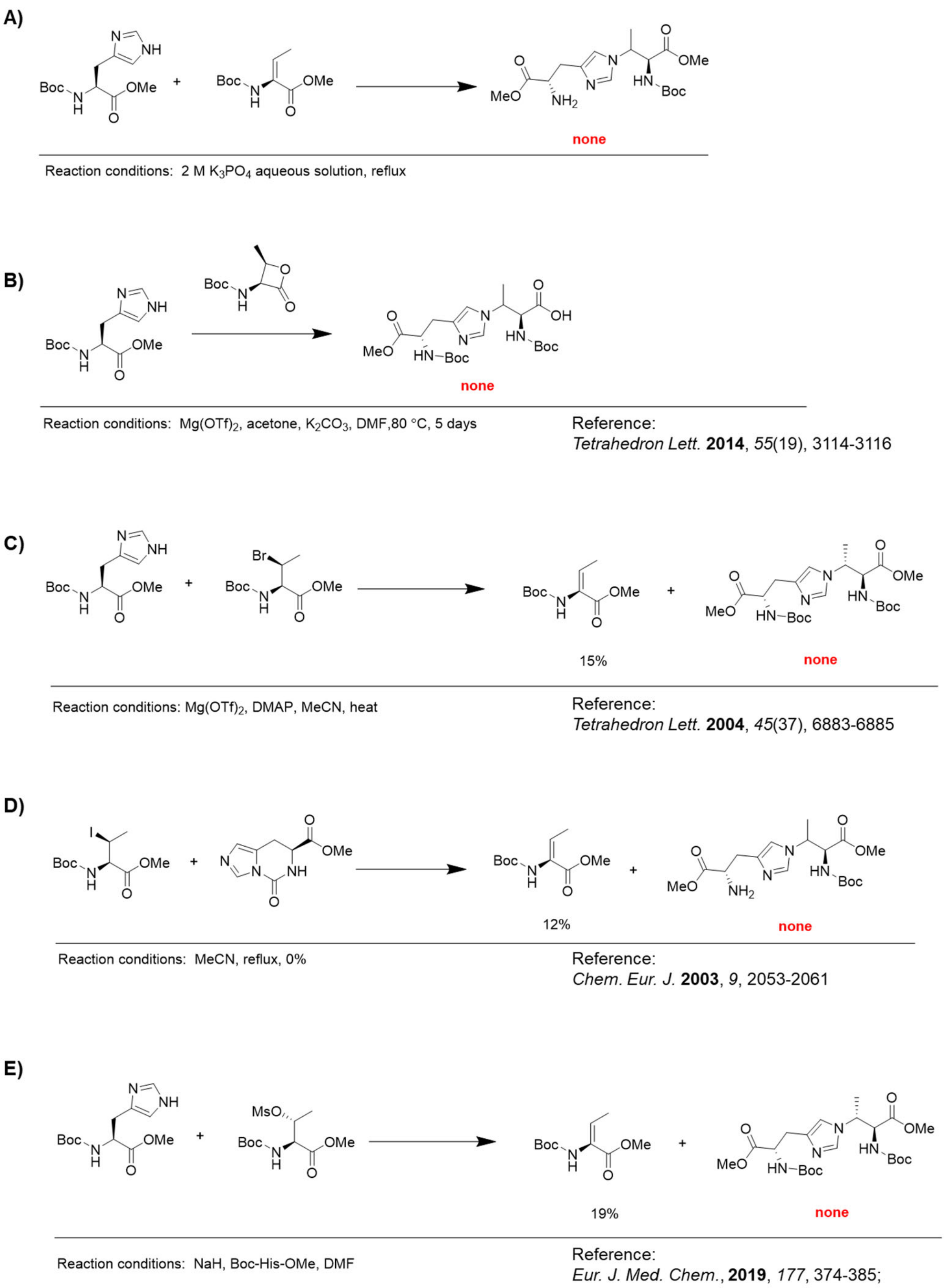

Supplementary Figure 52. Synthetic attempts to synthesize Hbt crosslinks. (A) Reaction of derivatives of His and Dhb was carried out in 2 M K_3PO_4 aqueous solution under reflux condition for 12 hours; (B)-(E) reactions were carried out following the reported procedures. All attempts failed to produce Hbt derivatives.
