# Supplementary material for: Discovery and biosynthesis of tricyclic copper-binding ribosomal peptides containing histidine-to-butyrine crosslinks
Source: Nat Commun. 2023 May 23;14:2944. doi: 10.1038/s41467-023-38517-2 (PMC10206099; doi:10.1038/s41467-023-38517-2)
Supplement: Supplementary file 3 — Reporting Summary [file 41467_2023_38517_MOESM3_ESM.pdf]

nature portfolio

Corresponding author(s): Jiangtao Gao, Hongwei Yao and Huan Wang

Last updated by author(s): Mar 7, 2023

Reporting Summary

Nature Portfolio wishes to improve the reproducibility of the work that we publish. This form provides structure for consistency and transparency in reporting. For further information on Nature Portfolio policies, see our Editorial Policies and the Editorial Policy Checklist.

Statistics

For all statistical analyses, confirm that the following items are present in the figure legend, table legend, main text, or Methods section.

n/a	Confirmed
☒	☐ The exact sample size (*n*) for each experimental group/condition, given as a discrete number and unit of measurement
☒	☐ A statement on whether measurements were taken from distinct samples or whether the same sample was measured repeatedly
☒	☐ The statistical test(s) used AND whether they are one- or two-sided<br>*Only common tests should be described solely by name; describe more complex techniques in the Methods section.*
☒	☐ A description of all covariates tested
☒	☐ A description of any assumptions or corrections, such as tests of normality and adjustment for multiple comparisons
☒	☐ A full description of the statistical parameters including central tendency (e.g. means) or other basic estimates (e.g. regression coefficient) AND variation (e.g. standard deviation) or associated estimates of uncertainty (e.g. confidence intervals)
☒	☐ For null hypothesis testing, the test statistic (e.g. *F*, *t*, *r*) with confidence intervals, effect sizes, degrees of freedom and *P* value noted<br>*Give P values as exact values whenever suitable.*
☒	☐ For Bayesian analysis, information on the choice of priors and Markov chain Monte Carlo settings
☒	☐ For hierarchical and complex designs, identification of the appropriate level for tests and full reporting of outcomes
☒	☐ Estimates of effect sizes (e.g. Cohen's *d*, Pearson's *r*), indicating how they were calculated

Our web collection on statistics for biologists contains articles on many of the points above.

Software and code

Policy information about availability of computer code

Data collection	Matrix-assisted laser desorption/ionization time-of-flight mass spectrometry (MALDI-TOF MS) was carried out on Bruker UltraFlextreme. Liquid chromatography electrospray ionization tandem mass spectrometry (LC/ESI-MS/MS) was carried out and processed using a Triple TOF 4600 System (AB Sciex) equipped with a Prominence Ultra-Fast Liquid Chromatography (UFLC) system (Shimadzu). UV-Vis spectrometry was recorded by NanoDrop 2000c (Thermo Scientific). Conditions for all ESI-MS and MS/MS were set as follows: nebulizer gas: 55 psi; heater gas: 55 psi; curtain gas: 35 psi; drying temperature: 550 °C; ion spray voltage: 5500 V; declustering potential: 100 V; collision energy: 35 V (positive); collision energy spread: 10 V. The mass range and accumulation time are 400-4000 m/z, 250 ms for ESI-MS and 100-2000 m/z, 100 ms for MS/MS, respectively. Collision-induced dissociation (CID) was performed for fragmentation of the respective peptide ions. Calibration solutions purchased from AB SCIEX were used for instrument calibration, and high resolution was chosen in the ESI+ mode. NMR experiments were performed at 298 K on Bruker AVANCE III 600 MHz and AVANCE NEO 800 MHz spectrometers equipped with 5 mm z-gradient 1H/13C/15N TCI cryogenic probes. Two-dimensional (2D) 1H-1H DQF-COSY, TOCSY, 1H-13C/15N HSQC, and 1H-13C HMBC were measured to obtain chemical shifts. 2D 1H-1H NOESY experiments with 500 ms of mixing time were performed to obtain 1H-1H distance constraints.
Data analysis	Matrix-assisted laser desorption/ionization-time of flight mass spectrometry data were analyzed by Bruker Daltonics Compass 1.4 for flexSeries. Liquid chromatography–mass spectrometry data were analyzed on PeakView Software (version 1.2). All NMR spectra were processed using TopSpin 4.1.1 and analyzed using NMRFAM-SPARKY. The chemical shifts for 1H were referenced to DSS, and 13C/15N chemical shifts were referenced indirectly. The Xplor-NIH program (version 3.5) was used for the structure determination and refinement. The 20 lowest energy structures were selected from 100 calculated structures for analysis. Figure generation was performed using PyMOL (version 2.5.0) and UCSF ChimeraX (version 1.2). Sequence similarity network (SSN) was generated using the EFI-EST tool (https://efi.igb.illinois.edu/efi-est/) with E-Value: 5, Fraction: 1, Filter Value: 50-200, and visualized using Cytoscape (version 3.7.2).

For manuscripts utilizing custom algorithms or software that are central to the research but not yet described in published literature, software must be made available to editors and reviewers. We strongly encourage code deposition in a community repository (e.g. GitHub). See the Nature Portfolio guidelines for submitting code & software for further information.

Data

Policy information about availability of data

All manuscripts must include a data availability statement. This statement should provide the following information, where applicable:

- Accession codes, unique identifiers, or web links for publicly available datasets
- A description of any restrictions on data availability
- For clinical datasets or third party data, please ensure that the statement adheres to our policy

The NMR structures of noursin and noursinH11W generated in this study have been deposited in the PDB database under accession numbers 7YFS (https://www.rcsb.org/structure/unreleased/7YFS) and 8HZW (https://www.rcsb.org/structure/unreleased/8HZW), respectively. The accession numbers of NorKC, AblKC and TamKC proteins from NCBI database are ANZ21440.1 (https://www.ncbi.nlm.nih.gov/protein/ANZ21440.1), WP_189866512.1 (https://www.ncbi.nlm.nih.gov/protein/WP_189866512.1/) and WP_184696628.1 (https://www.ncbi.nlm.nih.gov/protein/WP_184696628.1), respectively. Supplementary information is available for this paper online. Correspondence and requests for materials should be addressed to H.W., H.Y. or J.G..

Human research participants

Policy information about studies involving human research participants and Sex and Gender in Research.

Reporting on sex and gender	N/A
Population characteristics	N/A
Recruitment	N/A
Ethics oversight	N/A

Note that full information on the approval of the study protocol must also be provided in the manuscript.

Field-specific reporting

Please select the one below that is the best fit for your research. If you are not sure, read the appropriate sections before making your selection.

☒ Life sciences ☐ Behavioural & social sciences ☐ Ecological, evolutionary & environmental sciences

For a reference copy of the document with all sections, see nature.com/documents/nr-reporting-summary-flat.pdf

Life sciences study design

All studies must disclose on these points even when the disclosure is negative.

Sample size	Genome mining of bacterial genomes led to the identification of ten homologous BGCs. Two enzymes, AlbKC and TamKC that share 96% and 59% sequence similarity with NorKC, respectively, were chosen as model NorKC homologs to verify their function. We believe that this sample size is sufficient to demonstrate the correlation between domain interactions and catalytic activities of these enzymes.
Data exclusions	No data were excluded from analysis.
Replication	All replication of experiments were successful. Since there is no quantitative results presented in this paper, the mass spectrometry analysis data is representative of all replications.
Randomization	n/a (Each biochemical experiment performed in this study is rationally designed and leads to a specific conclusion. Samples were not randomized)
Blinding	n/a (Each biochemical experiment performed in this study is rationally designed and leads to a specific conclusion. Experiments were not blinded.)

Reporting for specific materials, systems and methods

We require information from authors about some types of materials, experimental systems and methods used in many studies. Here, indicate whether each material, system or method listed is relevant to your study. If you are not sure if a list item applies to your research, read the appropriate section before selecting a response.

Materials & experimental systems

n/a	Involved in the study
☒	☐ Antibodies
☒	☐ Eukaryotic cell lines
☒	☐ Palaeontology and archaeology
☒	☐ Animals and other organisms
☒	☐ Clinical data
☒	☐ Dual use research of concern

Methods

n/a	Involved in the study
☒	☐ ChIP-seq
☒	☐ Flow cytometry
☒	☐ MRI-based neuroimaging
